# City-wide electronic health records reveal gender and age biases in administration of known drug–drug interactions

**DOI:** 10.1038/s41746-019-0141-x

**Published:** 2019-07-23

**Authors:** Rion Brattig Correia, Luciana P. de Araújo Kohler, Mauro M. Mattos, Luis M. Rocha

**Affiliations:** 1 School of Informatics, Computing & Engineering, Indiana University, Bloomington, IN 47408 USA; 2 CAPES Foundation, Ministry of Education of Brazil, Brasília, DF 70040-020 Brazil; 3 Instituto Gulbenkian de Ciência, Oeiras, 2780-156 Portugal; 4 Universidade Regional de Blumenau (FURB), Blumenau, SC 89030-903 Brazil

**Keywords:** Risk factors, Drug regulation, Epidemiology, Computational science, Public health

## Abstract

The occurrence of drug–drug-interactions (DDI) from multiple drug dispensations is a serious problem, both for individuals and health-care systems, since patients with complications due to DDI are likely to reenter the system at a costlier level. We present a large-scale longitudinal study (18 months) of the DDI phenomenon at the primary- and secondary-care level using electronic health records (EHR) from the city of Blumenau in Southern Brazil (pop. ≈340,000). We found that 181 distinct drug pairs known to interact were dispensed concomitantly to 12% of the patients in the city’s public health-care system. Further, 4% of the patients were dispensed drug pairs that are likely to result in major adverse drug reactions (ADR)—with costs estimated to be much larger than previously reported in smaller studies. The large-scale analysis reveals that women have a 60% increased risk of DDI as compared to men; the increase becomes 90% when considering only DDI known to lead to major ADR. Furthermore, DDI risk increases substantially with age; patients aged 70–79 years have a 34% risk of DDI when they are dispensed two or more drugs concomitantly. Interestingly, a statistical null model demonstrates that age- and female-specific risks from increased polypharmacy fail by far to explain the observed DDI risks in those populations, suggesting unknown social or biological causes. We also provide a network visualization of drugs and demographic factors that characterize the DDI phenomenon and demonstrate that accurate DDI prediction can be included in health care and public-health management, to reduce DDI-related ADR and costs.

## Introduction

Adverse drug reactions (ADR) from drug–drug interactions (DDI) is a well-known public health problem worldwide.^1–3^ Most efforts to measure the scale of ADR from DDI focus on hospitalizations and emergency visits^4–10^ or literature meta-analysis.^3,11,12^ Very few studies so far have been able to characterize this problem in primary and secondary-care settings. Lack of access to longitudinal data from Electronic Health Records (EHR) of large populations continues to be the main barrier to measuring the prevalence of DDI and characterizing the phenomenon in medical care.^13–15^ For instance, Molden et al.^16^ searched 43,500 patients in pharmacy databases in southeastern Norway, studying only DDI from CYP inhibitor-substrate drugs. Pinto et al.^17^ studied DDI prevalence in a small cohort of forty elderly hypertensive patients in a primary health care unit in Brazil. Iyer et al.^18^ mined 50 million clinical notes from the private EHR database STRIDE,^19^ to identify signals of unknown potential DDI from clinical text. While STRIDE contains EHR from multiple care levels, this analysis did not address the concomitant dispensation of pairs of drugs with known DDI in primary- and secondary-care. Lastly, Guthrie et al.^20^ performed a repeated cross-sectional comparison of 84 days in 1995 and 2010, to study the increase in polypharmacy and DDI at the primary- and secondary-care level in the Tayside region of Scotland (pop. 405,721); DDI was defined according to the *British National Formulary*, a private publication. This study estimated that 13% of adults (≥20 years old) were prescribed a “potentially serious” known DDI in 2010, and that the number of drugs prescribed was the characteristic most predictive of DDI. Patients prescribed 15 or more drugs had an almost 27 fold DDI risk increase over those prescribed two to four drugs. However, by using only 84-day windows, this analysis misses potential co-administrations from separate prescriptions made outside of the relatively short windows; it also analyzed prescription, rather than dispensation data.

Here we pursue a large-scale longitudinal study of the DDI phenomenon at the primary- and secondary-care levels in an entire city, using considerably larger time-windows and relying on public DDI and ADR standards. We obtained 18 months of EHR data for the city of Blumenau in Southern Brazil (pop. 338,876), a city with a very high Human Development Index (HDI = 0.806^21^)—at the level of the top quartile of countries according to this United Nations Development Programme index.^22^ Brazil has a universal public health-care system, and Blumenau possesses a city-wide Health Information System (HIS) with prescription and dispensation information for its entire population. The analysis of Blumenau’s EHR data is thus an opportunity to understand the DDI phenomenon in a highly developed city in a country where DDI is known to occur similarly to other nations.^10,11^ The study provides an understanding of both prevalence and bias in the dispensation of known DDI outside of hospital settings. Dispensation data are only a surrogate for administration of DDI, as we are not certain that patients actually take the medications that are dispensed concomitantly. However, dispensation data can only be a better surrogate of administration than prescription data that was used in previous studies (e.g., ref. ^20^), as a prescription may ultimately not be dispensed.

From a public-health perspective, the concomitant administration of drugs with adverse interactions is of great concern.^5,10,11^ Since over 30% of all ADR are thought to be caused by DDI,^18^ better identification and prediction of administration of known DDI in primary- and secondary-care could reduce the number of patients seeking urgent care in hospitals, resulting in substantial savings for health systems worldwide.^3,7,14^ A systematic review from 2009 showed that the proportion of hospital inpatients with ADR (in general, not DDI only) ranged from 1.6 to 41.4%.^11^ Furthermore, an estimated 52% (45%) of ADR in outpatients (inpatients) were preventable.^12^ In the elderly population alone (>65 years old), the yearly financial burden of ADR was estimated to reach $11.9 million for the province of Ontario (pop. 12M),^9^ or about $1 per capita, per year. As we report below, the yearly cost of major DDI estimated from the Blumenau EHR dispensation data for the same age group is higher, at least $2 per capita, per year, after adjusting for inflation and exchange rates—though for less stringent assumptions it can be as high as $7 per capita, per year. This suggests that the financial burden of DDI is more severe than previously thought. Moreover, the rate of major DDI found to be dispensed in Blumenau is smaller than what was reported to be prescribed in Scotland.^20^ Therefore the financial burden of DDI is likely higher in other health-care systems, especially those with older populations.

To characterize the significant factors in DDI, we study demographic variables such as gender and age, as well as the drugs involved in DDI in greater detail, and reveal previously unknown factors in this phenomenon. We show that women in Blumenau are at a greater risk of being dispensed known DDI than men, with a 1.6 risk multiplier. This increased risk for females is not confounded by the larger number of women present in the data nor their age. The analysis also identifies the drug pairs that most lead to DDI in women which, surprisingly, are not attributable to female-specific medicines (e.g., hormone therapy). We also demonstrate that there is a significant increase of DDI risk with age, reaching more than 30% for adults over 65 years of age. Importantly, using a statistical null model, we show that the age risk growth is not explained simply by the increase in polypharmacy in older age. This suggests that the specific drugs dispensed to older populations are more prone to DDI and/or that insufficient attention is paid to this phenomenon in primary care for this population.

While the number of drugs dispensed and the number of concomitant drug dispensations are the best predictors of DDI (previously only observed for number of drugs prescribed^20^), we show that these quantities by themselves are poor predictors of DDI. We look at demographic variables such as education and neighborhood affluence and show they do not play a significant role in the risk for DDI in our data. Other factors, however, play very significant roles, chiefly age, gender, and the specific drugs dispensed. Indeed, we demonstrate that the automatic prediction of which patients are dispensed known DDI is quite accurate when those factors are included. This makes decision-support systems for predicting DDI risk in HIS not only feasible, but necessary to lower the rates of known DDI being dispensed.

To better understand which drugs are most involved in the DDI phenomenon, we integrate all DDI information of the Blumenau population into easy-to-visualize DDI networks. Looking at gender differences, for example, analysis of these networks identifies key drugs and interactions in the DDI phenomenon, and demonstrates that the higher DDI risk women face is not associated with any type of hormone therapy. Indeed, drugs that most contribute to the gender-disparity in DDI risk are not female-specific. This suggests there may be social or biological processes at play in primary- and secondary-care that lead to increased DDI risk for women. A full listing of the drugs that most contribute to the DDI observed in our study are presented in our DDI network analysis and accompanying tables.

## Results

### DDI demographics, severity, and cost

Our analysis tallied Ψ = 1,025,754 distinct drug pair co-administrations. Almost 3% of these, or Φ = 26,524, are known DDI and involve 75 distinct drugs that participate in |Δ| = 181 observed distinct interaction drug pairs. There is very strong linear relationship between volume of drug dispensation (*α*^*N*^) and DDI (Φ^*N*^) across neighborhoods (*N*), which fits a regression line almost perfectly (*R*^2^ = 0.92, *p* < 10^−6^); see Supplementary Fig. 12-right. The distribution of these DDI pairs per severity class is detailed in Table 1. A majority (69%) are labeled “moderate”, although, worryingly, 22.5% are classified as “major” DDI. The observed DDI pairs were dispensed to |*U*^Φ^| = 15,527 unique patients, which represent 12% of the *Pronto* patient population (and almost 5% of the entire Blumenau population). Looking only at the adult *Pronto* population, this number is raised to 15% (15,336). Almost 4% of all *Pronto* patients (5.01% of adults) were administered a major DDI, and 9.58% (12.15% of adults) were administered a moderate DDI; these numbers represent 1.54 and 3.75% of the entire Blumenau population, respectively. See Methods for precise definitions of symbols and formulae used in this section.

**Table 1:** Number and proportions of DDI observations and affected patients per DDI severity class

Severity *s*	Φ	|*U*^Φ^|	|*U*^Φ^|/|*U*|	|*U*^Φ^|/Ω	|*U*^Φ,[*y* > 20]^|/|*U*^[*y* > 20]^|
Major	5,968 (22.50%)	5,224	3.94%	1.54%	5.01%
Moderate	18,335 (69.13%)	12,711	9.58%	3.75%	12.15%
Minor	542 (2.04%)	528	0.4%	0.16%	0.51%
n/a	1,679 (6.33%)	1,493	1.12%	0.44%	1.43%
Major ∨ moderate	24,303 (91.63%)	15,030	11.32%	4.44%	14.35%
Moderate ∨ minor	18,877 (71.17%)	12,791	9.64%	3.77%	12.22%

Drugs or interactions identified in *DrugBank* but not present in *Drugs.com* are tallied as n/a, see SM for details. First column: Φ, number and proportion of observed DDI co-administrations. Second column: |*U*^Φ^|, number of patients affected by at least one DDI. Third and fourth columns: proportion of patients from the *Pronto* system and entire Blumenau populations, respectively. Fifth column: proportion of adult patients (*y* ≥ 20 years old) from the pronto system. ∨ denotes the logical disjunction. Notice that the same patient may have been administered DDI of more than one severity class

We estimate the financial burden of DDI to Blumenau by evaluating how many of the 24,592 hospital admissions billed to this public health system in the same period^23^ were due to ADR from DDI. This estimation relies on conjecturing what proportion (*p*_*h*_) of patients who where dispensed a major DDI are likely to have an ADR that requires hospitalization (details in Supplementary Information §9). We focus on the most conservative value from available literature^3^, which yields *p*_*h*_ = 2.68%, as well as on a less conservative estimate also previously reported^9^ of *p*_*h*_ = 8.35%. The most conservative estimate leads to a cost of DDI-related hospitalization in Blumenau of over $1M in the 18-month period, or a per capita cost of $2.03. The extrapolated costs to the state and the country are $21M and $565M, respectively (see Supplementary Tables 34 and 35). The less conservative estimate reaches a per capita cost of $6.33, or $3.2M, $61M, and $1.5B, for the city, state and country levels, respectively. However, all of these conjectures are likely to err on the side of under-reporting emergency room admissions due to DDI or ADR, since this is a well-known problem in studies of this phenomenon.^24–27^ Therefore, in Supplementary Information we also report cost estimates for various values of *p*_*h*_, so that readers can judge what is an appropriate value to consider.

### Drugs Involved in Interactions

Table 2 lists the top 20 DDI pairs, ordered by the rank product of their strength of DDI association, $\tau _{i,j}^{\mathrm{\Phi }}$, with the number of patients they were administered to, $|U_{i,j}^{\mathrm{\Phi }}|$. The complete list of DDI pairs, including the severity class and other measures, is provided in Supplementary Table 7 ordered by the number of affected patients (see also Supplementary Note 7). *τ*_*i*,*j*_ is largest (smallest) for DDI pairs (*i*, *j*) that are more (less) likely to be co-administered when either one of drugs *i* or *j* is administered. Computing the rank product between $\tau _{i,j}^{\mathrm{\Phi }}$ and $|U_{i,j}^{\mathrm{\Phi }}|$ identifies DDI pairs that are very prevalent in the population but which also tend to be co-administered.

**Table 2:** Top 20 known DDI pairs

Rankp(*τ*,*U*)	$\tau _{i,j}^{\mathrm{\Phi }}$	$|U_{i,j}^{\mathrm{\Phi }}|$	$\langle \lambda _{i,j}^u\rangle$	*i*	*j*	$RRI_{i,j}^F$	Class
(2,4)	0.60	1249	141 ± 124	ASA	Glyburide	0.89	Moderate
(1,12)	0.70	524	243 ± 188	Haloperidol	Biperiden	0.62	Moderate
(4,11)	0.58	535	152 ± 132	Atenolol	Glyburide	1.22	Moderate
(3,17)	0.60	385	155 ± 125	Digoxin	Furosemide	0.61	Moderate
(62,1)	0.26	5078	102 ± 95	Omeprazole	Clonazepam	2.28	Moderate
(8,16)	0.55	470	160 ± 133	Diltiazem	Simvastatin	1.27	Major
(26,5)	0.45	1190	127 ± 127	Amitriptyline	Fluoxetine	3.55	Major
(82,2)	0.23	2117	53 ± 74	ASA	Ibuprofen	1.42	Major
(10,22)	0.55	272	140 ± 114	Digoxin	Spironolactone	0.58	Minor
(5,46)	0.57	95	140 ± 126	Propranolol	Glyburide	1.61	Moderate
(15,18)	0.50	377	143 ± 138	Fluoxetine	Carbamazepine	0.98	Moderate
(91,3)	0.21	1460	54 ± 77	Atenolol	Ibuprofen	1.88	Moderate
(61,6)	0.27	999	87 ± 86	Omeprazole	Diazepam	1.21	Moderate
(16,26)	0.49	226	151 ± 145	Amitriptyline	Carbamazepine	0.99	Moderate
(6,84)	0.56	25	157 ± 136	Diltiazem	Amiodarone	1.26	Major
(12,47)	0.52	91	154 ± 142	Atenolol	Diltiazem	1.19	Major
(21,27)	0.47	222	148 ± 139	Fluoxetine	Lithium	1.79	Major
(40,15)	0.36	496	103 ± 87	ASA	Gliclazide	0.78	None
(96,7)	0.20	892	56 ± 61	Fluconazole	Simvastatin	2.63	Major
(14,48)	0.50	90	161 ± 157	Imipramine	Carbamazepine	1.35	Moderate

Top 20 known DDI pairs (*i*, *j*) by rank product (first column; individual rank in parenthesis) of the ranks of $\tau _{i,j}^{\mathrm{\Phi }}$, the strength of DDI association from Eq. (4), and $|{U_{i,j}^{\mathrm{\Phi }}}|$, the number of patients affected by the DDI (second and third columns, respectively). Mean (±s.d.) co-administration length, $\langle {\lambda _{i,j}^u} \rangle$, is shown in column 4 (in days) for each DDI pair (*i*, *j*) whose English drug names are shown in columns 5 and 6. Relative gender risk of DDI pair co-administration, $RRI_{i,j}^F$ is shown in column 7. DDI severity classification, according to Drugs.com, shown in column 8, with DDIs not found in Drugs.com labeled as None

Only 2% of the observed DDI administrations are considered of *minor* risk, affecting 542 patients. The highest ranked one (nineth) in Table 2 is (Digoxin, Spironolactone) and it was administered to $|U_{i,j}^{\mathrm{\Phi }}| = 272$ patients (for $\langle \lambda _{i,j}^u\rangle = 140$ days on average); it leads to increased levels of Digoxin while decreasing the effect of Spironolactone. The vast majority (almost 70% per Table 1) of observed DDI administrations fall in the *moderate* risk class. For instance, (Digoxin, Furosemide) can cause “possible electrolyte variations and arrhythmia” (4th, $|U_{i,j}^{\mathrm{\Phi }}| = 385$, $\langle \lambda _{i,j}^u\rangle$ = 155). Others, like the pair (Haloperidol, Biperiden; second, $|U_{i,j}^{\mathrm{\Phi }}| = 524$, $\langle \lambda _{i,j}^u\rangle = 243$) give rise to various ADR, such as central nervous system depression and tardive dyskinesia; despite the known ADR this pair has been used clinically,^28^ which explains the large value of $\tau _{i,j}^{\mathrm{\Phi }} = 0.7$, meaning that these drugs are more likely to be co-administered. In hot weather this DDI increases the risk of hyperthermia and heat stroke, and Blumenau has a humid subtropical climate with temperatures reaching 30 °C with 100% humidity during summer.

(Omeprazole, Clonazepam) is the most frequent DDI pair observed, by a large margin to the second (fifth, $|U_{i,j}^{\mathrm{\Phi }}| = 5078$, $\langle \lambda _{i,j}^u\rangle = 102$). Omeprazole is used to treat acid reflux and other gastroesophageal problems, while Clonazepam is a benzodiazepine anti-epileptic. This prevalent dispensation requires particular attention to dosage since “Omeprazole may increase the pharmacological effect and serum levels of certain benzodiazepines via hepatic enzyme inhibition”.^28,29^ Similarly, (Acetylsalicylic Acid (ASA), Glyburide) is the top ranked pair in Table 2 and very frequently dispensed (1st, $|U_{i,j}^{\mathrm{\Phi }}| = 1249$, $\langle \lambda _{i,j}^u\rangle = 141$). This pair is especially problematic for diabetic patients since “the salicylate increases the effect of sulfonylurea;” It causes hypoglycemia by enhancing insulin sensitivity, particularly in patients with advanced age and/or renal impairment.^28,30^

*Major* DDI pairs represent 22.5% of all observed DDI administrations per Table 1. The top 20 major DDI pairs are listed in Supplementary Table 11 and include:

- (Diltiazem, Simvastatin), sixth, $|U_{i,j}^{\mathrm{\Phi }}| = 470$, $\langle \lambda _{i,j}^u\rangle = 160$, where “Diltiazem increases the effect and toxicity of simvastatin” possibly causing liver damage as a side effect.^31^
- (Fluoxetine, Amitriptyline), seventh, $|U_{i,j}^{\mathrm{\Phi }}| = 1190$, $\langle \lambda _{i,j}^u\rangle = 127$, where “Fluoxetine increases the effect and toxicity of tricyclics”.^32^ The same ADR affects (Fluoxetine, Imipramine), 23rd, $|U_{i,j}^{\mathrm{\Phi }}| = 257$, and (Fluoxetine, Nortriptyline), thirty-third, $|U_{i,j}^{\mathrm{\Phi }}| = 154$.
- (ASA, Ibuprofen), eighth, $|U_{i,j}^{\mathrm{\Phi }}| = 2117$, $\langle \lambda _{i,j}^u\rangle = 53$, where “Ibuprofen reduces ASA cardioprotective effects”. In 2015 the European Medicines Agency issued an updated advice that occasional use of Ibuprofen should not affect the benefits of low-dose ASA.^33^ Our analysis shows that patients were dispensed this pair concomitantly on average for 53 days (±74 s.d.), conflicting with occasional use. However, since these are common medications we cannot rule out the possibility they were dispensed to be taken as needed.
- (Fluoxetine, Lithium), seventeenth, $|U_{i,j}^{\mathrm{\Phi }}| = 222$, $\langle \lambda _{i,j}^u\rangle = 148$), where “the SSRI increases serum levels of lithium” potentiating the risk of serotonin syndrome, which is rare but serious and potentially fatal.^28,34^
- (Fluconazole, Simvastatin), nineteenth, $|U_{i,j}^{\mathrm{\Phi }}| = 892$, $\langle \lambda _{i,j}^u\rangle = 56$), which leads to “increased risk of myopathy/rhabdomyolysis”. Also from the azole class, Ketoconazole and Itraconazole are considered potent inhibitors generally causing less clinically significant interactions with Simvastatin than Fluconazole.^28^ Both substitutes are available free of charge in the public health care system.^35^

In addition, the top 20 DDI pairs ranked by a normalized drug “footprint” in the population are listed in Supplementary Table 12.

### Gender risk and DDI networks

The set of patients who were co-administered known DDI was comprised of |*U*^Φ,M^| = 4, 793 (30.54%) males and |*U*^Φ,F^| = 10, 734 (69.46%) females (see Supplementary Fig. 4). To understand whether this difference in the proportion of DDI per gender was due to *Pronto* having more female patients (59%), or because women tend to be prescribed more drugs in general,^36^ we computed two measures of relative risk of for women. The relative risk of co-administration (*RRC*) for women is *RRC*^F^ = 1.0653 while their relative risk of interaction (*RRI*) is *RRI*^F^ = 1.5864. If the risks were equivalent for both genders, we would observe *RRC*^M^ ≈ *RRC*^F^ ≈ 1 and *RRI*^M^ ≈ *RRI*^F^ ≈ 1. While the relative risk of drug co-administration is only slightly larger (≈7%) for females, the relative risk of drug interaction is much larger (≈59%). This risk becomes even higher when we look only at the most dangerous severity class: $RRI_{{\mathrm{major}}}^{\mathrm{F}} = 1.8739$, while $RRI_{{\mathrm{minor}}}^{\mathrm{F}} = .8059$ (see Supplementary Table 22). Removing female anti-contraceptive drugs only slighly lowers *RRI*^*F*^ from 1.59 to 1.55.

**Fig. 1:**
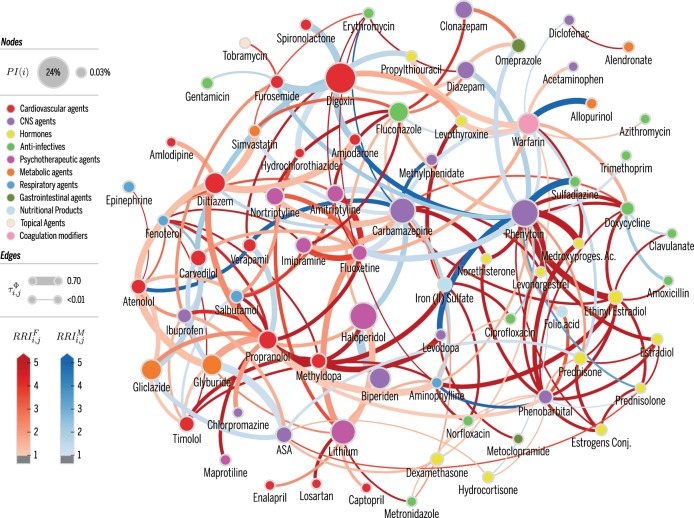
DDI network. A weighted version of network Δ where weights are defined by $\tau _{i,j}^{\mathrm{\Phi }}$. Nodes denote drugs *i* involved in at least one co-administration known to be a DDI. Node color represents the highest level of primary action class, as retrieved from Drugs.com (see legend). Node size represents the probability of interaction *PI(i*), as defined in text. Edge weights are the values of $\tau _{i,j}^{\mathrm{\Phi }}$ obtained from Eq. (4). Edge colors denote $RRI_{i,j}^g$, where *g* ∈ {M, F}, to identify DDI edges that are higher risk for females (blue) or males (red). Color intensity for $RRI_{i,j}^g$ varies in [1,5]; that is, values are clipped at 5

To understand the DDI phenomenon at large as well as which drugs are most responsible for the higher risk of DDI women face over men, we also computed *DDI networks* that characterize drug pairs according to measures of patient volume $(|U_{i,j}^{\mathrm{\Phi }}|)$ and DDI association strength $(\tau _{i,j}^{\mathrm{\Phi }})$. One of these networks is shown in Fig. 1 (others shown in Supplementary Note 6). The 75 drug nodes involved in DDI are colored by their primary action class. Node size represents the probability of interaction (*PI*) of a drug, *PI*(*i*), with larger nodes identifying drugs most contributing to potential ADR from DDI. To better grasp gender differences in the DDI phenomenon, edges are colored according to the *relative risk of drug pair interaction for each gender*, $RRI_{i,j}^g$ with *g* ∈ {F, M}, such that red (blue) edges denote increased DDI risk for women (men).

Of the |Δ| = 181 DDI edges, 133 are associated with an increased risk for women, whereas only 48 denote an increased risk for men—a ratio of 2.8. Removing hormone therapy drugs from the network changes the number of edges associated with increased risk for women from 133/181 = 73.48% to 116/158 = 73.42%; for men the ratio changes from 48/181 = 26.52% to 42/158 = 26.58%. In other words, there is virtually no change when hormone therapy drugs are removed from the network. Looking at the subgraph comprised only of very gender-imbalanced pairs, ${RRI_{i,j}^{g}}\,>\,{3}$, we find 49 drugs in interactions that affected 3,327 women (4.28% of female Pronto population), but only 13 drugs in interactions that affected 64 men (0.01% of male Pronto population). The 65 (9) such interactions for women (men) contain 16 (3) that are considered major (see also Supplementary Figs. 18 and 19). Table 3 shows the top major DDI pairs per gender which affected at least 10 patients; interestingly, only two DDI pairs that affect at least 10 patients were observed with a higher relative risk of interaction for males—see Supplementary Tables 18 and 19 for full listings.

**Table 3:** Top 10 known major DDI pairs

$|U_{i,j}^{{\mathrm{\Phi }},F}|$	*i*	*j*	$RRI_{i,j}^F$	$|U_{i,j}^{{\mathrm{\Phi }},M}|$	*i*	*j*	$RRI_{i,j}^M$
13	Carbamazepine	Ethinyl Estradiol	∞	29	Digoxin	Amiodarone	1.78
13	Levonorgestrel	Carbamazepine	∞	11	Diclofenac	Warfarin	1.19
1,411	ASA	Ibuprofen	1.42	–			
992	Amitriptyline	Fluoxetine	3.55	–			
703	Fluconazole	Simvastatin	2.63	–			
209	Imipramine	Fluoxetine	3.08	–			
302	Diltiazem	Simvastatin	1.27	–			
159	Fluoxetine	Lithium	1.79	–			
122	Fluoxetine	Nortriptyline	2.70	–			
28	Propranolol	Salbutamol	6.61	–			

Top 10 known *major* DDI pairs (*i*, *j*) with increased risk of co-administration per gender, *g* ∈ {M, F}, which affected at least 10 patients of each gender. Rows ordered by the rank product of the ranks of $RRI_{i,j}^g$, the relative gender risk of co-administration, and $|U_{i,j}^{{\mathrm{\Phi }},g}|$, the number of patients of given gender affected by the DDI

### Age risk

**Fig. 2:**
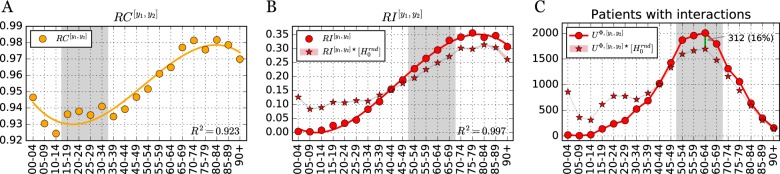
Risk of co-administration and interaction per age range. **a, b** Co-administration ($RC^{[y_1,y_2]}$) and interaction risk ($RI^{[y_1,y_2]}$) per age group, computed via Eq. (8). Solid orange line is the cubic regression for $RC^{[y_1,y_2]}$ while solid red line is the cubic regression for $RI^{[y_1,y_2]}$ (linear and quadratic regressions in Supplementary Information). **c** Absolute number of patients with at least one co-administration known to be a DDI. For all plots, age groups [90,94], [95,99], were aggregated into [90+]. Stars $( \star )$ depict values computed from the null model, $H_0^{rnd}$, with background filling denoting the 95% confidence interval based on 100 runs

To investigate the role of age in DDI co-administration we calculated two additional measures, the risk of co-administration (*RC*) for age group, $RC^{[y_1,y_2]}$, and the risk of interaction (*RI*) for age group, $RI^{[y_1,y_2]}$. If the number of DDI observed were proportional to the number of co-administrations, the latter quantity would be essentially flat across age groups (see Eq. 8 in Methods). As shown in Fig. 2, center, *RI* increases substantially for older age groups (see also Supplementary Table 23), varying from near zero for younger age groups to 0.35 for groups over 70. While there is some variation, *RC* varies a lot less than *RI*—no more than 6% across all age groups as seen Fig. 2-left (note the difference in scale). This shows that risk of co-administration is largely proportional to the number of dispensed drugs, while risk of interaction seems to grow more than the increase in co-administrations (polypharmacy) observed with age.

The risk of co-administration is overall quite high for all age groups ($RC^{[y_1,y_2]} \in [.92,.98]$), with increasing values as patients age. Patients dispensed at least two drugs are almost always being dispensed drugs concomitantly. Conversely, the risk of interaction starts from almost nonexistent at age [0–14] and reaches more than 25% after the age of 55.

**Fig. 3:**
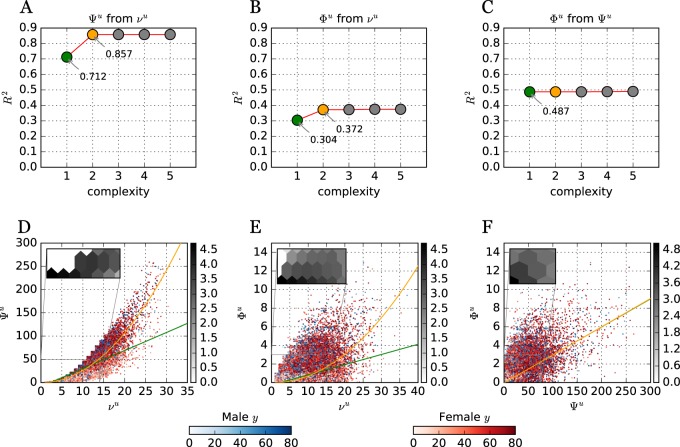
Patients with their number of drugs dispensed *ν*^*u*^, co-administrations Ψ^*u*^ and interactions Φ^***u***^. **d–f** Each circle depicts a patient, with red (blue) circles denoting females (males). Color intensity denotes their age, with stronger red (blue) representing older women (men). To reduce circle overlay and enhance visualization, a uniform noise ∈[0,1] was added to both coordinates. Green and orange lines denotes linear and quadratic regressions, respectively. Inserts with Hexagonal log-bins are included to better depict the density of patients close to the origin. **a–c** Pareto fronts comparing regression results (*R*^2^) at increasing regression model complexity. For example, complexity 1 and 2 denote a linear and quadratic regression, respectively

The relationship among the number of drugs dispensed (*ν*^*u*^), co-administrations (Ψ^*u*^), and interactions (Φ^*u*^) for all users is shown in Fig. 3. While there is a strong nonlinear (quadratic) relationship between *ν*^*u*^ and Ψ^*u*^ (Fig. 3-**d**), there is no evidence of a nonlinear relationship between Ψ^*u*^ and Φ^*u*^ (Fig. 3-**f**), which could explain the observed growth of RI with age—which implies that interactions grow faster than co-administrations with age. In contrast to previous reports,^20^ co-administrations (Ψ^*u*^) predict interactions (Φ^*u*^) better than number of drugs prescribed (*ν*^*u*^), though neither do so particularly well.

**Fig. 4:**
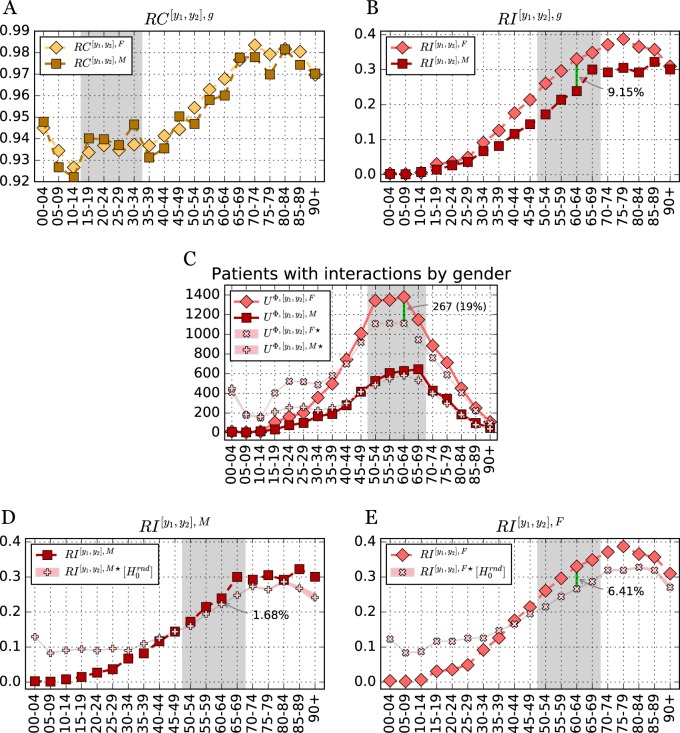
Risk of co-administration and interaction per age range and gender. **a** Risk of co-administration per age group and gender, $RC^{[y_{1},y_{2}],g}$. **b** Risk of interaction per age group and gender, $RI^{[y_{1},y_{2}],g}$. **c** Absolute number of patients with at least one known DDI co-administration, per age and gender $U^{{\Phi},[y_{1},y_{2}],g}$. **d, e** Female and male risk of interaction per age group and gender, $RI^{[y_{1},y_{2}],F}$ (**d**) and $RI^{[y_{1},y_{2}],M}$ (**e**). For all plots, age groups, [90,94], [95,99], [90,90+] were aggregated into [90+]. Stars ($\star$) depict values computed from the null model, $H_{0}^{rnd}$, with background filling denoting the 95% confidence interval based on 100 runs. Shaded areas identify specific age groups mentioned in the main manuscript

To further investigate whether factors other than increase in co-administration cause the increase of DDI risk with age, we developed a statistical null model; values reported for the null model are identified with a star ($\star$) and associated 95% confidence intervals (for 100 runs) in Fig. 2. The idea is to explore if the growth of *RI*^*y*^ is an expected phenomenon of increased polypharmacy with age, which necessarily results in a combinatorial increase of possible drug pairs that can interact. The null model was not able to reproduce the observed behavior of *RI*^*y*^ (*X*^2^ = 2840.6, *p* < .01), especially for older and younger ages (see Figs. 2 and 4 and Supplementary Information 7 for additional details).

We observe that for younger ages, *RI*^[0,29]^ is much lower than the model’s predicted $RI^{[0,29] \star }$ (Fig. 2-**b**); the same is true for the number of patients affected (Fig. 2-**c**). The largest discrepancies between model and real data occur at this age range, especially [0,4] and [20,24]. However, this expected behavior is inverted for ages [50+], with the transition occurring around age [40,44] (Fig. 2-**b**). For older ages, the largest discrepancies between model and reality occur for age groups in [50,70], where the predicted number of patients with DDI $\left( {|U^{{\mathrm{\Phi }} \star }|} \right)$ for age group [60,64] is 16% lower than what is observed (see Fig. 2-c).

We additionally parse age risk by gender by computing $RC^{[y_1,y_2],g}$ and $RI^{[y_1,y_2],g}$, shown in Fig. 4 (see also Supplementary Tables 24 and 25). Both genders have overall similar risk of co-administration in all age groups. Even during childbearing age, the co-administration risk is similar for the numbers of drugs dispensed, even if slightly larger for females (see filling in Fig. 4-**a**). Interestingly, for $RI^{[y_1,y_2],g}$ a clear difference between genders occurs after childbearing age, maximized between 50 and 69 years old (see filling in Fig. 4-**b** and absolute number of patients in Fig. 4-**c**). The gender difference in *RI* appears after the age of 35, reaching more than a 9% difference for age group [60,64].

Figure 4-**d–e** show the null model’s gender risk of interaction $RI^{[y_1,y_2],g \star }$, in comparison to observed values, $RI^{[y_1,y_2],g}$, for men (**d**) and women (**e**), respectively. For both genders, we still observe that the real *RI* for children and young adults ([0,34]) is well below the null model. However, the transition observed for older age is much more pronounced for women. In fact, after age 40, observed male *RI* is largely consistent with the null model, while female risk is higher.

### Prediction of patients with DDI

We computed several multiple regression (MR) models. These show that the inclusion of additional variables does not improve much at all the prediction of the variance of Φ^*u*^. For instance, a MR with both *ν*^*u*^ and Ψ^*u*^ leads only to very marginal increase in the explained variance of Φ^*u*^: adjusted *R*^2^ = 0.492. Adding higher order, nonlinear models also does not improve upon the original regression between Ψ^*u*^ and Φ^*u*^. Even the inclusion of demographic variables in MR models does not lead to improvement of *R*^2^ for Φ^*u*^—we analyzed many neighborhood-level variables such as average income, robbery, theft, suicide, transit crime, trafficking, and rape rates. Restricting the analysis to the subset of patients who reported education, and using it as an independent categorical variable also yields no improvement (see Supplementary Information 102 for MR and ANOVA details).

Interestingly, even the inclusion of gender as a categorical variable, does not improve *R*^2^ for Φ^*u*^. At first glance, this seems a somewhat counter intuitive result, given the observed high risk of DDI for females in comparison to males. However, the MR analysis revealed that even though women certainly face a much greater risk of DDI, the number of DDI pairs they are administered (Φ^*u*^) is on average similar to that of men, and both have large variance of Φ^*u*^ (see Supplementary Fig. 5). Thus, while gender clearly is a very strong factor in the risk of at least one DDI, it is not a good predictor of the specific number of interactions per patient.

Therefore, we sought to answer the question of how well we can automatically predict patients with at least one DDI (not the number of interactions per patient)? Using binary classifiers we are able to achieve very good performance on this task. Classifiers perform well above null models, with MCC ≈0.7 and excellent AUC scores: AUC ROC ≈0.97 and AUC P/R ≈0.83.

## Discussion

Our 18-month longitudinal analysis of EHR data of the entire city of Blumenau allowed us to study the DDI problem in primary and secondary care in greater detail and for a longer period of time than what has been hitherto possible. In summary, the DDI phenomenon is stable across the city, and proportional to population size—demonstrating no major inequalities due to income, education, crime, or other neighborhood social factors, which suggests a balanced and fair access to medical care in Blumenau. Our analysis revealed that ≈12% of all patients of the *Pronto* HIS where administered known DDI, which represents 5% of the entire Blumenau population. If we consider only the adult population, ≈15% were dispensed a known DDI (more than 6% of the Blumenau adult population). Looking at the type of DDI, we observe that 4% of all patients (5% of adults) were dispensed a major DDI likely to result in a very serious ADR—almost 2% of the city’s population.

Given the lack of similar studies, we cannot directly compare the rate of DDI severity observed in Blumenau to other public health systems. The Tayside study (with a smaller, 84 day observation window) reported a rate of 13% “potentially serious” DDI for adult patients.^20^ This severity class was derived from the *British National Formulary*, a private publication we do not have access to. If this severity is similar to the *Drugs.com* major DDI class, then Blumenau has a considerably lower rate of this type of DDI than Tayside—5–13%. If, on the other hand, “potentially serious” encompasses both the major and moderate *Drugs.com* DDI classes, then the rates observed in Blumenau are similar to those observed in Tayside—14.35% to 13%.

We uncovered 181 DDI pairs that most likely could have been prevented.^12^ These drugs known to interact were nonetheless dispensed for co-administration to 15,527 people, including more than five thousand who were administered a major DDI, likely to require medical attention. In addition to the human suffering caused, patient hospitalization due to major DDI may lead to a large financial burden to health-care systems. All our estimates lead to very substantial costs for the various levels of government, suggesting that the financial burden of DDI is at least double what was previously reported—$1 per capita in Ontario^9^—even when considering the most conservative estimate of the proportion of hospitalizations that derive from co-administration of known major DDIs. Thus, our large-scale longitudinal analysis suggests that previous estimates based on smaller studies likely underestimate the cost of the DDI phenomenon.

We provide comprehensive lists of the DDI pairs uncovered in the data, allowing others to look at specific drugs of interest. The data can be seen from different angles, such as the volume of people affected or the likelihood that certain drugs are co-administered. These include common medications such as proton-pump inhibitors (Omeprazole), anti-depressants (Fluoxetine), or common analgesics (Ibuprofen), as well as not so common drugs (e.g., Erythromycin). It is noteworthy that the DDI co-administration of CYP(3A4 and 2D6) inhibitors with their respective enzymes substrates was often found in our results. From our dataset CYP[3A4] inhibitors include Omeprazole, Fluconazole and Erythromycin and their respective substrates include Clonazepam, Simvastatin, and Carbamazepine. Recently, the FDA included a comparison list^37^ of in vitro and clinical inhibitors, inducers and substrates for CYP-mediated metabolisms. In agreement with previous work,^16^ our analysis revealed several such DDI, including the most common DDI pair in our data (Omeprazole, Clonazepam). Many other major interactions, while not ranked at the top, are nonetheless of concern due to severe ADR. For instance, in 2011 the FDA issued a warning^38^ contraindicating the concomitant use of Simvastatin with Erythromycin, due to increased risk of myopathy by “possibly increasing the statin toxicity”. Still, our analysis identified 10 patients concomitantly administrating this major DDI (117th, $|U_{i,j}^{\mathrm{\Phi }}| = 10$), also known for its increased risk of liver damage and a rare but serious condition of rhabdomyolysis that involves the breakdown of skeletal muscle tissue.^28,39^

Our network representation also allows us to integrate, summarize and visualize the DDI phenomenon. The analysis of the network itself also reveals nodes with largest degree, that is, drugs that participate in more known DDI. The top ones, participating in over 10 distinct DDI are: Phenytoin, Carbamazepine, Phenobarbital, Propranolol, Warfarin, Aminophylline, Fluoxetine, Fluconazole (see Supplementary Table 27 for others). Drugs in italic have both high degree and high *PI*, meaning they interact with many other drugs and are also more likely to interact with some other drug when dispensed. The network also allows us to investigate the roles of individual drugs and DDI pairs, in relation to others. For instance, Phenytoin, an anti-seizure medication, is the drug with largest degree and node size: it interacts with 24 other drugs, granting it the highest total degree strength, $\mathop {\sum}\nolimits_j {\tau _{ij}^{\mathrm{\Phi }}} = 6.51$; one in five times that Phenytoin is co-administered with another drug it leads to an interaction, *PI*(*Phenytoin*) = 0.2; and it also has the largest betweeness centrality (0.30),^40^ thus acting as bridge between other drugs with known DDI.

Our characterization of the significant demographic factors in the DDI phenomenon, shows that women in Blumenau are at a strikingly greater risk of being dispensed known DDI than men, with a 1.6 risk multiplier. In other words, women in the Blumenau’s *Pronto* system have an almost 60% increased risk over men of being dispensed a DDI, but only a 6.5% increased risk of being dispensed drugs concomitantly. When only major DDI are considered the risk multiplier is even higher: 1.9. That is, women have almost double the risk of men of being dispensed a major known DDI. It is noteworthy that we pursued a relative risk analysis for all age groups, showing that females face a greater or similar risk of DDI than males in all age groups, with substantially higher risk observed after 50 years of age. For instance, in age group [60−64], one in three women who are dispensed two or more drugs concomitantly face a known DDI, whereas that ratio is less than one in four for men for the same age group (see Fig. 4). Therefore increased risk for females is not confounded by the larger number of women present in the data nor their age.

It is known that age is also a factor in predicting the number of prescribed drugs,^36^ especially because of increased comorbidity in older patients. Our analysis shows that one in every four patients over 55 is likely to be face a known DDI when co-dispensed two or more drugs. The risk of interaction for older age groups of both genders is also severe, reaching more than 30% for adults over 70 years of age in comparison to younger age groups. While a greater risk for older age groups is expected due to increased polypharmacy with age, a comparison of the observed risk with a null model accounting for random polypharmacy (and preserving same number of co-administrations per age) shows that it does not explain the high levels of interactions older age groups face. This can be contrasted with the almost nonexistent number and risk of interactions in children, which are considerably lower than what the null model predicts for polypharmacy at that age. It is very surprising, indeed shocking, that there are more cases (and increased risk) of DDI in older age than random (age-conditioned) dispensation of drugs would yield. We would expect all age groups to have fewer cases than a random null model, but this is only observed for younger age groups.

The null model also revealed an additional gender bias, as older women clearly have a worse-than-random, while older men have a more similar-to-random risk of DDI in most age groups. In fact, deviation from the null model in older age is mostly explained by increased risk for females. In contrast, younger age groups of both genders have much better-than-random risk of DDI.

These observed gender and age risks suggest two possible hypothesis: specific drugs dispensed to women or older populations are more dangerous; and/or that not as much attention to DDI in primary care is reserved for these populations. The fact that the specific drugs dispensed greatly improve the automatic prediction of patients with DDI favors the first hypothesis, but given the age and gender risks observed, it is also clear that the same DDI-prone drugs are administered differently between genders and across age groups. This second hypothesis is strengthened by the fact that removing female-specific hormone therapy from the the DDI network of Fig. 1 barely reduces the DDI gender risk (from 59 to 55%). Indeed, the DDI pairs with increased risk for women traverse all drug classes and are not gender-specific, ranging from cardiovascular to central nervous systems agents.

While it was already known that drugs withdrawn from the market for ADR presented greater risks for women,^41^ our study demonstrates that women (and older populations) in Blumenau also face a higher risk of being dispensed known DDI. It could be that in older age groups (especially for women) there are fewer alternative drugs (with fewer adverse reactions) in the Blumenau public system, either because they are more expensive or simply because they are not available anywhere, thus forcing the prescription of known DDI. These and other possibilities warrant further study outside the scope of the present article. For instance, would the introduction of newer and costlier drugs into the public system, overcome the financial and human burden of current DDI levels? Nonetheless, since medical care should in principle provide a better-than-random risk of DDI for all age groups and genders, our results suggest that factors of a social, biological, or medical-care nature are at play at the primary- and secondary-care levels and should be further studied everywhere.

The performance achieved by our classifiers demonstrates that a useful computational intelligence pipeline can be devised to flag patients for further assessment by a primary-care physician, pharmacist, public official, or even to request a home visit from a community health agent. Existing prescription alert systems already warn against known DDI, still, these are evidently being prescribed in worrying numbers. This could be because there are good medical reasons to prescribe certain drug combinations despite known DDI risk, or because drugs may be prescribed by distinct physicians, who may not be aware of or check previous prescriptions, or simply dismiss HIS alerts^42^—perhaps due to physician alert fatigue.^43^

To be useful, personalized alert systems based on the type of predictions produced by our classifiers do not necessarily need to be added to prescription systems. Indeed, their utility lies not in identifying known DDI pairs—as those are already by definition available via formularies, web resources like Drugbank, or prescription HIS—but rather in identifying patients at greater risk of being prescribed DDI in the future, or subpopulations and comorbidities that for social or biomedical reasons face greater risk of DDI. Thus, they should be more useful for those involved in integrating and managing the care of individual patients or the entire public-health system. Those are decisions that each public-health system will have to weight. Still, our work demonstrates that a personalized alert system for DDI is accurate and can be used to reduce the DDI phenomenon not only in future versions of the *Pronto* HIS, but in other cities that have observed high levels of DDI—e.g., the Tayside region, in Scotland.^20^ In future work we intend to add such a pipeline to *Pronto* as well as utilize new data sources such as social media, since *Pronto* already includes such patient handles. Indeed, such data may allow early-warning signal detection of adverse events and DDI.^44,45^

Large-scale analyses of EHR to establish the prevalence of known DDI are rare. Most studies are obtained from small populations in hospital settings, so they vary by a large margin.^6,11,12,46^ Our study of the entire city of Blumenau at the primary- and secondary-care level offers an important new large-scale measurement of the DDI phenomenon in a public health-care system—a baseline that can be compared to other worldwide locations beyond Brazil, as EHR data become available. For instance, are the gender and age risk levels we observed similar in other primary- and secondary-care settings? Are there cultural or public/private differences? Will the health systems of other cities also prove to be unaffected by neighborhood and income levels, etc?

Our large-scale epidemiological analysis demonstrates that an integrated data- and network-science approach to public health can uncover biases in the DDI phenomenon as well as yield tools capable of issuing accurate DDI prediction per patient. Both outcomes contribute to preventing ADR from DDI and thus may lead to a significant positive impact on the quality of life of patients and finances of public-health systems. Moreover, the gender and age risks of DDI we discovered, should inform physicians and other health professionals anywhere that such factors are important in the drug management of their patients. We expect the results to increase awareness of those risks we uncovered.

## Methods

### Data

Eighteen months of drug dispensing data (Jan 2014–Jun 2015) were gathered from the *Pronto* HIS^47,48^ (see Supplemental Note 1 for a system description). Drugs reported in this system are available via medical prescription only, free of charge, and dispensed to citizens of Blumenau (population Ω = 338,876^49^) during the observation period. Doctors prescribe medications by selecting drug and dosage via the HIS. Low-cost drugs can generally be directly dispensed at the primary-care facilities, whereas specialized and higher-cost medication is distributed in three central facilities across the city. All drugs are dispensed by pharmacists who must select in *Pronto* the drug and quantity to be dispensed, allowing the length of administration to be estimated. It must be noted that patients are not required to retrieve drugs from the public system. They can buy prescribed medications from private pharmacies at their own expense, without such transactions being recorded in *Pronto*. However, there is no incentive to pay more at private pharmacies for the same medication. Indeed, our analysis indicates that use of *Pronto* is similar across all neighborhoods of Blumenau, irrespective of their average income (see Supplementary Fig. 3).

EHR were anonymized at the source and only drug dispensation and demographic variables, including gender, age, neighborhood, marital status, and educational level, were kept. Methods were performed in accordance with guidelines and regulations. All patient consent was handled at the source prior to the anonymization and outside of the responsibility of this team. Nonetheless, this study was approved by Indiana University’s Institutional Review Board (IRB). Drug names originally in Portuguese were converted to English, disambiguated and matched to their DrugBank ID (e.g., Cefalexina 500 mg Comprimido and Cefalexina 250MG/5 ml Suspenso Oral were matched to Chlorphenamine, DBID DB01114). Medications with multiple drug compounds (e.g., Amoxicillin 500 mg & Clavulanate 125 mg) were split into their constituent individual drugs. Other dispensed substances (e.g., infant formula milk or vitamin complexes) unmatched to DrugBank were discarded. In total, 122 unique drugs were keep for analysis. Because we have no means to know whether patients actually took the dispensed drugs, our analysis assumes that drugs dispensed were administered.

**Fig. 5:**
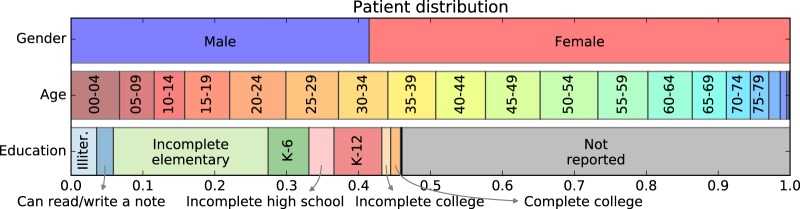
Distribution of patients given gender, age and education level. In total |*U*^M^| = 55,032 (41.46%) were males and |*U*^F^| = 77,690 (58.54%) were females. On education, a majority |*U*^*e* = ∅^| = 71,662 (53.99%) did not report their education level. |*U*^*e*−^| = 48,547 (36,58%) declared having at most some high school education whereas |*U*^*e* +^ | = 12,513 (9,43%) had completed high school education or above. On age, patients |*U*^*y* = [20,24]^| = 10,382 (7,82%) and |*U*^*y* = [50,54]^| = 10,650 (8,02%) accounted for the two largest age groups. Labels K-6 and K-12 are Completed elementary and Completed high school education, respectively. Labels for age *y* ≥ 80 and education level above Completed college not shown

Throughout the year of 2014 and the first six months of 2015, Blumenau’s *Pronto* HIS registered 1,573,678 distinct drug interval administrations, dispensed to |*U*| = 132,722 distinct patients—39.17% of the city population. The male/female proportions are 41.5/58.5%, respectively. Of the 46% who declared their education level, a large proportion (46.77%) reported having incomplete elementary school and 20.49% had finished high school or above (see Fig. 5 and Supplementary Fig. 5 for details). |*U*^*ν*≥2^| = 104,811 patients, corresponding to 78.97% of the *Pronto* patient population, were dispensed two or more distinct drugs in the period; only this set could have been dispensed known DDI.

### Methods

**Fig. 6:**
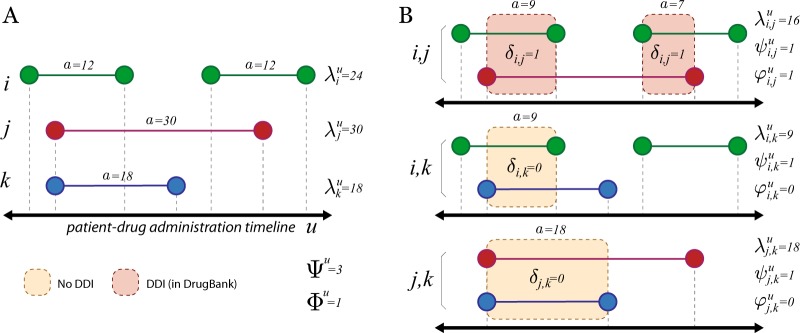
Diagram of co-administration and interaction computation. **a** A hypothetical patient-drug dispensing timeline with three drugs (*i*, *j*, and *k*). Drug administration length (*a*, in days, *n*) are shown for each dispensation. **b** The three possible pairwise comparisons (*i*, *j*), (*i*, *k*), and (*j*, *k*) between the dispensed drugs are shown with their co-administration overlap marked with either an orange (no known DDI) or red (known DDI) background

A drug interaction between a pair of drugs is measured if both drugs were concomitantly administered and the pair is identified as a known DDI in the 2011 version of DrugBank, an open-source drug database containing DDI information.^50^ Figure 6 displays a co-administration timeline example. More formally (see also Table 4 and Supplemental Note 3), let us denote patients by *u* ∈ *U* and drugs by *i*, *j* ∈ *D* (|*D*| = 122); *U*_*i*_ ∈ *U* is the subset of users who were dispensed drug *i*, *D*^*u*^ ⊆ *D* is the subset of drugs administered to patient *u*, and *ν*^*u*^ ≡ |*D*^*u*^| is the number of distinct drugs dispensed to patient *u*. Patients can be administered a drug *i* multiple times in the observation period, therefore $A_i^u \equiv \{ a_n^{i,u}\}$ denotes the set of distinct administration intervals *a* of drug *i* to patient *u*, where $a \in {\Bbb N}$ is measured in days (*n*). $\alpha _i^u = |A_i^u|$ and $\lambda _i^u = \mathop {\sum}\nolimits_n {a_n^{i,u}}$ denote the number of times and total number of days drug *i* was administered to patient *u*, respectively.

**Table 4:** Co-administration and interaction quantities and subsets used throughout the analysis

Quantity notation	Number of
*ν*^*u*^ ≡ |*D*^*u*^|	Distinct drugs dispensed to patient *u*.
${\mathrm{\Psi }}^u = \mathop {\sum}\limits_{i,j \in D^u} {\psi _{i,j}^u}$	Co-administrations to patient *u*.
${\mathrm{\Psi }}_{i,j} = \mathop {\sum}\limits_{u \in U} {\psi _{i,j}^u}$	Co-administrations of drug pair (*i*, *j*) to all patients.
${\mathrm{\Phi }}^{u} = \mathop {\sum}\limits_{i,j \in D^{u}} {\varphi _{i,j}^{u}}$	Co-administrations of known DDI pairs to patient *u*.
${\mathrm{\Phi }}_{i,j} = \mathop {\sum}\limits_{u \in U}{\varphi _{i,j}^{u}}$	Co-administrations of known DDI pair (*i*, *j*) to all patients.

Subset notation	Subset of patients
*U*^*ν* > *x*^ = {*u* ∈ *U*:*ν*^*u*^ > *x*}	Who had at least $x \in {\Bbb N}$ drug administrations.
*U*^Ψ^ = {*u* ∈ *U*:Ψ^*u*^ > 0}	Who had at least one co-administration.
$U_{i,j}^{\mathrm{\Psi }} = \{ u \in U:\psi _{i,j}^u = 1\}$	Who were co-administered drug pair (*i*, *j*).
*U*^Φ^ = {*u* ∈ *U*:Φ^*u*^ > 0}	Who had at least 1 known DDI.
$U_{i,j}^{\mathrm{\Phi }} = \{ u \in U:\varphi _{i,j}^u = 1\}$	Who were co-administered known DDI pair (*i*, *j*).
*U*^*g*^ = {*u*∈*U*:*gender*(*u*) = *g*},*g*∈{M, F}	Per gender.
$U^{[y_1,y_2]} = \{ u \in U:age(u) \in [y_1,y_2]\} ,y_1,y_2 \in {\Bbb N}$	Per age bracket.
$U^N = \{ u \in U:neighborhood(u) \in N\} ,N \in {\Bbb N}$	Per neighborhood.
$U^E = \{ u \in U:education(u) \ge E\} ,E \in {\Bbb N}$	Per education level. *U*^*E* = ∅^ is the subset of patients who did not report their education level.

From these subsets we also denote their possible intersections by combining the appropriate sub and superscripts

Similarly, $\alpha _{i,j}^u$ and $\lambda _{i,j}^u$ denote the number of times and total number of days (co-administration length) drugs *i* and *j* were co-administered to patient *u*, respectively (see Supplementary Note 2 for more details of co-administration measurement). To identify the co-administration of drug pair (*i*, *j*) to patient *u* we define a Boolean variable $\psi _{i,j}^u \in \{ 0,1\}$ as:

1 $$\psi _{i,j}^u = (\lambda _{i,j}^u > 0)$$

a logical variable measuring whether patient *u* co-administered drug pair (*i*, *j*) for at least one day. Next, we define a symmetrical binary map Δ:*D* × *D* → {0, 1} to indicate whether drug pair (*i*, *j*) ∈ *D* × *D* is (*δ*_*i*,*j*_ = 1) a known DDI in DrugBank, or not (*δ*_*i*,*j*_ = 0). Thus, to flag the co-administration of a known drug interaction (*i*, *j*) to patient *u* we similarly define a Boolean variable $\varphi _{i,j}^u \in \{ 0,1\}$ as:

2 $$\varphi _{i,j}^u = (\psi _{i,j}^u = 1 \wedge \delta _{i,j} = 1).$$

For each DDI pair observed, literature references and a severity score *s*∈{major, moderate, minor, n/a} were retrieved from Drugs.com.^28^ From these values, other quantities and sets are computed per patient *u*, drug *i* or drug pair (*i*, *j*) as listed in Table 4.

The drug pairs (*i*, *j*) with the largest “footprint” in the population, are the pairs that maximize $|U_{i,j}^{\mathrm{\Psi }}|$. Out of these most co-administered pairs, we are naturally most interested in those that are known DDI and thus maximize $|U_{i,j}^{\mathrm{\Phi }}|$. A normalized version of this measure is computed as

3 $$\gamma _{i,j}^{\mathrm{\Phi }} = \frac{{|U_{i,j}^{\mathrm{\Phi }}|}}{{|U_i|}},$$

which conditions the number of users co-administered known DDI pair (*i*, *j*) on the number of users that are administered drug *i*. This measure is not symmetrical: $\gamma _{i,j}^{\mathrm{\Phi }} \ne \gamma _{j,i}^{\mathrm{\Phi }}$. Maximizing it yields DDI pairs (*i*, *j*) that tend to be co-administered to patients who are administered either *i* or *j* independently; see Supplementary Table 12 for top 20 such DDI pairs.

Another facet of the DDI phenomenon we can observe is related to the co-administration length of drug pairs ($\lambda _{i,j}^u$). A normalized version is computed as: $\tau _{i,j}^u = \lambda _{i,j}^u/(\lambda _i^u + \lambda _j^u - \lambda _{i,j}^u)$, where *τ* ∈ [0,1]. This symmetric proximity measure^51^ allows us to distinguish drug pairs that tend to be co-administered to patient *u* only simultaneously $(\tau _{i,j}^u \to 1)$, or with small temporal overlap $(\tau _{i,j}^u \to 0)$. A normalized measure for the entire patient population is then computed as:

4 $$\tau _{i,j}^{\mathrm{\Psi }} = \frac{{\mathop {\sum}\limits_{u \in U_{i,j}^{\mathrm{\Psi }}} {\tau _{i,j}^{u}} }}{{|U_{i,j}^{\mathrm{\Psi }}|}}$$

This proximity measure defines a weighted graph *T*^Ψ^^51^ on set *D*; the graph’s edges, $\tau _{i,j}^{\mathrm{\Psi }} \in [0,1]$, link drugs that were co-administered in the patient population. $\tau _{i,j}^{\mathrm{\Psi }}$ is larger when drug pairs (*i*, *j*) tend to be co-administered when either *i* or *j* is administered (correlated), and smaller otherwise (independent). Therefore, $\tau _{i,j}^{\mathrm{\Psi }}$ is a measure of the strength of drug association in the data for drug pairs (*i*, *j*); high values can pick drug pairs dispensed together for known comorbidities, which physicians should be aware of, as well as for unknown cormobidities (especially involving distinct specialists prescribing drugs independently). Since we do not know the underlying comorbidities, we cannot separate the two cases with this dataset. However, to focus on the DDI phenomenon (for known and unknown comorbidity), we obtain a subgraph *T*^Φ^, restricted to known DDI pairs by computing $\tau _{i,j}^{\mathrm{\Phi }} = \tau _{i,j}^{\mathrm{\Psi }}.\delta _{i,j}$; thus, *T*^Φ^ is a weighted version of Δ.

### Gender risk

The relative risk of co-administration (*RRC*) for women is computed as the ratio of the conditional probabilities of patients being dispensed at least one pair of drugs concomitantly, given gender:

5 $$RRC^{\mathrm{F}} = \frac{{P({\mathrm{\Psi }}^u > 0|u \in U^{\mathrm{F}})}}{{P({\mathrm{\Psi }}^u > 0|u \in U^{\mathrm{M}})}} = \frac{{|U^{{\mathrm{\Psi }},{\mathrm{F}}}|/|U^{\mathrm{F}}|}}{{|U^{{\mathrm{\Psi }},{\mathrm{M}}}|/|U^{\mathrm{M}}|}}$$

Naturally, the same risk for males is computed as *RRC*^M^ = 1/*RRC*^F^. Similarly, we also computed the relative risk of interaction (*RRI*) for women as:

6 $$RRI^{\mathrm{F}} = \frac{{P({\mathrm{\Phi }}^u > 0|u \in U^{\mathrm{F}})}}{{P({\mathrm{\Phi }}^u > 0{\mathrm{|}}u \in U^{\mathrm{M}})}} = \frac{{|U^{{\mathrm{\Phi }},{\mathrm{F}}}|/|U^{\mathrm{F}}|}}{{|U^{{\mathrm{\Phi }},{\mathrm{M}}}|/|U^{\mathrm{M}}|}}$$

with $$RRI^{\mathrm{M}} = 1/RRI^{\mathrm{F}}$$.

### DDI network

The DDI Network is a weighted version of graph Δ where edge weights between drugs *i*, *j* (nodes in graph) are the values $\tau _{i,j}^{\mathrm{\Phi }}$ obtained from Eq. 4—yielding a proximity between drug pairs according to their co-occurrence in DDI co-administrations when either drug is administered (a symmetrical measure of strength of association/correlation^51^). Node size represents the probability of interaction for drug *i*:

7 $$PI(i) = \frac{{\mathop {\sum}\nolimits_j {{\mathrm{\Phi }}_{i,j}} }}{{\mathop {\sum}\nolimits_j {{\mathrm{\Psi }}_{i,j}} }}$$

which denotes the propensity of drug *i* to be involved in a DDI with all drugs it is co-administered with in the data (see Supplementary Table 27 for values); larger nodes thus identify more dangerous drugs in the sense that they most contribute to potential ADR from DDI in our data.

To better grasp gender differences in the DDI phenomenon, edges are colored according to the relative risk of drug pair interaction *for* each gender: $RRI_{i,j}^g$ where *g* ∈ {M, F}. These quantities are computed for each DDI pair (*i*, *j*) via Eq. (6), but using ${\mathrm{\Phi }}_{i,j}^u$ (number of co-administrations of known DDI pair (*i*, *j*) to patient *u*) instead of Φ^*u*^. Naturally, $RRI_{i,j}^F = 1/RRI_{i,j}^M$. If $RRI_{i,j}^F > 1$, the edge is colored in red with intensity proportional to $RRI_{i,j}^F$, otherwise the edge is colored in blue with intensity proportional to $RRI_{i,j}^M$ (see legend). Therefore, increased DDI risk for women (men) is identified by darker red (blue) edges. Supplementary Tables 18 and 19 show the $RRI_{i,j}^g$ values for the top most gender-imbalanced DDI pairs per gender.

For some results we remove the following contraceptive drugs: Ethinyl Estradiol, Estradiol, Norethisterone, Levonorgestrel, and Estrogens Conjugated.

### Age risk

To investigate the role of age in known DDI co-administration, we aggregated patients into age groups and computed the risk of specific age groups to be dispensed a known DDI for the amount of co-administrations observed for that age group. Thus, a risk of interaction for age group [*y*_1_,*y*_2_] is calculated as

8 $$RI^{[y_1,y_2]} = \frac{{P({\mathrm{\Phi }}^u > 0|u \in U^{[y_1,y_2]})}}{{P({\mathrm{\Psi }}^u > 0|u \in U^{[y_1,y_2]})}},$$

which can be interpreted as the probability of being dispensed a known DDI given the expected number of co-administrations for a patient in a specific age range [*y*_1_, *y*_2_]. A risk of co-administration for age group [*y*_1_, *y*_2_], $RC^{[y_1,y_2]}$, is similarly computed, but using *ν*^*u*^ ≥ 2—the number of patients with at least two drug administrations—instead of Ψ^*u*^. This is interpreted as the probability of being concomitantly dispensed two or more drugs (co-administration), when a patient of a given age group is dispensed two or more drugs in the full observation period. Additionally, we also parse age risk by gender by computing $RI^{[y_1,y_2],g}$ for each gender *g* ∈ {*M*, *F*} using Eq. (8), but for users $u \in U^{[y_1,y_2],g}$. Similarly, $RC^{[y_1,y_2],g}$ is computed for the risk of co-administration per age and gender.

### Null model

The null model, $H_0^{rnd}$, aims to capture the expected increase in *RI*^*y*^ with age, given the observed polypharmacy and gender for each specific age group. Thus, the model’s assumption is that all drugs that were in reality dispensed in a given age group are dispensed at random with the same overall frequency of co-administration for that age group. Specifically, for each co-administration observed in the data for an age group [$y_{1}\!,y_{2}$], the null model draws random drug pairs (*i*, *j*) from the set of all drugs observed for that age group, $D^{[y_1,y_2]}$. The random drug pairs are subsequently checked for DDI status in DrugBank, just like the original analysis. This way, the null model has exactly the same number of co-administration occurrences for each age group and gender, but randomly shuffled drug pairs—and only the drugs dispensed for a certain age are randomly shuffled for that age group (additional details in Supplementary Note 7).

### Machine learning classifiers

We trained linear kernel Support Vector Machine (SVM)^52^ and Logistic Regression (LR)^53^ classifiers using stratified 4-fold cross-validation to ensure generalization performance (additional details in Supplementary Note 11). Age, gender, number of drugs (*ν*^*u*^), and co-administrations (Φ^*u*^) were used as demographic variables features. In addition, all |*D*| = 122 drugs in the data are used as binary features, whereby if patient *u* was administered drug *i* that feature is set to 1 and to 0 otherwise; this allows classifiers to be trained on which drugs, and drug combinations, are most likely to be involved in DDI.

The trained classifiers are compared to two “coin-toss” null models, one unbiased where each class has equal probability, and a biased one based on estimated class frequency. A third, more elaborate null model classifier, finds the best age cutoff for each gender, from which all patients above the cutoff age are considered as having a DDI. This last “age-gender” null model represents a baseline comparison of the best we could do if only gender and age were given for each patient. To assess the performance of all classifiers, in Supplementary Note 11 we report several measures. Here, we focus on the Matthew’s Correlation Coefficient (MCC),^54^ which is regarded as an ideal measure of the quality of binary classification in unbalanced scenarios such as this.^55^ We also report two other measures widely used in machine learning classifier performance, the area under the receiver operating charactistic curve (AUC ROC), and the area under the precision and recall curve (AUC P/R).

Other classifiers, feature selection and cross-validation techniques can be used to increase performance, but such gains when studying the DDI phenomenon do not typically lead to substantial performance increases,^56^ so such optimization is beyond the scope of this article.

### Reporting summary

Further information on research design is available in the Nature Research Reporting Summary linked to this article.

## Supplementary information

Supplemental Informatiojn

Reporting Summary Checklist

## Data Availability

The anonymized data that support the findings of this study are available from the city of Blumenau, Brazil. Restrictions apply to the availability of these data, as they may contain information that could compromise research participant privacy through de-anonymization. The data were used under a license agreement, and so are not publicly available, but are, however, available from the authors upon reasonable request and with permission of the city of Blumenau. All data tables and aggregates are available in appropriate electronic form.

## Supplementary information

**Supplementary Information** accompanies the paper on the *npj Digital Medicine* website (10.1038/s41746-019-0141-x).

## Code availability

Custom python and R scripts are available in github.com/rionbr/DDIBlumenau.
