# Supplementary material for: City-wide electronic health records reveal gender and age biases in administration of known drug–drug interactions
Source: NPJ Digit Med. 2019 Jul 23;2:74. doi: 10.1038/s41746-019-0141-x (PMC6650500; doi:10.1038/s41746-019-0141-x)
Supplement: Supplementary file 1 — Supplemental Informatiojn [file 41746_2019_141_MOESM1_ESM.pdf]

Supplemental Information for "City-wide Electronic Health Records Reveal Gender and Age Biases in Administration of Known Drug-Drug Interactions"

Rion Brattig Correia[1,2,3,*], **Luciana P. de Araújo Kohler**[4], **Mauro M. Mattos**[4], **Luis M. Rocha**[1,3,*]

[1]School of Informatics, Computing & Engineering, Indiana University, Bloomington, IN 47408 USA
[2]CAPES Foundation, Ministry of Education of Brazil, Brasília, DF 70040-020, Brazil
[3]Instituto Gulbenkian de Ciência, Oeiras 2780-156, Portugal
[4]Universidade Regional de Blumenau (FURB), Blumenau, SC 89030-903, Brazil
[*] correspondence to rocha@indiana.edu and rionbr@gmail.com

---

1 Pronto: academia, government and patients **2**
1.1 The need for a city-wide HIS 2
1.2 Drug dispensation in Blumenau . . . 3
1.3 Patient education 4

2 Computation details **6**

3 Notation and symbol reference **8**

4 Drug Interactions **10**
4.1 Interactions per severity 17
4.2 Interactions per gender 17
4.3 Interactions per age 18
4.4 Interaction per age and gender . . . 18

5 Risk and Relative Risk measures **19**
5.1 Relative Risk per gender 20
5.2 Relative Risk per severity 24
5.3 Risk Measures per age 24
5.4 Risk Ratios per number of drug . . . 26

6 DDI Networks **26**

7 Null Model for RI^y **35**

8 Interactions per Neighborhood **35**

9 Projected Cost of DDI in hospitalizations **38**

10 Statistical modeling **41**
10.1 Simple Regression (SR) models . . . 42
10.2 Multiple Regression (MR) models . . 45
10.3 Linear Mixed-Effect (LMM) models 50

11 Patient classification **52**
11.1 Simple model 53
11.2 Complete model 53
11.3 No Drugs model 54
11.4 Precision & Recall and Receiver Operating Characteristic curves 55
11.5 Feature loadings 56

Supplementary Note 1 Pronto: academia, government and patients

This section explores some important details about the health information system that made the data presented in this paper possible and tries to enlighten for the broader impact of such projects.

1.1 The need for a city-wide HIS

Apart from systems nationally developed for specific health attention policies—vital statistics, mortality, epidemiology, diabetes, etc—Brazil has no universal electronic health record (EHR) country-wide [1, 2]. Only secondary care (specialists) or high-cost procedures are fed into federal HIS that contains user identification with their national health card (*Cartão Nacional de Saúde*; CNS), even though the majority of services are performed at primary care [1]. The CNS, initiated in 1999 [3], was the initial step towards a unified EHR, but several difficulties were met along these now 20 years of the program [4–6]. Thus, it is currently not possible to follow patients across systems—specially those that only access primary care—or request to the system their medical record. At the city level, most big cities have enough funds to buy specialized, private HIS, to develop and implement EHR along with an intra-city public health development plan for its citizens. On the other hand, the vast majority of small and mid-cities (there are 5,336 cities with less than 100.000 inhabitants [7]) hardly have financial access to the same costly solutions, and most of the information still transits on paper. Still, the necessity to manage several primary care installations, hundreds of health professional agendas and input city-level information into federal HIS takes place.

To address this need in the city of Blumenau, southern Brazil, the municipal government and the regional university (*Universidade Regional de Blumenau*; FURB) joined forces to develop their own open-source HIS to collect and store EHR for its citizens. The system, named *Pronto*, was built by the Laboratory of Technology Development and Transfer (*Laboratório de Desenvolvimento e Transferência de Tecnologia*; LDTT [8]), a small transdisciplinary [9] team of professors and students from diverse fields of research—such as compute science, nursing, medicine, dentistry, psychology, communication, and others—at FURB. This enterprise, bridging academia, government and private sector [10], spun off several scientific quests [8, 11–13] in order to enhance the quality of life of patients in Blumenau, broadening FURB's societal impact, and enabling patients to experience outcomes of scientific research first hand.

After development and deployment, the technology was transferred to the private sector under public bidding regulations, and continues to this day to serve as Blumenau's public health care system under municipal oversight. Pronto is currently used in all health institutions throughout the city. From primary to specialized care—hospitals have their own specialized system—and drug dispensing units. The system currently maintains health professional agendas, integrated medical and dental records, and drug prescription and dispensing across more than 30 health care units.

Doctors prescribe medications by selecting drug and dosage in the electronic system. Low-cost drugs can generally be directly dispensed at the primary-care facilities, whereas specialized and higher-cost medication are distributed in three central facilities across the city. All drugs are dispensed by pharmacists who must select in *Pronto* the drug and quantity to be dispensed, allowing the length of administration to be estimated. There is no pill manipulation as all drugs are dispensed in their original sealed packaging. The database also stores inventory information—in case of drug recall, for instance, patients can be contacted in regards to a specific drug lot.

Pronto runs on a custom built, decentralized database model, where each individual health unit has its own database and a master-to-master replication takes place asynchronously, a design feature due to the unreliable network infrastructure in rural areas. Included in the technical challenges faced by the developing team were also the heterogeneous data feed from multiple health professionals; the transformation from such data data into insightful knowledge to diverse stakeholders, and the constant adaptation to match city as well as state and federal regulations.

A city-wide unified EHR enables a variety of scientific research questions, but most importantly, it permits a holistic approach to public health care. Since patients may enter the system at different clinics throughout the city, the system enables their EHR to be present whenever they go. Moreover, since different health

professionals have access to the EHR, health is not only seen at the individual level but also from the family social structure perspective, a government defined strategy for primary care [14].

Another important system design concept, in line with recent international recommendations for HIS [15], is that all data models are either patient- or family-centered. This means that all health professionals feed data into a model that enables information exchange to better support decisions focused either on the individual or their family. For instance, leaving professional restriction on data privacy aside, let's say patient John D., a young teenager living with his parents initiates a fluoxetine treatment prescribed by a local physician under complains of anxiety. From their conversations on a follow up examination, the doctor decides to ask the patient to check with a psychiatrist. The psychiatrist then detects traces of acute depression. Within the system the specialist has access to the community health agent notes (a type of family health specialist who monthly surveys households around a primary care health center) who, couple weeks back, checked on the family and reported that the household provider lost his/her job. With better social and medical characterization of the problem, all three professionals can now provide a more accurate, personalized, and systematic treatment to the teenager. Furthermore, caring for the family well-being as a whole, and the long-term health of other family members, the physician requests that a social worker be included in the case. This example, albeit simplistic, demonstrates the potential of a holistic approach to public health, focused on prevention, and enabled by a city-wide EHR.

1.2 Drug dispensation in Blumenau

In order to provide readers some geographical context, Supplementary Figure 1 shows the location of Blumenau in Brazil as well as the city neighborhoods with their individual population density.

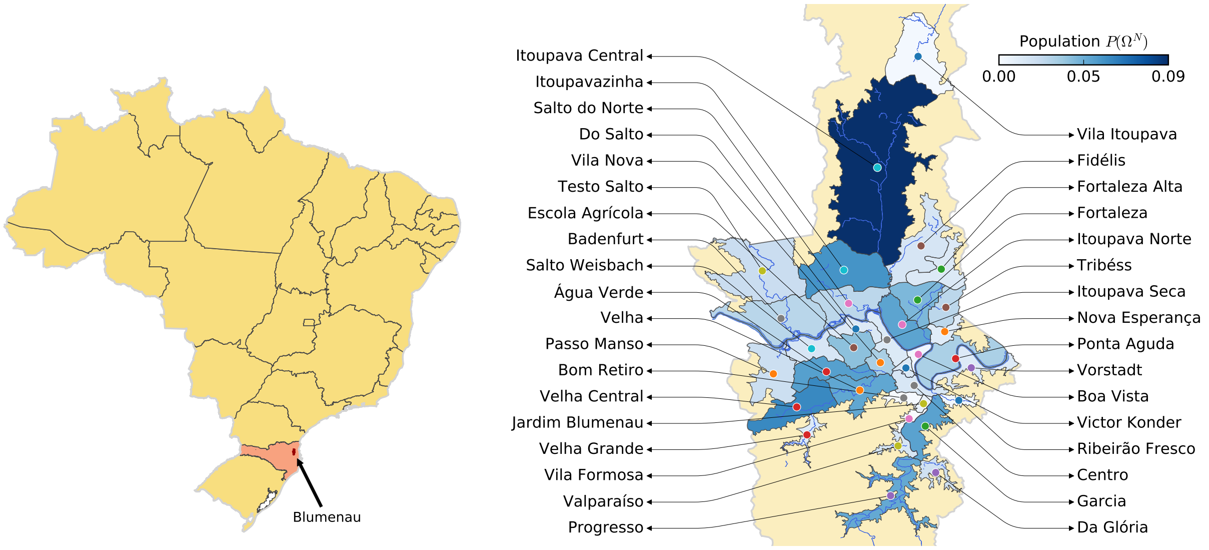

Supplementary Figure 1: (left) Political map of Brazil with state borders. Arrow denotes city of *Blumenau* in the state of *Santa Catarina*. (right) Political map of Blumenau with neighborhoods (N) annotated and mapped to city population, $P(\Omega^N)$. Cartographic shapes from IBGE [7].

The monthly drug dispensation in the city of Blumenau can be seen in Supplemental Figure 2. We conjecture that the smaller number of dispensed medication during summer months (Dec-Feb) are due to a difference portion of the city population taking mandatory 30-days vacations yearly, which are usually split in two 10-days vacations during the summer months, and another 10-days during the winter months. The Atlantic Ocean coast, only a 40 minutes drive east, is a common destination for Blumenau citizens on weekends and holidays. Carnival (*Carnaval*), which is usually held at the end of February, also draws many citizens for a 1 week vacation on the coast.

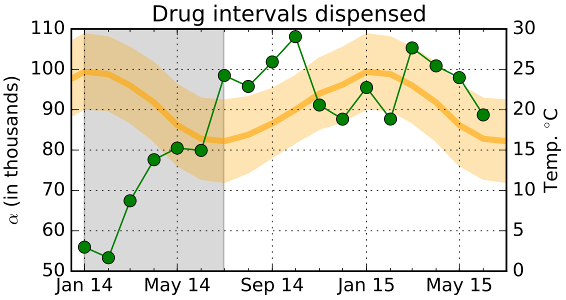

Supplementary Figure 2: Total number (in thousands) of drug intervals dispensed (α) monthly in the city of Blumenau. Orange fill shows average temperature range in Blumenau (in °C). There is no correlation (0.06) between drug dispensation (in non shaded area) and average temperature in the same period. Grey area shows months in which the *Pronto* HIS was under field deployment.

Since Brazil also has private health care and pharmaceutical systems, patients of the public system are often thought to be from lower economical classes, a hypothesis we investigated.

Indeed, the proportion of *Pronto* patients for most age brackets in the four richest neighborhoods—namely *Jardim Blumenau*, *Bom Retiro*, *Victor Konder* and *Vila Formosa*—are significantly smaller than in other neighborhoods(t-test, $p < 3^{-20}$). This strongly suggests that patients from the richest neighborhoods use the public drug dispensation system much less than equivalent groups from other areas (see Supplemental Figure 3).

The only exception to this pattern was found for females age 45-74 from *Bom Retiro* and *Victor Konder* (2nd and 3rd richest neighborhoods, respectively), who, while using the system less than the same group in other neighborhoods, do use it significantly more than those from the richest neighborhood, *Jardim Blumenau* (t-test, $p < 2^{-6}$). This suggests that these two higher-income neighborhoods have a population of older women who uses the public health care system. This may be an interesting phenomenon warranting further sociological studies.

1.3 Patient education

To place the education numbers acquired via *Pronto* system in perspective, we gathered data from the Atlas Brasil Blumenau[1], a United Nations Program for the Development of Brazil (PNUD).

In 2010 the city of Blumenau reported that the proportion of children age 5-6 in school was 88.41%. For the same year the proportion of children age 11-13 attending the last years of elementary school was 90.41%. The proportion of teenagers age 15-17 having completed elementary school was 72,34%. And the proportion of young adults age 18-20 who completed high school was 51.38%. Nationally these number were 91.12%, 84,86%, 57.24% and 41.01%, respectively. The average length of study for children in school age was 10.81 years for Blumenau and 9.97 for the state of Santa Catarina. The number of adults, age 18 or older, who completed elementary school was 65.88% for Blumenau and 54.92% for the state. Considering adults age 25 or older: 2,13% were illiterate, 61,55% completed elementary school, 41,22% completed high school and 15,49%, completed college. Nationally, these proportions are 11,82%, 50,75%, 35,83% and 11,27%, respectively.

Below is the self-reported education distribution for unique patients of *Pronto*. Education level is requested upon registration or profile update and no documents are required. However, staff in health centers are trained to retrieve the best response from patients without their embarrassment—by displaying a card with enumerated answers asking them to respond the according letter.

[1] http://atlasbrasil.org.br/2013/pt/perfil_m/blumenau_sc

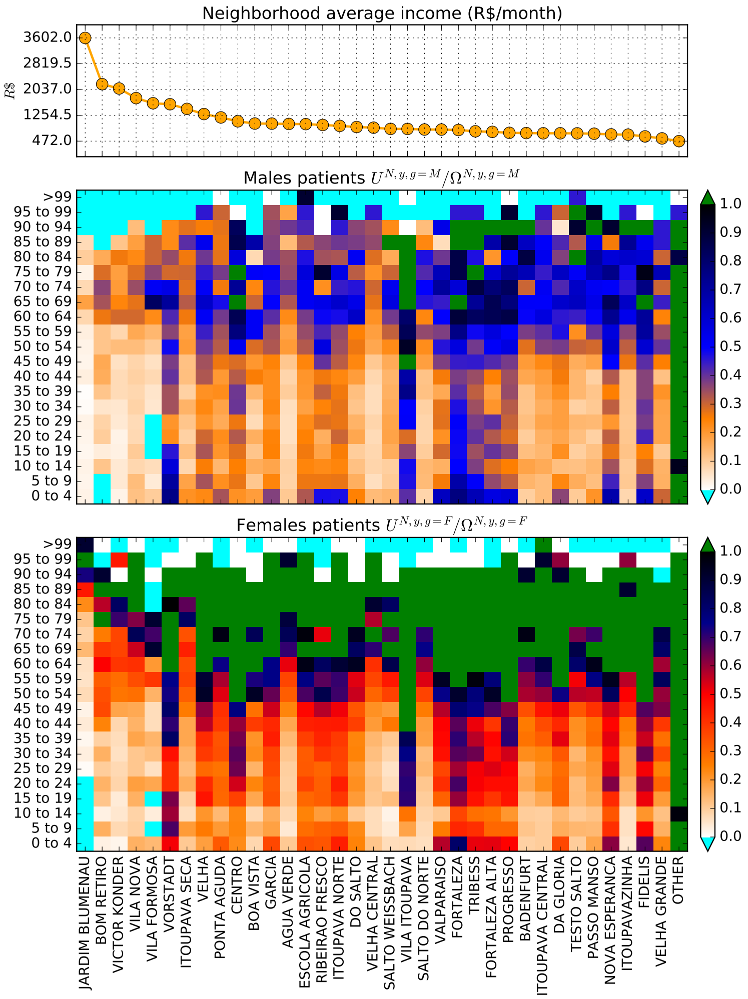

Supplementary Figure 3: Top. Neighborhood average income in Brazilian Reais (R$) [7]. **Middle & bottom.** Age-neighborhood bins of male (middle; $U^{N,y,g=M}/\Omega^{N,y,g=M}$) and female (bottom; $U^{N,y,g=F}/\Omega^{N,y,g=F}$) patients registered in *Pronto* with at least one drug dispensed and matched to DrugBank. Each bin is a probability-like value of patients normalized by official census population data collected and defined by IBGE [7]. Green bins represent values above 1, meaning our data has more patients than IBGE[7] census data. Conversely, cyan bins represent values where our data contains no patient.

E	U^E	%	Ac. %	%	Ac. %
Cant read/write	4,720	0.0356	0.0356	0.0773	0.0773
Can read/write a note	3,104	0.0234	0.0590	0.0508	0.1281
Incomplete elementary	28,557	0.2152	0.2741	0.4677	0.5958
Complete elementary	7,516	0.0566	0.3307	0.1231	0.7189
Incomplete high school	4,650	0.0350	0.3658	0.0762	0.7951
Complete high school	8,797	0.0663	0.4321	0.1441	0.9391
Incomplete college	1,654	0.0125	0.4445	0.0271	0.9662
Complete college	1,823	0.0137	0.4583	0.0299	0.9961
Espec./Residency	192	0.0014	0.4597	0.0031	0.9992
Masters	26	0.0002	0.4599	0.0004	0.9997
Doctoral	21	0.0002	0.4601	0.0003	1.0000
Not reported	71,662	0.5399	1.0000		
Total	132,722	1.0000			

Supplementary Table 1: Education level of *Pronto* patients

E	U^E	%	Ac. %	%	Ac. %
Cant read/write	1,245	0.0134	0.0134	0.0257	0.0257
Can read/write a note	2,552	0.0274	0.0408	0.0528	0.0785
Incomplete elementary	23,983	0.2577	0.2985	0.4957	0.5742
Complete elementary	6,733	0.0723	0.3708	0.1392	0.7134
Incomplete high school	3,126	0.0336	0.4044	0.0646	0.7780
Complete high school	7,544	0.0811	0.4855	0.1559	0.9340
Incomplete college	1,233	0.0132	0.4987	0.0255	0.9594
Complete college	1,732	0.0186	0.5173	0.0358	0.9952
Espec./Residency	187	0.0020	0.5194	0.0039	0.9991
Masters	25	0.0003	0.5196	0.0005	0.9996
Doctoral	18	0.0002	0.5198	0.0004	1.0000
Not reported	44,690	0.4802	1.0000		
Total	93,068	1.0000			

Supplementary Table 2: Education level of *Pronto* patients age 25 or older

Supplementary Note 2 Computation details

This section describes variables used in the main manuscript. It also details computations in order to facilitate replication. A quick symbol reference can be seen in Supplementary Note 3. All computations were done in python using custom built scripts.

In our analysis, patients are denoted by $u \in U$ and drugs by $i, j \in D$; $U_i \in U$ is the subset of users who were dispensed drug i, $D^u \subseteq D$ is the subset of drugs dispensed to patient u, and $\nu^u \equiv |D^u|$ is the number of distinct drugs dispensed to patient u. Drugs are dispensed to patients in administration intervals $a = (i, t_s, t_f)$, where t_s and t_f are the start and end times (in days; $t \in \mathbb{N}$) of drug administration, and $(t_f - t_s)$ represents the total length of administration, respectively. The total number of drug i intervals dispensed to patient u is $\alpha_i^u = |A_i^u|$, where $A_i^u \equiv \{a_n^{i,u}\}$ is the set of administration intervals for patient u of drug i in the data, with $n = 1, \ldots, \alpha_i^u$.

Administration length. The total number of days patient u administered drug i (possibly over n distinct dispensations) is then computed as

$$\lambda_i^u = \sum_1^n a_n^{i,u} \quad . \tag{1}$$

Co-administration length. For each drug pair (i, j) administered to patient $u, \forall i, j \in D^u$, we identify the possible length of administration overlap between administrations of both drugs, $A_i^u \equiv \{a_n^{i,u}\}$ and $A_j^u \equiv \{a_m^{j,u}\}$, assuming without loss of generality that $t_{s,n} \leq t_{s,m}$, as

$$\lambda_{i,j}^u = \sum_{\substack{a_n \in A_i^u \\ a_m \in A_j^u}} \begin{cases} (t_{f,n} - t_{s,m}) \ , & \text{iff } (t_{f,n} < f_{f,m}) \\ (t_{f,m} - t_{s,m}) \ , & \text{otherwise} \end{cases} \quad . \tag{2}$$

Co-administrations. To be able to discriminate patients with a specific co-administration, and to compute how many were prescribed such co-administration, we define

$$\psi_{i,j}^u = \left(\lambda_{i,j}^u > 0\right) \quad , \tag{3}$$

a logical variable measuring whether patient u had at least one day of co-administration between drug pair (i, j); $\psi_{i,j}^u \in \{0, 1\}$. Then, the total number of co-administrations, per patient u or drug pair (i, j) is calculated as

$$\Psi^u = \sum_{i,j \in D^u} \psi_{i,j}^u \equiv |U^\Psi| \quad , \quad \Psi_{i,j} = \sum_{u \in U} \psi_{i,j}^u \equiv |U_{i,j}^\Psi| \quad . \tag{4}$$

Interactions. Next, we define a symmetrical binary map, also known as a symmetrical graph, $\Delta : D \times D \rightarrow \{0, 1\}$ on set D indicating if a drug pair $(i, j) \in D \times D$ has $(\delta_{i,j} = 1)$ a known DDI in DrugBank,

or not ($\delta_{i,j} = 0$). Then, to discriminate patients with a known DDI we define

$$\varphi^u_{i,j} = \left(\psi^u_{i,j} = 1 \wedge \delta_{i,j} = 1\right) \quad , \tag{5}$$

a logical variable measuring whether patient u had at least one day of co-administration between drug pair (i, j) *and* this drug pair is listed in DrugBank as a known DDI; $\varphi^u_{i,j} \in \{0, 1\}$. The total number of co-administrations of known DDI, per patient u or drug pair (i, j) is calculated as

$$\Phi^u = \sum_{i,j \in D^u} \varphi^u_{i,j} \equiv |U^\Phi| \quad , \quad \Phi_{i,j} = \sum_{u \in U} \varphi^u_{i,j} \equiv |U^\Phi_{i,j}| \quad . \tag{6}$$

Normalized interactions. To identify the drug pairs (i, j) with the largest "footprint" in the population, we compute the pairs that are most co-administered in the population: those pairs that maximize $|U^\Psi_{i,j}|$. Out of these, we are naturally most interested in the drug pairs that are known DDI and are most co-administered: those that maximize $|U^\Phi_{i,j}|$. Two asymmetrical normalized versions of this measure are computed as

$$\gamma^\Phi_{i,j} = \frac{|U^\Phi_{i,j}|}{|U_i|} \quad , \quad \gamma^\Phi_{j,i} = \frac{|U^\Phi_{i,j}|}{|U_j|} \quad , \tag{7}$$

which conditions the number of users co-administered drug pair (i, j) on the number of users that are administering either drug, i or j.

Normalized lengths. To obtain a normalized value of co-administration length, we also define

$$\tau^u_{i,j} = \frac{\lambda^u_{i,j}}{\lambda^u_i + \lambda^u_j - \lambda^u_{i,j}} \quad , \tag{8}$$

where $\tau^u_{i,j} \in [0, 1]$, and can be thought of a probability—or a Jaccard measure where values indicate a proximity [16–18]—of having drug pair (i, j) co-administered in relation to each drug's individual length of administration, for patient u.

Intuitively, if patient u always administers drugs i and j simultaneously, $\tau^u_{i,j} \to 1$. Conversely, drug pairs with small co-administration overlap have $\tau^u_{i,j} \to 0$. A normalized measure for the entire population is computed as

$$\tau^\Psi_{i,j} = \frac{\sum_{u \in U^\Psi_{i,j}} \tau^u_{i,j}}{|U^\Psi_{i,j}|} \quad , \tag{9}$$

where this proximity measure defines a weighted graph T^Φ [17] on set D where edges are $\tau^\Psi_{i,j} \in [0, 1]$ and link drugs that were co-administered in the population. $\tau^\Psi_{i,j}$ is larger when drug pairs (i, j) tend to be co-administered when either i or j is administered (correlated), and smaller otherwise (independent). To obtain a subgraph T^Φ, restricted to known DDI pairs, we compute $\tau^\Psi_{i,j} \times \delta_{i,j}$; thus T^Φ is a weighted version of Δ. In practice, due to computational complexity, we only compute $\tau^\Psi_{i,j}$ for drug pairs known to be a DDI ($\varphi^u_{i,j} > 0$).

Drug classes. For drug pairs co-administered and known to be DDI, we gathered their respective drug class hierarchy from Drugs.com [19]. In the main manuscript we used the top level of this hierarchy to distinguish different types of drugs (i.e., cardiovascular agents, hormones, etc). For example, Fluoxetine[2] has the following hierarchy: "Psychotherapeutic agents", "Antidepressants", and "Selective serotonin reuptake inhibitors", where the base class used was "Psychotherapeutic agents".

[2] https://www.drugs.com/fluoxetine.html

Supplementary Note 3 Notation and symbol reference

For quick reference, this chapter lists symbols used in the main manuscript and supplemental information.

symbol	description
U, u	Set of patients $u \in U \subset \mathbb{N}$ to whom at least one drug matched to DrugBank was dispensed.
D, i	Set of drugs $i \in D \subset \mathbb{N}$ available for dispensation in the public health care system of Blumenau; $D^u \subseteq D$ is the subset of drugs administered to patient u.
$A_i^u \equiv \{a_n^{i,u}\},\ a_n = (i, t_s, t_f)_n$	Set of administration intervals a_n. Each interval is defined as a n-tuple comprised of drug i and its administration start t_s (the dispensation), end time t_f, and administration length $t_t = (t_f - t_s)$, where $t \in \mathbb{N}$ (in days, n).
α_i^u	Number of drug intervals a_n (dispensations) to patient u. $\alpha_i^u = \|A_i^u\|$
ν^u	Number of distinct drugs dispensed to patient u. $\nu^u \equiv \|D^u\|$
λ_i^u	Administration length (in days) of drug i (across possibly multiple dispensations) for patient u.
$\lambda_{i,j}^u$	Co-administration length (in days) of drug pair (i, j) (across possibly multiple dispensations) for patient u. See Supplementary Equation 2 for overlap computation.
$\Delta, \delta_{i,j}$	$\Delta : D \times D \rightarrow 0, 1$ is a symmetrical binary relation (symmetrical graph) on set D, denoting the drug pairs having $(\delta_{i,j} = 1)$ a known DDI in DrugBank [20], or not $(\delta_{i,j} = 0)$; $\delta_{i,j} = \delta_{j,i}$, $\delta_{i,i} = 0$ (no self relation).
$\tau_{i,j}^u$	Normalized length of co-administration between drugs i and j for patient u. $\tau_{i,j}^u \in [0, 1]$ is a Jaccard measure between the number of days drug pair (i, j) was co-administered (intersection) divided by the number each drug, i and j was administered individually (union). $\tau_{i,j}^u = \lambda_{i,j}^u / (\lambda_i^u + \lambda_j^u - \lambda_{i,j}^u)$
Ω	The total city population. Also Ω^N and $\Omega^{y,g}$ are population numbers for a specific neighborhood N or for a certain age group y and gender g, respectively.

Supplementary Table 3: Basic symbols used in the paper

symbol	description
g	Gender where $g \in \{\mathrm{M}, \mathrm{F}\}$.
y	Age where $y \in \mathbb{N}$. y can also be grouped into age intervals (e.g., $y^{[0\text{-}4]}$, $y^{[5\text{-}9]}$, ..., $y^{[95\text{-}99]}$, $y^{[>99]}$) following IBGE–Instituto Brasileiro de Geografia e Estatística [7] convention.
N	Neighborhood $N \in \mathbb{N}$ in the city of Blumenau.
E	Education levels $N \in \mathbb{N}$ following IBGE–Instituto Brasileiro de Geografia e Estatística [7] convention.
s	DDI severity based on Drugs.com [19] classification. $s \in \{\mathrm{major}, \mathrm{moderate}, \mathrm{minor}, *, \mathrm{none}\}$

Supplementary Table 4: Symbols used in indexing.

symbol	description
α	Total number of administration intervals dispensed. $\alpha = \sum_{u \in U, i \in D^u} \alpha_i^u$
$\psi_{i,j}^u$	Logical variable denoting whether drug pair (i,j) was co-administered by patient u. $\psi_{i,j}^u = (\lambda_{i,j}^u > 0)$
Ψ^u	Number of distinct co-administrations for patient u. $\Psi^u = \sum_{i,j \in D^u} \psi_{i,j}^u$
$\Psi_{i,j}$	Number of distinct co-administrations between drug pair (i,j) for all patients. $\Psi_{i,j} = \sum_{u \in D^u} \psi_{i,j}^u \equiv \lvert U_{i,j} \rvert$
Ψ	Total number of drug co-administrations. $\Psi = \sum_u \Psi^u = \sum_{i,j} \Psi_{i,j}$
$\varphi_{i,j}^u$	Logical variable denoting whether drug pair (i,j) was co-administered to patient u *and* the pair is listed in DrugBank as a known DDI. $\varphi_{i,j}^u = (\psi_{i,j}^u > 0 \wedge \delta_{i,j} = 1)$
Φ^u	Number of distinct co-administration for patient u known to be a DDI. $\Phi^u = \sum_{i,j \in D^u} \varphi_{i,j}^u$
$\Phi_{i,j}$	Number of distinct co-administrations known to be a DDI between drug pair (i,j), for all patients. $\Phi_{i,j} = \sum_{u \in U} \varphi_{i,j}^u$
Φ	Total number of distinct drug interaction pairs. $\Phi = \sum_u \Phi^u = \sum_{i,j} \Phi_{i,j}$
$U^{\nu > x}$	Subset of patients who had at least $x \in \mathbb{N}$ distinct drugs administrations. $U^{\nu > x} = \{u \in U : \nu^u > x\}$.
U^{Ψ}	Subset of patients who had at least 1 drug co-administration. $U^{\Psi} = \{u \in U : \Psi^u > 0\}$.
$U_{i,j}^{\Psi}$	Subset of patients who were co-administered drug pair (i,j). $U_{i,j}^{\Psi} \equiv \{u \in U : \psi_{i,j}^u = 1\}$.
U^{Φ}	Subset of patients who had at least 1 known DDI. $U^{\Phi} = \{u \in U : \Phi^u > 0\}$.
$U_{i,j}^{\Phi}$	Subset of patients who were co-administered known DDI pair (i,j). $U_{i,j}^{\Phi} \equiv \{u \in U : \varphi_{i,j}^u = 1\}$.
U^g	Subset of patients per gender g. $U^g \equiv \{u \in U : gender(u) = g\}$.
$U^{[y_1,y_2]}$	Subset of patients per age bracket $[y_1, y_2]$. $U^{[y_1,y_2]} \equiv \{u \in U : age(u) \in [y_1, y_2]\}$.
U^N	Subset of patients per neighborhood N. $U^N \equiv \{u \in U : neighborhood(u) \in \mathbb{N}\}$.
U^E	Subset of patients per education level E. $U^{E=\varnothing}$ is the subset of patients who did not report their education level.
$\gamma_{i,j}^{\Psi}, \gamma_{j,i}^{\Psi}$	Normalized number of patients that were co-administering drug pair (i,j). $\gamma_{i,j}^{\Psi} = \lvert U_{i,j}^{\Psi} \rvert / \lvert U_i \rvert$ and $\gamma_{j,i}^{\Psi} = \lvert U_{i,j}^{\Psi} \rvert / \lvert U_j \rvert$. Note $\gamma_{i,j}^{\Psi} \neq \gamma_{j,i}^{\Psi}$.
$\gamma_{i,j}^{\Phi}, \gamma_{j,i}^{\Phi}$	Normalized number of patients that were co-administering drug pair (i,j), known to be a DDI. $\gamma_{i,j}^{\Phi} = \lvert U_{i,j}^{\Phi} \rvert / \lvert U_i \rvert$ and $\gamma_{j,i}^{\Phi} = \lvert U_{i,j}^{\Phi} \rvert / \lvert U_j \rvert$. Note $\gamma_{i,j}^{\Phi} \neq \gamma_{j,i}^{\Phi}$.
$\tau_{i,j}^{\Psi}$	Normalized length of co-administration of drug pair (i,j), for all patients. $\tau_{i,j}^{\Psi} = \sum_{u \in U_{i,j}^{\Psi}} \tau_{i,j}^u / \lvert U_{i,j}^{\Psi} \rvert$
$\tau_{i,j}^{\Phi}$	Normalized length of co-administration of drug pair (i,j), known to be a DDI, for all patients. $\tau_{i,j}^{\Phi} = \tau_{i,j}^{\Psi} \times \delta_{i,j}$

Supplementary Table 5: Administration, co-administration and interaction symbols

symbol	description
RRC^F , RRC^M	Relative risk of co-administration for women and men, respectively. For computation details see Supplementary Note 5.
RRI^F , RRI^M	Relative risk of interaction for women and men, respectively. For computation details see Supplementary Note 5.
$PI(i)$	Probability of interaction, or the propensity of a drug i to be involved in a DDI with all drugs it is co-administered with in the data. $PI(i) = \sum_j \Phi_{i,j} / \sum_j \Psi_{i,j}$

Supplementary Table 6: Relative Risks and Probabilities

Supplementary Note 4 Drug Interactions

This section lists DDI found in the analysis. Data source for these interactions were retrieved from http://wifo5-04.informatik.uni-mannheim.de/drugbank/. This dataset was last updated in 2011 and it contains the DrugBank ID for each pair of drugs and a textual description of the interaction. The latest (version 5.0) version of the DrugBank database includes a much larger number of interaction although much of the interaction at the top of the list could not be validated from a second source, namely Drugs.com [19]. Thus we opted for a more conservative approach with fewer number of overall unique interaction that we could attribute a severity score from a second data source.

From Drugs.com [19], the description of each severity score is as follow:

- *Major*: Highly clinically significant. Avoid combinations; the risk of the interaction outweighs the benefit.
- *Moderate*: Moderately clinically significant. Usually avoid combinations; use it only under special circumstances.
- *Minor*: Minimally clinically significant. Minimize risk; assess risk and consider an alternative drug, take steps to circumvent the interaction risk and/or institute a monitoring plan.

Note that some interactions present in DrugBrank were not found in Drugs.com. These are marked as *None*.

rank_Φ	$\lvert U^\Phi_{i,j}\rvert$	$\gamma^\Phi_{i,j}$	$\tau^\Phi_{i,j}$	$\langle\lambda^u_{i,j}\rangle$	i	j	$RRI^F_{i,j}$	severity	interaction
1	5078	0.19	0.26	102 ± 95	Omeprazole	Clonazepam	2.28	Moderate	Omeprazole increases the effect of benzodiazepine
2	2117	0.18	0.23	53 ± 74	ASA	Ibuprofen	1.42	Major	Ibuprofen reduces ASA cardioprotective effects
3	1460	0.20	0.21	54 ± 77	Atenolol	Ibuprofen	1.88	Moderate	Risk of inhibition of renal prostaglandins
4	1249	0.10	0.60	141 ± 124	ASA	Glyburide	0.89	Moderate	The salicylate increases the effect of sulfonylurea
5	1190	0.19	0.45	127 ± 127	Amitriptyline	Fluoxetine	3.55	Major	Fluoxetine increases the effect and toxicity of tricyclics
6	999	0.04	0.27	87 ± 86	Omeprazole	Diazepam	1.21	Moderate	Omeprazole increases the effect of benzodiazepine
7	892	0.14	0.20	56 ± 61	Fluconazole	Simvastatin	2.63	Major	Increased risk of myopathy/rhabdomyolysis
8	752	0.06	0.12	30 ± 50	ASA	Dexamethasone	1.30	Moderate	The corticosteroid decreases the effect of salicylates
9	627	0.10	0.16	46 ± 54	Fluconazole	Clonazepam	3.40	None	Increases the effect of the benzodiazepine
10	609	0.07	0.19	48 ± 93	Prednisone	ASA	0.94	Moderate	The corticosteroid decreases the effect of salicylates
11	535	0.07	0.58	152 ± 132	Atenolol	Glyburide	1.22	Moderate	The beta-blocker decreases the symptoms of hypoglycemia
12	524	0.50	0.70	243 ± 188	Haloperidol	Biperiden	0.62	Moderate	Anticholinergic inc. risk of psychosis and tardive dyskinesia
13	501	0.21	0.25	44 ± 62	Propranolol	Ibuprofen	3.42	Moderate	Risk of inhibition of renal prostaglandins
14	500	0.15	0.20	52 ± 75	Furosemide	Ibuprofen	1.93	Moderate	NSAID decreases diuretic and antihypertensive effects of loop diuretic
15	496	0.04	0.36	103 ± 87	ASA	Gliclazide	0.78	None	The salicylate increases the effect of sulfonylurea
16	470	0.63	0.55	160 ± 133	Diltiazem	Simvastatin	1.27	Major	Increases the effect and toxicity of simvastatin
17	385	0.59	0.60	155 ± 125	Digoxin	Furosemide	0.61	Moderate	Possible electrolyte variations and arrhythmias
18	377	0.03	0.50	143 ± 138	Fluoxetine	Carbamazepine	0.98	Moderate	Increases the effect of carbamazepine
19	364	0.17	0.28	110 ± 106	Carbamazepine	Simvastatin	0.94	Moderate	Decreases the effect of the statin
20	355	0.03	0.26	86 ± 84	Fluoxetine	Propranolol	4.76	Moderate	The SSRI increases the effect of the beta-blocker
21	284	0.04	0.27	66 ± 57	Levothyroxine	Iron (II) Sulfate	4.59	Moderate	Iron decreases absorption of levothyroxine
22	272	0.42	0.55	140 ± 114	Digoxin	Spironolactone	0.58	Minor	Increased digoxin levels and decreased effect with spironolactone
23	257	0.16	0.42	123 ± 130	Imipramine	Fluoxetine	3.08	Major	Fluoxetine increases the effect and toxicity of tricyclics
24	245	0.04	0.19	42 ± 40	Fluconazole	Amitriptyline	4.25	Moderate	The imidazole increases the effect and toxicity of the tricyclic
25	244	0.01	0.22	57 ± 77	Acetaminophen	Warfarin	1.07	Minor	Acetaminophen increases the anticoagulant effect
26	226	0.04	0.49	151 ± 145	Amitriptyline	Carbamazepine	0.99	Moderate	The tricyclics increases the effect of carbamazepine
27	222	0.02	0.47	148 ± 139	Fluoxetine	Lithium	1.79	Major	The SSRI increases serum levels of lithium
28	201	0.03	0.34	107 ± 95	Atenolol	Gliclazide	1.09	None	The beta-blocker decreases the symptoms of hypoglycemia
29	186	0.18	0.43	142 ± 156	Haloperidol	Carbamazepine	0.62	Moderate	Carbamazepine decreases the effect of haloperidol
30	179	0.08	0.09	10 ± 6	Ethinyl Estradiol	Amoxicillin	126.09	Moderate	Anti-infectious agent could decrease effect of oral contraceptive
31	173	0.27	0.41	109 ± 96	Digoxin	Carvedilol	0.53	Moderate	Carvedilol increases levels/effect of digoxin
32	155	0.02	0.22	68 ± 80	Amitriptyline	Salbutamol	2.83	Moderate	The tricyclic increases the sympathomimetic effect
33	154	0.02	0.45	144 ± 122	Levothyroxine	Warfarin	1.05	Moderate	Thyroid hormones increase the anticoagulant effect
33	154	0.01	0.33	94 ± 92	Fluoxetine	Nortriptyline	2.70	Major	Fluoxetine increases the effect and toxicity of tricyclics
35	149	0.28	0.32	115 ± 109	Phenytoin	Omeprazole	0.80	Moderate	Omeprazole increases the effect of hydantoin
36	148	0.14	0.49	168 ± 160	Haloperidol	Lithium	1.31	Major	Possible extrapyramidal effects and neurotoxicity
37	147	0.02	0.23	60 ± 76	Atenolol	Salbutamol	1.37	Moderate	Antagonism
38	130	0.00	0.08	27 ± 45	Ibuprofen	Lithium	2.08	Moderate	The NSAID increases serum levels of lithium
39	123	0.00	0.16	31 ± 43	Ibuprofen	Carvedilol	0.88	Moderate	Risk of inhibition of renal prostaglandins
40	117	0.18	0.46	126 ± 127	Digoxin	Hydrochlorothiazide	0.95	Moderate	Possible electrolyte variations and arrhythmias
41	116	0.02	0.14	9 ± 7	Norfloxacin	Iron (II) Sulfate	6.14	Moderate	Formation of non-absorbable complexes
42	103	0.16	0.43	113 ± 110	Digoxin	Levothyroxine	1.50	Moderate	The thyroid hormones decreases the effect of digoxin
42	103	0.01	0.23	76 ± 80	Fluoxetine	Carvedilol	1.50	Moderate	The SSRI increases the effect of the beta-blocker
44	102	0.01	0.04	4 ± 4	Diclofenac	Alendronate	9.61	Moderate	Increased risk of gastric toxicity
45	101	0.01	0.25	92 ± 81	Fluoxetine	Warfarin	1.46	Moderate	The SSRI increases the effect of anticoagulant
46	95	0.04	0.57	140 ± 126	Propranolol	Glyburide	1.61	Moderate	The beta-blocker decreases the symptoms of hypoglycemia
47	91	0.01	0.52	154 ± 142	Atenolol	Diltiazem	1.19	Major	Increased risk of bradycardia
48	90	0.06	0.50	161 ± 157	Imipramine	Carbamazepine	1.35	Moderate	The tricyclic increases the effect of carbamazepine
49	89	0.01	0.17	36 ± 32	Fluconazole	Diazepam	2.16	Moderate	Increases the effect of the benzodiazepine
50	84	0.01	0.09	15 ± 26	Prednisone	Ethinyl Estradiol	58.79	Moderate	The estrogenic agent increases the effect of corticosteroid

Supplementary Table 7: DDI list 1-50. Complete list of known DDI pairs (i,j) by rank of $U^\Phi_{i,j}$, the number of patients affects by the DDI (1st and 2nd columns, respectively). The normalized drug pair footprint in the population ($\gamma^\Phi_{i,j}$) as well as the normalized co-administration length ($\tau^\Phi_{i,j}$), are shown in columns 3 and 4, respectively. Mean (± s.d.) co-administration length, $\langle\lambda^u_{i,j}\rangle$, is shown in column 5 (in days) for each DDI pair (i,j) whose English drug names are shown in columns 6 and 7. The relative gender risk of DDI pair co-administration, $RRI^F_{i,j}$, shown in column 8. DDI severity classification, according to *Drugs.com*, shown in column 9; DDIs or drugs not found in *Drugs.com* are labeled as *None* or *, respectively. Drug pair interaction, according to *DrugBank*, shown in column 10. Continues on Supplementary Table 8.

$rank_{\Phi}$	$\|U^{\Phi}_{i,j}\|$	$\gamma^{\Phi}_{i,j}$	$\tau^{\Phi}_{i,j}$	$\langle \lambda^{u}_{i,j} \rangle$	i	j	$RRI^{F}_{i,j}$	severity	interaction
51	71	0.13	0.47	169 ± 151	Phenytoin	Fluoxetine	0.73	Moderate	Fluoxetine increases the effect of phenytoin
51	71	0.01	0.08	15 ± 19	Atenolol	Fenoterol	2.64	*	Antagonism
53	69	0.02	0.16	10 ± 8	Ciprofloxacin	Iron (II) Sulfate	4.18	Moderate	Formation of non-absorbable complexes
54	63	0.17	0.35	33 ± 28	Methyldopa	Iron (II) Sulfate	21.60	Moderate	Iron decreases the absorption of dopa derivatives
54	63	0.08	0.34	110 ± 118	Diltiazem	Amlodipine	1.52	Moderate	Increases the effect and toxicity of amlodipine
56	60	0.01	0.19	49 ± 95	Prednisone	Warfarin	0.76	Moderate	The corticosteroid alters the anticoagulant effect
56	60	0.01	0.12	28 ± 43	Amitriptyline	Fenoterol	2.83	*	The tricyclic increases the sympathomimetic effect
58	59	0.01	0.15	34 ± 34	Fluconazole	Carbamazepine	1.03	Moderate	Increases the effect of carbamazepine
59	58	0.05	0.03	5 ± 10	Hydrocortisone	ASA	1.35	Moderate	The corticosteroid decreases the effect of salicylates
60	57	0.01	0.15	50 ± 48	Fluconazole	Imipramine	7.37	Moderate	The imidazole increases the effect and toxicity of the tricyclic
60	57	0.02	0.35	96 ± 96	Glyburide	Carvedilol	0.73	Moderate	The beta-blocker decreases the symptoms of hypoglycemia
62	52	0.08	0.49	118 ± 114	Digoxin	Amiodarone	0.56	Major	Amiodarone increases the effect of digoxin
63	51	0.00	0.23	93 ± 90	Hydrochlorothiazide	Lithium	2.90	Major	The thiazide diuretic increases serum levels of lithium
64	48	0.00	0.16	65 ± 74	Enalapril	Lithium	1.91	Moderate	The ACE inhibitor increases serum levels of lithium
65	47	0.04	0.46	135 ± 109	Allopurinol	Warfarin	0.19	Moderate	Allopurinol increases the anticoagulant effect
66	44	0.03	0.16	51 ± 61	Imipramine	Salbutamol	3.19	Moderate	The tricyclic increases the sympathomimetic effect
67	43	0.08	0.40	144 ± 153	Phenytoin	Diazepam	0.62	Moderate	Possible increased levels of the hydantoin, decrease of benzodiazepine
68	41	0.00	0.21	82 ± 74	Losartan	Lithium	4.13	Moderate	Losartan increases serum levels of lithium
69	39	0.04	0.42	82 ± 64	Gliclazide	Carvedilol	0.67	None	The beta-blocker decreases the symptoms of hypoglycemia
69	39	0.14	0.15	30 ± 46	Timolol	Ibuprofen	1.42	None	Risk of inhibition of renal prostaglandins
69	39	0.06	0.42	130 ± 122	Nortriptyline	Carbamazepine	1.26	Moderate	The tricyclic increases the effect of carbamazepine
72	36	0.05	0.05	15 ± 14	Phenobarbital	Dexamethasone	1.11	Moderate	The barbiturate decreases the effect of the corticosteroid
73	31	0.01	0.04	9 ± 8	Prednisolone	ASA	2.95	Moderate	The corticosteroid decreases the effect of salicylates
73	31	0.01	0.20	48 ± 66	Propranolol	Salbutamol	6.61	Major	Antagonism
75	30	0.00	0.09	10 ± 3	Clavulanate	Ethinyl Estradiol	inf	None	Anti-infectious agent could decrease effect of oral contraceptive
76	28	0.04	0.22	83 ± 86	Digoxin	Diazepam	1.09	Moderate	The benzodiazepine increases the effect of digoxin
77	27	0.01	0.30	81 ± 73	Propranolol	Gliclazide	2.02	None	The beta-blocker decreases the symptoms of hypoglycemia
77	27	0.03	0.19	11 ± 6	Doxycycline	Ethinyl Estradiol	inf	Moderate	Anti-infectious agent could decrease effect of oral contraceptive
77	27	0.05	0.05	10 ± 6	Phenytoin	Ciprofloxacin	0.49	Moderate	Ciprofloxacin decreases the hydantoin effect
77	27	0.01	0.05	7 ± 3	Carbamazepine	Metronidazole	1.68	Moderate	Metronidazole increases the effect of carbamazepine
77	27	0.00	0.07	8 ± 7	Prednisone	Estradiol	inf	Moderate	The estrogenic agent increases the effect of corticosteroid
82	26	0.00	0.20	44 ± 70	Fluconazole	Nortriptyline	5.43	Moderate	The imidazole increases the effect and toxicity of the tricyclic
82	26	0.05	0.07	14 ± 12	Phenytoin	Dexamethasone	1.13	Moderate	The enzyme inducer decreases the effect of the corticosteroid
84	25	0.03	0.56	157 ± 136	Diltiazem	Amiodarone	1.26	Major	Increased risk of cardiotoxicity and arrhythmias
85	24	0.01	0.17	15 ± 10	Propranolol	Fenoterol	3.54	*	Antagonism
85	24	0.01	0.29	100 ± 85	Carbamazepine	Warfarin	0.99	Moderate	Decreases the anticoagulant effect
85	24	0.00	0.05	3 ± 2	Diclofenac	Warfarin	0.84	Major	The NSAID increases the anticoagulant effect
85	24	0.04	0.15	29 ± 45	Phenytoin	Prednisone	1.72	Moderate	The enzyme inducer decreases the effect of the corticosteroid
89	23	0.03	0.47	152 ± 143	Diltiazem	Propranolol	2.01	Major	Increased risk of bradycardia
89	23	0.00	0.16	36 ± 44	Fluconazole	Haloperidol	1.33	Major	The imidazole increases the effect and toxicity of haloperidol
91	22	0.05	0.20	19 ± 28	Estrogens Conj.	Prednisone	inf	Moderate	The estrogenic agent increases the effect of corticosteroid
91	22	0.00	0.07	9 ± 4	Ciprofloxacin	Warfarin	1.02	Major	The quinolone increases the anticoagulant effect
93	21	0.01	0.08	10 ± 6	Tobramycin	Furosemide	3.01	Major	Increased ototoxicity
93	21	0.02	0.33	94 ± 116	Chlorpromazine	Propranolol	1.77	Moderate	Increased effect of both drugs
95	19	0.00	0.07	26 ± 35	Prednisone	Phenobarbital	1.53	Moderate	The barbiturate decreases the effect of the corticosteroid
95	19	0.00	0.07	10 ± 7	Ciprofloxacin	Aminophylline	1.21	Major	The quinolone increases the effect of theophylline
97	18	0.00	0.13	33 ± 44	Fluconazole	Warfarin	0.89	Major	Increases the anticoagulant effect
98	17	0.03	0.24	50 ± 48	Nortriptyline	Salbutamol	1.70	Moderate	The tricyclic increases the sympathomimetic effect
98	17	0.01	0.26	107 ± 85	Propranolol	Phenobarbital	1.01	Moderate	The barbiturate decreases the effect of metabolized beta-blocker
100	16	0.02	0.24	46 ± 29	Haloperidol	Propranolol	1.56	Moderate	Increased effect of both drugs

Supplementary Table 8: DDI list 51-100. See Supplementary Table 7 for column description. Continues on Supplementary Table 9.

rank_Φ	$\lvert U^{\Phi}_{i,j}\rvert$	$\gamma^{\Phi}_{i,j}$	$\tau^{\Phi}_{i,j}$	$\langle \lambda^{u}_{i,j} \rangle$	i	j	$RRI^{F}_{i,j}$	severity	interaction
100	16	0.00	0.07	6 ± 3	Azithromycin	Warfarin	1.18	Moderate	Increases the anticoagulant effect
100	16	0.00	0.08	16 ± 21	Metronidazole	Lithium	4.96	Moderate	Metronidazole increases the effect and toxicity of lithium
100	16	0.03	0.31	94 ± 83	Phenytoin	Furosemide	0.55	Minor	The hydantoin decreases the effect of furosemide
104	15	0.01	0.13	20 ± 17	Carvedilol	Fenoterol	0.47	*	Antagonism
105	14	0.02	0.21	5 ± 3	Doxycycline	Amoxicillin	0.53	Moderate	Possible antagonism of action
105	14	0.00	0.12	9 ± 7	Furosemide	Gentamicin	0.71	Major	Increased ototoxicity
105	14	0.00	0.23	63 ± 59	Fluconazole	Phenytoin	1.28	Moderate	Increases the effect of hydantoin
105	14	0.03	0.23	88 ± 71	Phenytoin	Warfarin	0.94	Moderate	Increased hydantoin levels and risk of bleeding
109	13	0.01	0.14	35 ± 26	Carbamazepine	Ethinyl Estradiol	inf	Major	This product might cause a slight decrease of contraceptive effect
109	13	0.01	0.14	35 ± 26	Levonorgestrel	Carbamazepine	inf	Major	Carbamazepine decreases the contraceptive effect
109	13	0.01	0.56	122 ± 113	Propranolol	Methyldopa	8.50	Major	Possible hypertensive crisis
109	13	0.05	0.19	55 ± 59	Timolol	Glyburide	1.13	Moderate	The beta-blocker decreases the symptoms of hypoglycemia
113	12	0.01	0.18	72 ± 98	Captopril	Lithium	1.42	Moderate	The ACE inhibitor increases serum levels of lithium
114	11	0.01	0.08	33 ± 63	Imipramine	Fenoterol	7.08	*	The tricyclic increases the sympathomimetic effect
114	11	0.01	0.24	57 ± 46	Methylphenidate	Carbamazepine	0.07	None	Carbamazepine could reduce the effect of methylphendiate
114	11	0.00	0.15	14 ± 21	Norfloxacin	Aminophylline	7.08	Moderate	The quinolone increases the effect of theophylline
117	10	0.02	0.04	11 ± 4	Erythromycin	Simvastatin	2.83	Major	The macrolide possibly increases the statin toxicity
118	9	0.00	0.03	7 ± 1	Prednisolone	Phenobarbital	0.89	Moderate	The barbiturate decreases the effect of the corticosteroid
118	9	0.00	0.29	62 ± 46	Folic acid	Phenytoin	1.42	Moderate	Folic acid decreases the levels of hydantoin
118	9	0.00	0.11	16 ± 9	Metoclopramide	Levodopa	5.67	Moderate	Levodopa decreases the effect of metoclopramide
118	9	0.00	0.29	72 ± 128	Carbamazepine	Norethisterone	inf	Major	This product may cause a slight decrease of contraceptive effect
118	9	0.03	0.15	51 ± 91	Timolol	Salbutamol	0.89	Major	Antagonism
123	8	0.01	0.22	67 ± 36	Diltiazem	Carbamazepine	0.71	Major	Increases the effect of carbamazepine
123	8	0.00	0.04	6 ± 2	Metronidazole	Phenobarbital	1.18	Moderate	The barbiturate decreases the effect of metronidazole
123	8	0.02	0.33	53 ± 40	Methyldopa	Salbutamol	4.96	Moderate	Increased arterial pressure
123	8	0.00	0.25	82 ± 54	Carbamazepine	Aminophylline	0.71	Moderate	Increases or decreases the effect of theophylline
127	7	0.01	0.16	62 ± 97	Phenytoin	Trimethoprim	0.94	Moderate	Trimethoprim increases the effect of hydantoin
127	7	0.00	0.23	122 ± 123	Propranolol	Maprotiline	4.25	Minor	Propranolol increases the serum levels of cisapride
127	7	0.01	0.11	25 ± 15	Nortriptyline	Fenoterol	1.77	*	The tricyclic increases the sympathomimetic effect
127	7	0.02	0.09	13 ± 8	Methyldopa	Fenoterol	4.25	None	Increased arterial pressure
127	7	0.01	0.20	11 ± 4	Doxycycline	Iron (II) Sulfate	inf	Moderate	Formation of non-absorbable complexes
132	5	0.00	0.55	82 ± 86	Propranolol	Aminophylline	1.06	Major	Antagonism of action and increased effect of theophylline
132	5	0.04	0.45	221 ± 207	Propylthiouracil	Warfarin	1.06	Moderate	The anti-thyroid agent causes variations in the anticoagulant effect
132	5	0.01	0.09	9 ± 4	Doxycycline	Carbamazepine	2.83	Moderate	The anticonvulsant decreases the effect of doxycycline
132	5	0.02	0.22	34 ± 26	Timolol	Gliclazide	1.06	None	The beta-blocker decreases the symptoms of hypoglycemia
132	5	0.00	0.17	10 ± 6	Prednisolone	Ethinyl Estradiol	inf	Moderate	The estrogenic agent increases the effect of the corticosteroid
132	5	0.00	0.31	162 ± 120	Medroxyproges. Ac.	Phenobarbital	inf	Moderate	The enzyme inducer decreases the effect of hormones
132	5	0.01	0.35	107 ± 123	Phenytoin	Amiodarone	0.18	Moderate	Amiodarone increases the effect of hydantoin
132	5	0.00	0.21	104 ± 153	Folic acid	Phenobarbital	2.83	Moderate	Folic acid decreases the effect of anticonvulsant
140	4	0.00	0.16	40 ± 29	Levonorgestrel	Phenobarbital	inf	Major	Phenobarbital decreases the effect of levonorgestrel
140	4	0.00	0.26	72 ± 61	Atenolol	Verapamil	0.00	Major	Increased effect of both drugs
140	4	0.01	0.14	10 ± 2	Estrogens Conj.	Prednisolone	inf	Moderate	The estrogenic agent increases the effect of corticosteroid
140	4	0.00	0.14	4 ± 3	Doxycycline	Clavulanate	0.71	None	Possible antagonism of action
140	4	0.00	0.17	62 ± 44	Phenobarbital	Aminophylline	0.24	Moderate	The barbiturate decreases the effect of theophylline
145	3	0.01	0.18	97 ± 94	Estrogens Conj.	Phenobarbital	inf	Moderate	The enzyme inducer decreases the effect of hormones
145	3	0.00	0.32	136 ± 117	Ethinyl Estradiol	Aminophylline	inf	Moderate	The contraceptive increases the effect and toxicity of theophylline
145	3	0.00	0.20	53 ± 16	Ethinyl Estradiol	Phenobarbital	inf	Major	This product may cause a slight decrease of contraceptive effect
145	3	0.00	0.22	45 ± 43	Medroxyproges. Ac.	Warfarin	inf	None	The agent increases the effect of anticoagulant
145	3	0.00	0.01	2 ± 0	Hydrocortisone	Phenobarbital	1.42	Moderate	The barbiturate decreases the effect of the corticosteroid
145	3	0.00	0.23	11 ± 9	Doxycycline	Warfarin	1.42	Moderate	The tetracycline increases the anticoagulant effect

Supplementary Table 9: DDI list 101-150. See Supplementary Table 7 for column description. Continues on Supplementary Table 10.

rank_Φ	$\lvert U^\Phi_{i,j}\rvert$	$\gamma^\Phi_{i,j}$	$\tau^\Phi_{i,j}$	$\langle\lambda^u_{i,j}\rangle$	i	j	$RRI^F_{i,j}$	severity	interaction
145	3	0.01	0.12	23 ± 13	Phenytoin	Aminophylline	1.42	Moderate	Decreased effect of both products
145	3	0.01	0.43	40 ± 57	Methyldopa	Levodopa	inf	Minor	Methyldopa increases the effect and toxicity of levodopa
145	3	0.00	0.12	40 ± 19	Digoxin	Verapamil	inf	Moderate	Verapamil increases the effect of digoxin
145	3	0.00	0.15	58 ± 56	Aminophylline	Lithium	1.42	Moderate	Theophylline decreases serum levels of lithium
145	3	0.01	0.10	9 ± 4	Phenytoin	Prednisolone	0.35	Moderate	The enzyme inducer decreases the effect of the corticosteroid
145	3	0.01	0.43	185 ± 98	Phenytoin	Levodopa	0.00	Moderate	The hydantoin decreases the effect of levodopa
157	2	0.00	0.06	6 ± 1	Estradiol	Prednisolone	inf	Moderate	The estrogenic agent increases the effect of corticosteroid
157	2	0.01	0.13	31 ± 0	Timolol	Aminophylline	inf	Major	Antagonism of action and increased effect of theophylline
157	2	0.00	0.24	62 ± 53	Phenytoin	Estradiol	inf	Moderate	The enzyme inducer decreases the effect of the hormones
157	2	0.00	0.08	23 ± 11	Doxycycline	Phenobarbital	inf	Moderate	The anticonvulsant decreases the effect of doxycycline
157	2	0.00	0.20	79 ± 30	Norethisterone	Phenobarbital	inf	Major	This product may cause a slight decrease of contraceptive effect
157	2	0.00	0.20	79 ± 30	Estradiol	Phenobarbital	inf	Moderate	The enzyme inducer decreases the effect of hormones
157	2	0.00	0.42	102 ± 110	Propranolol	Verapamil	0.71	Major	Increased effect of both drugs
157	2	0.01	0.03	2 ± 0	Timolol	Fenoterol	inf	*	Antagonism
157	2	0.00	0.02	2 ± 0	Phenytoin	Hydrocortisone	0.71	Moderate	The enzyme inducer decreases the effect of the corticosteroid
157	2	0.00	0.53	288 ± 213	Phenytoin	Medroxyproges. Ac.	inf	Moderate	The enzyme inducer decreases the effect of the hormones
157	2	0.00	0.24	62 ± 53	Phenytoin	Norethisterone	inf	Major	This product may cause a slight decrease of contraceptive effect
157	2	0.00	0.42	274 ± 218	Digoxin	Propylthiouracil	0.71	Moderate	The antithyroid agent increases the effect of digoxin
169	1	0.00	0.00	2 ± 0	Atenolol	Epinephrine	inf	Moderate	Hypertension, then bradycardia
169	1	0.00	0.49	179 ± 0	Phenytoin	Ethinyl Estradiol	inf	Major	This product may cause a slight decrease of contraceptive effect
169	1	0.00	0.30	117 ± 0	Haloperidol	Methyldopa	inf	Moderate	Methyldopa increases haloperidol effect or risk of psychosis
169	1	0.00	0.49	179 ± 0	Phenytoin	Levonorgestrel	inf	Major	Phenytoin decreases the contraceptive effect
169	1	0.00	0.51	31 ± 0	Phenytoin	Sulfadiazine	0.00	Moderate	The sulfonamide increases the effect of hydantoin
169	1	0.00	0.02	4 ± 0	Erythromycin	Aminophylline	inf	Moderate	The macrolide increases the effect and toxicity of theophylline
169	1	0.00	0.24	12 ± 0	Timolol	Methyldopa	inf	Major	Possible hypertensive crisis
169	1	0.00	0.05	6 ± 0	Erythromycin	Carbamazepine	0.00	Major	The macrolide increases the effect of carbamazepine
169	1	0.00	0.06	2 ± 0	Erythromycin	Diazepam	inf	Moderate	The macrolide increases the effect of the benzodiazepine
169	1	0.00	0.03	9 ± 0	Erythromycin	Fluoxetine	inf	Moderate	Possible serotoninergic syndrome with this combination
169	1	0.00	0.25	15 ± 0	Phenytoin	Doxycycline	inf	Moderate	The anticonvulsant decreases the effect of doxycycline
169	1	0.00	0.06	29 ± 0	Phenytoin	Estrogens Conj.	inf	Moderate	The enzyme inducer decreases the effect of the hormones
169	1	0.00	0.31	124 ± 0	Carbamazepine	Verapamil	0.00	Major	Verapamil increases the effect of carbamazepine

Supplementary Table 10: DDI list 151-181. See Supplementary Table 7 for column description.

rank_Φ	$\lvert U^\Phi_{i,j}\rvert$	$\gamma^\Phi_{i,j}$	$\tau^\Phi_{i,j}$	$\langle\lambda^u_{i,j}\rangle$	i	j	$RRI^F_{i,j}$	severity
2	2117	0.18	0.23	53 ± 74	ASA	Ibuprofen	1.42	Major
5	1190	0.19	0.45	127 ± 127	Amitriptyline	Fluoxetine	3.55	Major
7	892	0.14	0.20	56 ± 61	Fluconazole	Simvastatin	2.63	Major
16	470	0.63	0.55	160 ± 133	Diltiazem	Simvastatin	1.27	Major
23	257	0.16	0.42	123 ± 130	Imipramine	Fluoxetine	3.08	Major
27	222	0.02	0.47	148 ± 139	Fluoxetine	Lithium	1.79	Major
33	154	0.01	0.33	94 ± 92	Fluoxetine	Nortriptyline	2.70	Major
36	148	0.14	0.49	168 ± 160	Haloperidol	Lithium	1.31	Major
47	91	0.01	0.52	154 ± 142	Atenolol	Diltiazem	1.19	Major
62	52	0.08	0.49	118 ± 114	Digoxin	Amiodarone	0.56	Major
63	51	0.00	0.23	93 ± 90	Hydrochlorothiazide	Lithium	2.90	Major
73	31	0.01	0.20	48 ± 66	Propranolol	Salbutamol	6.61	Major
84	25	0.03	0.56	157 ± 136	Diltiazem	Amiodarone	1.26	Major
85	24	0.00	0.05	3 ± 2	Diclofenac	Warfarin	0.84	Major
89	23	0.03	0.47	152 ± 143	Diltiazem	Propranolol	2.01	Major
89	23	0.00	0.16	36 ± 44	Fluconazole	Haloperidol	1.33	Major
91	22	0.00	0.07	9 ± 4	Ciprofloxacin	Warfarin	1.02	Major
93	21	0.01	0.08	10 ± 6	Tobramycin	Furosemide	3.01	Major
95	19	0.00	0.07	10 ± 7	Ciprofloxacin	Aminophylline	1.21	Major
97	18	0.00	0.13	33 ± 44	Fluconazole	Warfarin	0.89	Major

Supplementary Table 11: Top 20 *major* DDI pairs (i, j) by rank of $|U^\Phi_{i,j}|$, the number of patients affects by the DDI (1st and 2nd columns, respectively). The normalized drug pair footprint in the population ($\gamma^\Phi_{i,j}$) as well as the normalized co-administration length ($\tau^\Phi_{i,j}$), are shown in columns 3 and 4, respectively. Mean (± s.d.) co-administration length, $\langle\lambda^u_{i,j}\rangle$, is shown in column 5 (in days) for each DDI pair (i, j) whose English drug names are shown in columns 6 and 7. The relative gender risk of DDI pair co-administration, $RRI^F_{i,j}$, is shown in column 8. DDI severity classification, according to *Drugs.com*, shown in column 9.

rankp(γ)	$\gamma^\Phi_{i,j}$	$\gamma^\Phi_{j,i}$	$\lvert U^\Phi_{i,j}\rvert$	$\langle\lambda^u_{i,j}\rangle$	i	j	$RRI^F_{i,j}$	severity
1	0.50	0.61	524	243 ± 188	Haloperidol	Biperiden	0.62	Moderate
2	0.59	0.12	385	155 ± 125	Digoxin	Furosemide	0.61	Moderate
3	0.19	0.36	5078	102 ± 95	Omeprazole	Clonazepam	2.28	Moderate
4	0.10	0.50	1249	141 ± 124	ASA	Glyburide	0.89	Moderate
5	0.42	0.14	272	140 ± 114	Digoxin	Spironolactone	0.58	Minor
6	0.63	0.02	470	160 ± 133	Diltiazem	Simvastatin	1.27	Major
7	0.27	0.15	173	109 ± 96	Digoxin	Carvedilol	0.53	Moderate
8	0.04	0.44	496	103 ± 87	ASA	Gliclazide	0.78	None
9	0.04	0.31	999	87 ± 86	Omeprazole	Diazepam	1.21	Moderate
9	0.19	0.09	1190	127 ± 127	Amitriptyline	Fluoxetine	3.55	Major
11	0.14	0.16	148	168 ± 160	Haloperidol	Lithium	1.31	Major
12	0.07	0.22	535	152 ± 132	Atenolol	Glyburide	1.22	Moderate
13	0.18	0.08	186	142 ± 156	Haloperidol	Carbamazepine	0.62	Moderate
14	0.18	0.06	2117	53 ± 74	ASA	Ibuprofen	1.42	Major
14	0.20	0.04	1460	54 ± 77	Atenolol	Ibuprofen	1.88	Moderate
16	0.02	0.24	222	148 ± 139	Fluoxetine	Lithium	1.79	Major
17	0.03	0.18	201	107 ± 95	Atenolol	Gliclazide	1.09	None
18	0.01	0.26	154	94 ± 92	Fluoxetine	Nortriptyline	2.70	Major
19	0.28	0.00	149	115 ± 109	Phenytoin	Omeprazole	0.80	Moderate
20	0.03	0.17	377	143 ± 138	Fluoxetine	Carbamazepine	0.98	Moderate

Supplementary Table 12: Top 20 known DDI pairs (i, j) by rank product (1st column) of the ranks of $\gamma^\Phi_{i,j}$ and $\gamma^\Phi_{j,i}$, the normalized drug pair footprint in the population (1st, 2nd and 3rd columns, respectively). The number of patients affected by the drug pair, $|U^\Phi_{i,j}|$, is shown in column 4. Mean (± s.d.) co-administration length, $\langle\lambda^u_{i,j}\rangle$, is shown in column 5 (in days) for each DDI pair (i, j) whose English drug names are shown in columns 6 and 7. The relative gender risk of DDI pair co-administration, $RRI^F_{i,j}$, is shown in column 8. DDI severity classification, according to *Drugs.com*, shown in column 9; DDIs or drugs not found in *Drugs.com* are labeled as *None* or ***, respectively.

rank_τ	$\tau^{\Phi}_{i,j}$	$\lvert U^{\Phi}_{i,j}\rvert$	$\langle\lambda^{u}_{i,j}\rangle$	i	j	$RRI^{F}_{i,j}$	severity
1	0.70	524	243 ± 188	Haloperidol	Biperiden	0.62	Moderate
2	0.60	1249	141 ± 124	ASA	Glyburide	0.89	Moderate
3	0.60	385	155 ± 125	Digoxin	Furosemide	0.61	Moderate
4	0.58	535	152 ± 132	Atenolol	Glyburide	1.22	Moderate
5	0.57	95	140 ± 126	Propranolol	Glyburide	1.61	Moderate
6	0.56	25	157 ± 136	Diltiazem	Amiodarone	1.26	Major
7	0.56	13	122 ± 113	Propranolol	Methyldopa	8.50	Major
8	0.55	470	160 ± 133	Diltiazem	Simvastatin	1.27	Major
9	0.55	5	82 ± 86	Propranolol	Aminophylline	1.06	Major
10	0.55	272	140 ± 114	Digoxin	Spironolactone	0.58	Minor
11	0.53	2	288 ± 213	Phenytoin	Medroxyproges. Ac.	inf	Moderate
12	0.52	91	154 ± 142	Atenolol	Diltiazem	1.19	Major
13	0.51	1	31 ± 0	Phenytoin	Sulfadiazine	0.00	Moderate
14	0.50	90	161 ± 157	Imipramine	Carbamazepine	1.35	Moderate
15	0.50	377	143 ± 138	Fluoxetine	Carbamazepine	0.98	Moderate
16	0.49	226	151 ± 145	Amitriptyline	Carbamazepine	0.99	Moderate
17	0.49	52	118 ± 114	Digoxin	Amiodarone	0.56	Major
18	0.49	1	179 ± 0	Phenytoin	Levonorgestrel	inf	Major
18	0.49	1	179 ± 0	Phenytoin	Ethinyl Estradiol	inf	Major
20	0.49	148	168 ± 160	Haloperidol	Lithium	1.31	Major

Supplementary Table 13: Top 20 known DDI pairs (i, j) by rank of $\tau^{\Phi}_{i,j}$, the normalized co-administration length (1st and 2nd columns, respectively). The number of patients affected by the drug pair, $\lvert U^{\Phi}_{i,j}\rvert$, is shown in column 3. Mean (± s.d.) co-administration length, $\langle\lambda^{u}_{i,j}\rangle$, is shown in column 4 (in days) for each DDI pair (i, j) whose English drug names are shown in columns 5 and 6. The relative gender risk of DDI pair co-administration, $RRI^{F}_{i,j}$, shown in column 7. DDI severity classification, according to *Drugs.com*, shown in column 8; DDIs or drugs not found in *Drugs.com* are labeled as *None* or ***, respectively.

4.1 Interactions per severity

In this section, Supplementary Table 14 shows the number of individual interactions and unique users, both per severity of interaction.

Note that some interactions present in DrugBrank were not found in Drugs.com. These are marked as *None*. The drug *Fenoterol* (brand name *Berotec* in Brazil) was not found in Drugs.com. These interactions were summed separately and are shown with an asterisk (*).

severity s	Φ_s	$\lvert U_s^\Phi \rvert$	$\lvert U_s^\Phi \rvert / \lvert U \rvert$	$\lvert U_s^\Phi \rvert / \lvert \Omega \rvert$
Major	5,968 (22.50%)	5,224	3.94%	1.54%
Moderate	18,335 (69.13%)	12,711	9.58%	3.75%
Minor	542 (02.04%)	528	0.40%	0.16%
None	1,489 (05.61%)	1,314	0.99%	0.39%
*	190 (00.72%)	179	0.13%	0.05%
Total	26,524 (100%)	19,956	-%	-%

Supplementary Table 14: The 2nd column lists the numbers of interactions, Φ_s, per DDI severity class (1st column); percentages of interactions per class are shown in parenthesis. Drugs or interactions identified in *DrugBank* but not present in Drugs.com are tallied as *None*. Interactions for *Berotec* tallied as *. The 3rd column lists the number of patients affected by at least one interaction $|U_s^\Phi|$, per DDI severity. Fourth and fifth columns lists the proportion of patients in each DDI severity class for the *Pronto* system and the entire Blumenau populations, respectively. Notice that the same patient may have been administered DDI of more than one severity type.

4.2 Interactions per gender

In this section, Supplementary Table 15 shows the number of individual interactions and unique users per gender.

gender g	Φ^g	$\lvert U^{\Phi,g} \rvert$	$\lvert U^{\Phi,g} \rvert / \lvert U \rvert$	$\lvert U^{\Phi,g} \rvert / \lvert \Omega \rvert$
Male	8,100 (30.54%)	4,793	3.61%	1.41%
Female	18,424 (69.46%)	10,734	8.09%	3.17%
Total	26,524 (100%)	15,527	11.70%	4.58%

Supplementary Table 15: The 2nd column lists the numbers of interactions, Φ, per gender (1st column); percentages of interactions per gender shown in parenthesis. The 3rd column lists the number of patients affected by at least one interaction per gender, $|U^{\Phi,g}|$. The 4th and 5th columns show the proportion of patients in each gender for the *Pronto* system and entire Blumenau populations, respectively.

4.3 Interactions per age

Supplementary Table 16 shows the number of individual interaction and unique users per age group.

age y	Φ^y	$\lvert U^{\Phi,y}\rvert$	$\lvert U^{\Phi,y}\rvert/\lvert U\rvert$	$\lvert U^{\Phi,y}\rvert/\lvert\Omega\rvert$
00-04	23 (0.09%)	20	0.02%	0.01%
05-09	7 (0.03%)	7	0.01%	0.00%
10-14	29 (0.11%)	25	0.02%	0.01%
15-19	172 (0.65%)	139	0.10%	0.04%
20-24	311 (1.17%)	237	0.18%	0.07%
25-29	433 (1.63%)	301	0.23%	0.09%
30-34	771 (2.91%)	525	0.40%	0.15%
35-39	1,097 (4.14%)	687	0.52%	0.20%
40-44	1,581 (5.96%)	1,023	0.77%	0.30%
45-49	2,332 (8.79%)	1,426	1.07%	0.42%
50-54	3,128 (11.79%)	1,868	1.41%	0.55%
55-59	3,447 (13.00%)	1,956	1.47%	0.58%
60-64	3,508 (13.23%)	2,006	1.51%	0.59%
65-69	3,254 (12.27%)	1,794	1.35%	0.53%
70-74	2,417 (9.11%)	1,311	0.99%	0.39%
75-79	1,978 (7.46%)	1,057	0.80%	0.31%
80-84	1,143 (4.31%)	638	0.48%	0.19%
85-89	620 (2.34%)	349	0.26%	0.10%
90-94	205 (0.77%)	117	0.09%	0.03%
95-99	49 (0.18%)	27	0.02%	0.01%
>99	19 (0.07%)	14	0.01%	0.00%
Total	26,524 (100%)	15,527	11.70%	4.58%

Supplementary Table 16: The 2nd column lists the numbers of interactions, Φ^y, per age range (1st column); percentages of interactions per age range shown in parenthesis. The 3rd column lists the number of patients affected by at least one interaction per age range, $|U^{\Phi,y}|$. The 4th and 5th columns show the proportion of patients in each age range from the *Pronto* system and entire Blumenau populations, respectively.

4.4 Interaction per age and gender

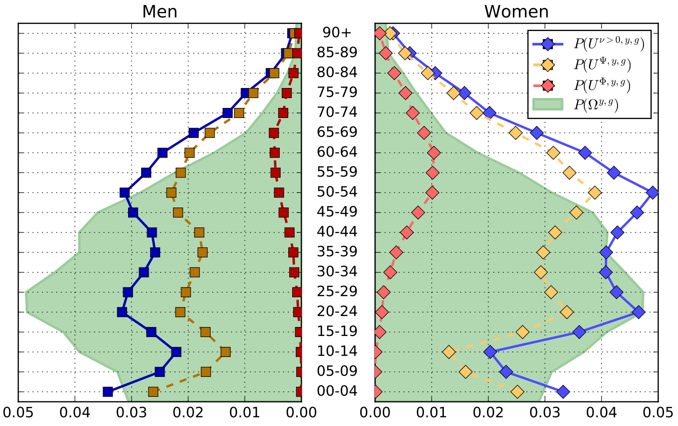

Supplementary Figure 4: The joint probability a patient was dispensed at least one drug $P(U^{\nu>0,y,g})$, had co-administrations $P(U^{\Psi,y,g})$, or had a DDI $P(U^{\Phi,y,g})$, given age range ($[y_1, y_2]$) and gender (g), are shown in blue, orange and red lines, respectively. Values for age group $y \geq 90$ were aggregated for plotting. Population distribution for Blumenau $P(\Omega^{y,g})$ is shown as a green fill. A Kolmogorov-Smirnov test cannot reject the hypothesis that both the female and male distribution of patients with at least one co-administration known to be DDI ($U^{\Phi,y,g}$) are drawn from the same underlying continuous distribution ($KS = .3810$, p-value $= .0706$).

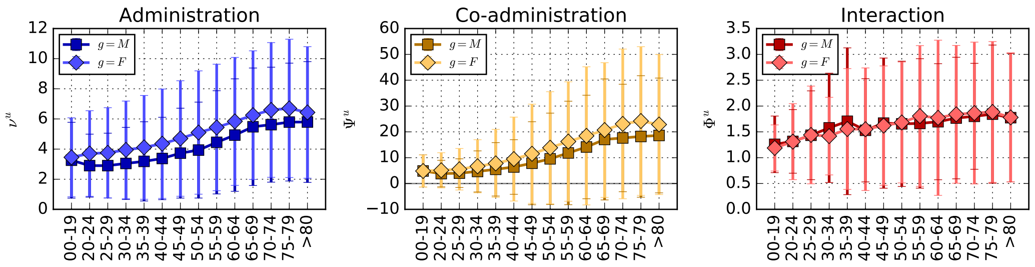

Supplementary Figure 5: Left. Mean number of drugs dispensed (ν^u) to patients in each age group. **Middle.** Mean number of drug pairs co-administered (Ψ^u) by patients in each age group. **Right.** Mean number of drug pairs known to be a DDI (Φ^u) co-administered by patients in each age group. Numbers for male and female patients shown in lighter and darker colors, respectively. In all plots vertical bars denote the standard deviation.

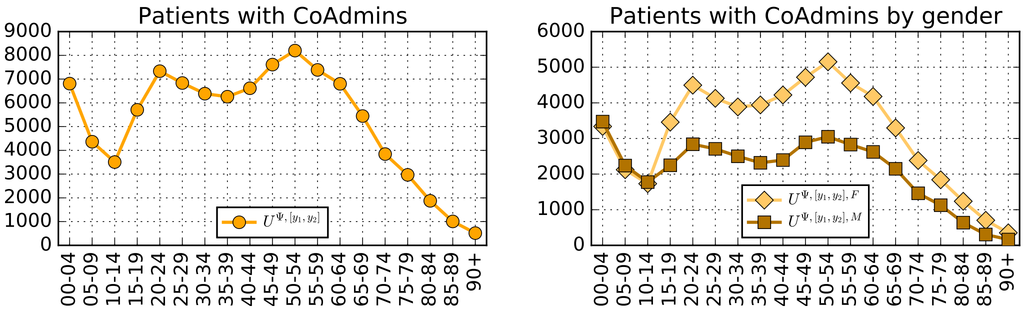

Supplementary Figure 6: Left. Absolute number of patients with at least one co-administration per age group, $|U^{\Psi,[y_1,y_2]}|$. **Right.** Absolute number of patients with at least one co-administration per age group and gender, $|U^{\Psi,[y_1,y_2],g}|$.

Supplementary Note 5 Risk and Relative Risk measures

Risk and Relative Risk measures are computed based on the number of patients in specific groups. Here we detail the computation of all measures used throughout the main manuscript.

The relative risk of co-administration and interaction for women, are computed as

$$RRC^{\mathrm{F}} = \frac{P(\Psi^u > 0 \,|\, u \in U^{\mathrm{F}})}{P(\Psi^u > 0 \,|\, u \in U^{\mathrm{M}})} = \frac{|U^{\Psi,\mathrm{F}}|\,/|U^{\mathrm{F}}|}{|U^{\Psi,\mathrm{M}}|\,/|U^{\mathrm{M}}|};\; RRI^{\mathrm{F}} = \frac{P(\Phi^u > 0 \,|\, u \in U^{\mathrm{F}})}{P(\Phi^u > 0 \,|\, u \in U^{\mathrm{M}})} = \frac{|U^{\Phi,\mathrm{F}}|\,/|U^{\mathrm{F}}|}{|U^{\Phi,\mathrm{M}}|\,/|U^{\mathrm{M}}|}. \tag{10}$$

Similarly, $RRC^M = 1/RRC^F$ and $RRI^M = 1/RRI^F$. In the main manuscript we mentioned the computation of RRI^F without contraceptive drugs. The drug removed in this computation were *Ethinyl Estradiol*, *Estradiol*, *Norethisterone*, *Levonorgestrel* and *Estrogens Conjugated*.

The relative risk of an interaction at a certain DDI severity level, $s \in \{\text{major}, \text{moderate}, \text{minor}, \text{none}, *\}$, given gender, is computed as

$$RRI_s^F = \frac{P(\Phi_s^u > 0 \,|\, u \in U^{\mathrm{F}})}{P(\Phi_s^u > 0 \,|\, u \in U^{\mathrm{M}})} = \frac{|U_s^{\Phi,\mathrm{F}}|/|U^{\mathrm{F}}|}{|U_s^{\Phi,\mathrm{M}}|\;|U^{\mathrm{M}}|} \quad . \tag{11}$$

The relative risk of an interaction between two drugs, given gender, is computed as

$$RRI_{i,j}^F = \frac{P(\Phi_{i,j}^u > 0 \,|\, u \in U^{\mathrm{F}})}{P(\Phi_{i,j}^u > 0 \,|\, u \in U^{\mathrm{M}})} = \frac{|U_{i,j}^{\Phi,\mathrm{F}}|/|U^{\mathrm{F}}|}{|U_{i,j}^{\Phi,\mathrm{M}}|/|U^M|}. \tag{12}$$

The relative risk of co-administration and interaction, given number of dispensed drugs, are computed as

$$RRC^{\nu=x} = \frac{P(\Psi^u \mid u \in U^{\nu=x})}{P(\Psi^y \mid u \in U^{\nu=2})} = \frac{|U^{\Psi,\nu=x}|/|U^{\nu=x}|}{|U^{\Psi,\nu=2}|/|U^{\nu=2}|};\ RRI^{\nu=x} = \frac{P(\Phi^u \mid u \in U^{\nu=x})}{P(\Phi^u \mid u \in U^{\nu=2})} = \frac{|U^{\Phi,\nu=x}|/|U^{\nu=x}|}{|U^{\Phi,\nu=2}|/|U^{\nu=2}|}. \tag{13}$$

The risk of co-administration and interaction, given age group, are computed as

$$RC^{[y_1,y_2]} = \frac{P(\Psi^u > 0 \mid u \in U^{[y_1,y_2]})}{P(\nu^u \geq 2 \mid u \in U^{[y_1,y_2]})} = \frac{|U^{\Psi,[y_1,y_2]}|}{|U^{\nu\geq 2,[y_1,y_2]}|};\ RI^{[y_1,y_2]} = \frac{P(\Phi^u > 0 \mid u \in U^{[y_1,y_2]})}{P(\Psi^u > 0 \mid u \in U^{[y_1,y_2]})} = \frac{|U^{\Psi,[y_1,y_2]}|}{|U^{\Phi,[y_1,y_2]}|}. \tag{14}$$

Note $RC^{[y_1,y_2]}$ and $RI^{[y_1,y_2]}$ can be also interpreted as probabilities. Similarly, we also compute the risk of co-administration and interaction, per age group and gender as

$$RC^{g,[y_1,y_2]} = \frac{P(\Psi^u > 0 \mid u \in U^{g,[y_1,y_2]})}{P(\nu^u \geq 2 \mid u \in U^{g,[y_1,y_2]})} = \frac{|U^{\Psi,g,[y_1,y_2]}|}{|U^{\nu\geq 2,g,[y_1,y_2]}|};\ RI^{g,[y_1,y_2]} = \frac{P(\Phi^u > 0 \mid u \in U^{g,[y_1,y_2]})}{P(\Psi^u > 0 \mid u \in U^{g,[y_1,y_2]})} = \frac{|U^{\Psi,g,[y_1,y_2]}|}{|U^{\Phi,g,[y_1,y_2]}|}. \tag{15}$$

5.1 Relative Risk per gender

g	$\lvert U^g\rvert$	$\lvert U^{\nu\geq 2}\rvert$	$\lvert U^{\Psi,g}\rvert$	$\lvert U^{\Phi,g}\rvert$	RRC^F	RRI^F
Male	55,032	41,922	39,723	4,793	1.0000	1.0000
Female	77,690	62,889	59,738	10,734	1.0653	1.5864

Supplementary Table 17: Absolute number of patients and relative risk measures per gender (g, 1st column). Columns 2 through 5 lists, per gender, absolute numbers of: patients ($|U^g|$), patients with at least 2 administrations ($|U^{\nu\geq 2}|$), patients with at least one co-administration ($|U^{\Psi,g}|$), and patients with at least one known DDI co-administration ($|U^{\Phi,g}|$). Relative Risk for women for both co-administration (RRC^F) and known DDI co-administration (RRI^F) are listed in columns 6 and 7, respectively.

rankp(RRI, U^{F})	$\lvert U^{\Phi,M}\rvert$	$\lvert U^{\Phi,F}\rvert$	$\langle \lambda^u_{i,j} \rangle$	i	j	$RRI^F_{i,j}$	severity
1 (1,49)	0	30	10 ± 3	Clavulanate	Ethinyl Estradiol	inf	None
2 (1,51)	0	27	8 ± 7	Prednisone	Estradiol	inf	Moderate
2 (1,51)	0	27	11 ± 6	Doxycycline	Ethinyl Estradiol	inf	Moderate
4 (1,57)	0	22	19 ± 28	Estrogens Conj.	Prednisone	inf	Moderate
5 (1,71)	0	13	35 ± 26	Carbamazepine	Ethinyl Estradiol	inf	Major
5 (1,71)	0	13	35 ± 26	Levonorgestrel	Carbamazepine	inf	Major
7 (76,1)	1204	3874	102 ± 95	Omeprazole	Clonazepam	2.28	Moderate
8 (1,83)	0	9	72 ± 128	Carbamazepine	Norethisterone	inf	Major
9 (1,89)	0	7	11 ± 4	Doxycycline	Iron (II) Sulfate	inf	Moderate
10 (1,94)	0	5	162 ± 120	Medroxyproges. Ac.	Phenobarbital	inf	Moderate
10 (1,94)	0	5	10 ± 6	Prednisolone	Ethinyl Estradiol	inf	Moderate
12 (1,98)	0	4	40 ± 29	Levonorgestrel	Phenobarbital	inf	Major
12 (1,98)	0	4	10 ± 2	Estrogens Conj.	Prednisolone	inf	Moderate
14 (1,102)	0	3	97 ± 94	Estrogens Conj.	Phenobarbital	inf	Moderate
14 (1,102)	0	3	40 ± 57	Methyldopa	Levodopa	inf	Minor
14 (1,102)	0	3	45 ± 43	Medroxyproges. Ac.	Warfarin	inf	None
14 (1,102)	0	3	40 ± 19	Digoxin	Verapamil	inf	Moderate
14 (1,102)	0	3	53 ± 16	Ethinyl Estradiol	Phenobarbital	inf	Major
14 (1,102)	0	3	136 ± 117	Ethinyl Estradiol	Aminophylline	inf	Moderate
20 (1,111)	0	2	23 ± 11	Doxycycline	Phenobarbital	inf	Moderate
20 (1,111)	0	2	79 ± 30	Norethisterone	Phenobarbital	inf	Major
20 (1,111)	0	2	288 ± 213	Phenytoin	Medroxyproges. Ac.	inf	Moderate
20 (1,111)	0	2	62 ± 53	Phenytoin	Norethisterone	inf	Major
20 (1,111)	0	2	62 ± 53	Phenytoin	Estradiol	inf	Moderate
20 (1,111)	0	2	79 ± 30	Estradiol	Phenobarbital	inf	Moderate
20 (1,111)	0	2	2 ± 0	Timolol	Fenoterol	inf	*
20 (1,111)	0	2	31 ± 0	Timolol	Aminophylline	inf	Major
20 (1,111)	0	2	6 ± 1	Estradiol	Prednisolone	inf	Moderate
29 (1,124)	0	1	117 ± 0	Haloperidol	Methyldopa	inf	Moderate
29 (1,124)	0	1	2 ± 0	Atenolol	Epinephrine	inf	Moderate
29 (1,124)	0	1	12 ± 0	Timolol	Methyldopa	inf	Major
29 (1,124)	0	1	29 ± 0	Phenytoin	Estrogens Conj.	inf	Moderate
29 (1,124)	0	1	2 ± 0	Erythromycin	Diazepam	inf	Moderate
29 (1,124)	0	1	4 ± 0	Erythromycin	Aminophylline	inf	Moderate
29 (1,124)	0	1	179 ± 0	Phenytoin	Ethinyl Estradiol	inf	Major
29 (1,124)	0	1	179 ± 0	Phenytoin	Levonorgestrel	inf	Major
29 (1,124)	0	1	15 ± 0	Phenytoin	Doxycycline	inf	Moderate
29 (1,124)	0	1	9 ± 0	Erythromycin	Fluoxetine	inf	Moderate
39 (104,2)	706	1411	53 ± 74	ASA	Ibuprofen	1.42	Major
40 (59,4)	198	992	127 ± 127	Amitriptyline	Fluoxetine	3.55	Major
41 (83,3)	400	1060	54 ± 77	Atenolol	Ibuprofen	1.88	Moderate
42 (75,5)	189	703	56 ± 61	Fluconazole	Simvastatin	2.63	Major
43 (62,7)	108	519	46 ± 54	Fluconazole	Clonazepam	3.40	None
44 (61,9)	86	415	44 ± 62	Propranolol	Ibuprofen	3.42	Moderate
45 (52,12)	46	309	86 ± 84	Fluoxetine	Propranolol	4.76	Moderate
46 (38,17)	1	178	10 ± 6	Ethinyl Estradiol	Amoxicillin	126.09	Moderate
47 (117,6)	369	630	87 ± 86	Omeprazole	Diazepam	1.21	Moderate
48 (53,14)	38	246	66 ± 57	Levothyroxine	Iron (II) Sulfate	4.59	Moderate
49 (81,10)	134	366	52 ± 75	Furosemide	Ibuprofen	1.93	Moderate
49 (54,15)	35	210	42 ± 40	Fluconazole	Amitriptyline	4.25	Moderate
51 (110,8)	265	487	30 ± 50	ASA	Dexamethasone	1.30	Moderate
52 (64,16)	48	209	123 ± 130	Imipramine	Fluoxetine	3.08	Major
53 (47,23)	12	104	9 ± 7	Norfloxacin	Iron (II) Sulfate	6.14	Moderate
54 (41,27)	7	95	4 ± 4	Diclofenac	Alendronate	9.61	Moderate
55 (39,29)	1	83	15 ± 26	Prednisone	Ethinyl Estradiol	58.79	Moderate
56 (115,11)	197	338	152 ± 132	Atenolol	Glyburide	1.22	Moderate
57 (40,35)	2	61	33 ± 28	Methyldopa	Iron (II) Sulfate	21.60	Moderate
58 (71,20)	31	124	68 ± 80	Amitriptyline	Salbutamol	2.83	Moderate
59 (112,13)	168	302	160 ± 133	Diltiazem	Simvastatin	1.27	Major
60 (84,18)	63	159	148 ± 139	Fluoxetine	Lithium	1.79	Major

Supplementary Table 18: Top 60 known DDI pairs (i, j) most imbalanced for *females*, sorted by rank product (1st column; individual rank in parenthesis) of $RRI^F_{i,j}$, the relative gender risk of DDI pair co-administration, and $\lvert U^{\Phi,F}\rvert$, the number of women affected by the DDI (7nd and 3rd columns, respectively). The number of men ($\lvert U^{\Phi,M}\rvert$) affected is shown in column 2. Mean (± s.d.) co-administration length, $\langle \lambda^u_{i,j} \rangle$, is shown in column 4 (in days) for each DDI pair (i, j) whose English drug names are shown in columns 5 and 6. DDI severity classification, according to *Drugs.com*, shown in column 8; DDIs or drugs not found in *Drugs.com* are labeled as *None* or ***, respectively.

rankp(RRI, U^{M})	$\lvert U^{\Phi,M}\rvert$	$\lvert U^{\Phi,F}\rvert$	$\langle\lambda^{u}_{i,j}\rangle$	i	j	$RRI^{M}_{i,j}$	severity
1 (1,32)	4	0	72 ± 61	Atenolol	Verapamil	inf	Major
2 (1,38)	3	0	185 ± 98	Phenytoin	Levodopa	inf	Moderate
3 (20,2)	280	244	243 ± 188	Haloperidol	Biperiden	1.62	Moderate
3 (40,1)	553	696	141 ± 124	ASA	Glyburide	1.12	Moderate
5 (1,43)	1	0	124 ± 0	Carbamazepine	Verapamil	inf	Major
5 (1,43)	1	0	6 ± 0	Erythromycin	Carbamazepine	inf	Major
5 (1,43)	1	0	31 ± 0	Phenytoin	Sulfadiazine	inf	Moderate
8 (18,5)	207	178	155 ± 125	Digoxin	Furosemide	1.64	Moderate
9 (13,9)	99	74	109 ± 96	Digoxin	Carvedilol	1.89	Moderate
10 (8,15)	37	10	135 ± 109	Allopurinol	Warfarin	5.22	Moderate
11 (41,3)	262	347	48 ± 93	Prednisone	ASA	1.07	Moderate
12 (33,4)	236	260	103 ± 87	ASA	Gliclazide	1.28	None
13 (17,8)	149	123	140 ± 114	Digoxin	Spironolactone	1.71	Minor
14 (6,24)	10	1	57 ± 46	Methylphenidate	Carbamazepine	14.12	None
15 (21,9)	99	87	142 ± 156	Haloperidol	Carbamazepine	1.61	Moderate
16 (7,32)	4	1	107 ± 123	Phenytoin	Amiodarone	5.65	Moderate
17 (12,22)	16	11	10 ± 6	Phenytoin	Ciprofloxacin	2.05	Moderate
18 (16,17)	29	23	118 ± 114	Digoxin	Amiodarone	1.78	Major
19 (46,6)	158	219	143 ± 138	Fluoxetine	Carbamazepine	1.02	Moderate
20 (11,26)	9	6	20 ± 17	Carvedilol	Fenoterol	2.12	*
21 (42,7)	156	208	110 ± 106	Carbamazepine	Simvastatin	1.06	Moderate
22 (9,38)	3	1	62 ± 44	Phenobarbital	Aminophylline	4.24	Moderate
23 (19,20)	23	20	144 ± 153	Phenytoin	Diazepam	1.62	Moderate
24 (15,26)	9	7	94 ± 83	Phenytoin	Furosemide	1.82	Minor
25 (14,28)	8	6	5 ± 3	Doxycycline	Amoxicillin	1.88	Moderate
26 (34,12)	70	79	115 ± 109	Phenytoin	Omeprazole	1.25	Moderate
27 (10,41)	2	1	9 ± 4	Phenytoin	Prednisolone	2.82	Moderate
28 (22,21)	20	19	82 ± 64	Gliclazide	Carvedilol	1.49	None
29 (36,13)	55	68	31 ± 43	Ibuprofen	Carvedilol	1.14	Moderate
30 (30,16)	35	36	169 ± 151	Phenytoin	Fluoxetine	1.37	Moderate
31 (48,11)	94	132	151 ± 145	Amitriptyline	Carbamazepine	1.01	Moderate
32 (32,17)	29	31	49 ± 95	Prednisone	Warfarin	1.32	Moderate
33 (31,19)	28	29	96 ± 96	Glyburide	Carvedilol	1.36	Moderate
34 (45,14)	50	67	126 ± 127	Digoxin	Hydrochlorothiazide	1.05	Moderate
35 (23,30)	7	7	9 ± 7	Furosemide	Gentamicin	1.41	Major
36 (24,32)	4	4	82 ± 54	Carbamazepine	Aminophylline	1.41	Moderate
36 (24,32)	4	4	67 ± 36	Diltiazem	Carbamazepine	1.41	Major
38 (35,23)	11	13	3 ± 2	Diclofenac	Warfarin	1.19	Major
39 (24,41)	2	2	4 ± 3	Doxycycline	Clavulanate	1.41	None
40 (24,43)	1	1	102 ± 110	Propranolol	Verapamil	1.41	Major
40 (24,43)	1	1	274 ± 218	Digoxin	Propylthiouracil	1.41	Moderate
40 (24,43)	1	1	2 ± 0	Phenytoin	Hydrocortisone	1.41	Moderate
43 (37,28)	8	10	33 ± 44	Fluconazole	Warfarin	1.13	Major
44 (47,24)	10	14	100 ± 85	Carbamazepine	Warfarin	1.01	Moderate
45 (37,32)	4	5	51 ± 91	Timolol	Salbutamol	1.13	Major
45 (37,32)	4	5	7 ± 1	Prednisolone	Phenobarbital	1.13	Moderate
47 (42,31)	6	8	88 ± 71	Phenytoin	Warfarin	1.06	Moderate
48 (42,38)	3	4	62 ± 97	Phenytoin	Trimethoprim	1.06	Moderate

Supplementary Table 19: All 48 known DDI pairs (i, j) most imbalaced for *males*, sorted by rank product (1st column; individual rank in parenthesis) of $RRI^{M}_{i,j}$, the relative gender risk of DDI pair co-administration, and $\lvert U^{\Phi,M}\rvert$, the number of men affected by the DDI (7nd and 2rd columns, respectively). The number of women ($\lvert U^{\Phi,F}\rvert$) affected is shown in column 3. Mean (± s.d.) co-administration length, $\langle\lambda^{u}_{i,j}\rangle$, is shown in column 4 (in days) for each DDI pair (i, j) whose English drug names are shown in columns 5 and 6. DDI severity classification, according to *Drugs.com*, shown in column 8; DDIs or drugs not found in *Drugs.com* are labeled as *None* or ***, respectively.

$RRI^{g}_{i,j} \geq x$	$\lvert D^{\Phi,F}\rvert$	$\lvert D^{\Phi,M}\rvert$	Φ^{F}	Φ^{M}	Φ^{F}_{major}	Φ^{M}_{major}
1	68	46	133	48	31	10
2	56	17	80	12	21	3
3	49	13	65	9	16	3
4	45	13	58	9	13	3
5	38	11	49	8	13	3
6	36	8	47	6	13	3
7	35	8	45	6	12	3
8	32	8	42	6	12	3
9	31	8	41	6	11	3
10	29	8	40	6	11	3

Supplementary Table 20: Number and proportions of drugs and interactions at increasing level of relative gender risk of DDI pair co-administration, $RRI^{g}_{i,j} > x$ (1st column). Number of drugs by gender, is shown in columns 2 and 3. Number of drug pairs known to be a DDI, by gender, is shown in columns 4 and 5. Number of drug pairs, known to be a *major* DDI, by gender, is shown in columns 6 and 7. See also Supplementary Table 21.

$RRI^g_{i,j} \geq x$	$\lvert U^{\Phi,F}\rvert$	$\lvert U^{\Phi,M}\rvert$	$\lvert U^{\Phi,F}_{major}\rvert$	$\lvert U^{\Phi,M}_{major}\rvert$	$\frac{\lvert U^{\Phi,F}\rvert}{\lvert U^F\rvert}$	$\frac{\lvert U^{\Phi,M}\rvert}{\lvert U^M\rvert}$	$\frac{\lvert U^{\Phi,F}_{major}\rvert}{\lvert U^F\rvert}$	$\frac{\lvert U^{\Phi,M}_{major}\rvert}{\lvert U^M\rvert}$
1	9,836	2,010	3,747	69	12.66%	03.65%	04.82%	00.13%
2	7,089	91	2,060	6	09.12%	00.17%	02.65%	00.01%
3	3,327	64	1,255	6	04.28%	00.12%	01.62%	00.01%
4	1,589	64	73	6	02.05%	00.12%	00.09%	00.01%
5	775	61	73	6	01.00%	00.11%	00.09%	00.01%
6	744	20	73	6	00.96%	00.04%	00.09%	00.01%
7	615	20	45	6	00.79%	00.04%	00.06%	00.01%
8	547	20	45	6	00.70%	00.04%	00.06%	00.01%
9	536	20	33	6	00.69%	00.04%	00.04%	00.01%
10	441	20	33	6	00.57%	00.04%	00.04%	00.01%

Supplementary Table 21: Number and proportions of affected patients at increasing level of relative gender risk of DDI pair co-administration, $RRI^g_{i,j} > x$ (1st column). Number of patients by gender, is shown in columns 2 and 3. Number of patients by gender and major DDI, is shown in columns 4 and 5. The relative proportion of affected patients in relation to the Pronto population is shown in columns 6 through 9. See also Supplementary Table 20.

5.2 Relative Risk per severity

severity s	$\|U_s^{\Phi,M}\|$	$\|U_s^{\Psi,F}\|$	RRI_s^F
Major	1,433	3791	1.8739
Moderate	3,951	8760	1.5705
Minor	247	281	0.8059
None	409	905	1.5674
*	39	140	2.5428

Supplementary Table 22: Absolute number of patients and relative risk measures per gender (g) and severity score (s, 1st column). Columns 2 and 3 lists absolute number of males ($g = M$) and females ($g = F$) affected by at least one DDI for each severity score, respectively. Column 4 lists the relative risk of interaction given a specific severity score and gender. DDIs or drugs not found in *Drugs.com* are labeled as *None* or *, respectively Notice that the same patient may have been administered DDI of more than one severity type.

5.3 Risk Measures per age

$[y_1, y_2]$	$\|U^{[y_1,y_2]}\|$	$\|U^{\nu\geq 2,[y_1,y_2]}\|$	$\|U^{\Psi,[y_1,y_2]}\|$	$\|U^{\Phi,[y_1,y_2]}\|$	$RC^{[y_1,y_2]}$	$RI^{[y_1,y_2]}$
00-04	8,946	7,195	6,810	20	0.9465	0.0029
05-09	6,390	4,688	4,362	7	0.9305	0.0016
10-14	5,631	3,794	3,507	25	0.9244	0.0071
15-19	8,305	6,094	5,705	139	0.9362	0.0244
20-24	10,382	7,819	7,334	237	0.9380	0.0323
25-29	9,725	7,305	6,835	301	0.9357	0.0440
30-34	9,100	6,787	6,386	525	0.9409	0.0822
35-39	8,844	6,696	6,259	687	0.9347	0.1098
40-44	9,184	7,043	6,615	1,023	0.9392	0.1546
45-49	10,085	8,039	7,610	1,426	0.9466	0.1874
50-54	10,650	8,617	8,200	1,868	0.9516	0.2278
55-59	9,236	7,686	7,386	1,956	0.9610	0.2648
60-64	8,179	7,049	6,801	2,006	0.9648	0.2950
65-69	6,315	5,572	5,444	1,794	0.9770	0.3295
70-74	4,412	3,916	3,843	1,311	0.9814	0.3411
75-79	3,398	3,042	2,968	1,057	0.9757	0.3561
80-84	2,129	1,909	1,874	638	0.9817	0.3404
85-89	1,174	1,029	1,007	349	0.9786	0.3466
90+	637	531	515	158	0.9699	0.3068

Supplementary Table 23: Absolute number of patients and risk measures per age range ($[y_1, y_2]$, 1st column). Columns 2 through 5 lists, per age range, absolute numbers of: patients ($|U^{[y_1,y_2]}|$), patients with at least 2 drug administrations ($|U^{\nu\geq 2,[y_1,y_2]}|$), patients with at least one co-administration ($|U^{\Psi,[y_1,y_2]}|$), and patients with at least one known DDI co-administration ($|U^{\Phi,[y_1,y_2]}|$). Per age range risk for both co-administration ($RC^{[y_1,y_2]}$) and known DDI co-administration ($RI^{[y_1,y_2]}$) are listed in columns 6 and 7, respectively.

$[y_1, y_2]$	$\lvert U^{M,[y_1,y_2]}\rvert$	$\lvert U^{\nu\geq 2,M,[y_1,y_2]}\rvert$	$\lvert U^{\Psi,M,[y_1,y_2]}\rvert$	$\lvert U^{\Phi,M,[y_1,y_2]}\rvert$	$RC^{M,[y_1,y_2]}$	$RI^{M,[y_1,y_2]}$
00-04	4,537	3,664	3,473	8	0.9479	0.0023
05-09	3,319	2,416	2,239	3	0.9267	0.0013
10-14	2,932	1,926	1,776	14	0.9221	0.0079
15-19	3,518	2,390	2,247	33	0.9402	0.0147
20-24	4,204	3,020	2,838	76	0.9397	0.0268
25-29	4,066	2,890	2,708	99	0.9370	0.0366
30-34	3,692	2,641	2,500	1,68	0.9466	0.0672
35-39	3,428	2,488	2,317	1,90	0.9313	0.0820
40-44	3,504	2,559	2,394	2,79	0.9355	0.1165
45-49	3,945	3,043	2,892	4,17	0.9504	0.1442
50-54	4,142	3,219	3,048	5,25	0.9469	0.1722
55-59	3,638	2,953	2,829	6,06	0.9580	0.2142
60-64	3,257	2,731	2,622	6,26	0.9601	0.2387
65-69	2,525	2,197	2,148	6,45	0.9777	0.3003
70-74	1,729	1,494	1,461	4,27	0.9779	0.2923
75-79	1,303	1,162	1,127	3,44	0.9699	0.3052
80-84	718	649	637	1,86	0.9815	0.2920
85-89	361	312	304	98	0.9744	0.3224
90+	214	168	163	49	0.9702	0.3006

Supplementary Table 24: Absolute number of *male* patients and risk measures per age range ($[y_1, y_2]$, 1st column). Columns 2 through 5 lists, per age range, absolute numbers of: male patients ($|U^y|$), male patients with at least 2 drug administrations ($|U^{\nu\geq 2,M,[y_1,y_2]}|$), male patients with at least one co-administration ($|U^{\Psi,M,[y_1,y_2]}|$), and male patients with at least one known DDI co-administration ($|U^{\Phi,M,[y_1,y_2]}|$). Per age range women risk for both co-administration ($RC^{M,[y_1,y_2]}$) and known DDI co-administration ($RI^{M,[y_1,y_2]}$) are listed in columns 6 and 7, respectively.

$[y_1, y_2]$	$\lvert U^{F,[y_1,y_2]}\rvert$	$\lvert U^{\nu\geq 2,F,[y_1,y_2]}\rvert$	$\lvert U^{\Psi,F,[y_1,y_2]}\rvert$	$\lvert U^{\Phi,F,[y_1,y_2]}\rvert$	$RC^{F,[y_1,y_2]}$	$RI^{F,[y_1,y_2]}$
00-04	4,409	3,531	3,337	12	0.9451	0.0036
05-09	3,071	2,272	2,123	4	0.9344	0.0019
10-14	2,699	1,868	1,731	11	0.9267	0.0064
15-19	4,787	3,704	3,458	106	0.9336	0.0307
20-24	6,178	4,799	4,496	161	0.9369	0.0358
25-29	5,659	4,415	4,127	202	0.9348	0.0489
30-34	5,408	4,146	3,886	357	0.9373	0.0919
35-39	5,416	4,208	3,942	497	0.9368	0.1261
40-44	5,680	4,484	4,221	744	0.9413	0.1763
45-49	6,140	4,996	4,718	1,009	0.9444	0.2139
50-54	6,508	5,398	5,152	1,343	0.9544	0.2607
55-59	5,598	4,733	4,557	1,350	0.9628	0.2962
60-64	4,922	4,318	4,179	1,380	0.9678	0.3302
65-69	3,790	3,375	3,296	1,149	0.9766	0.3486
70-74	2,683	2,422	2,382	884	0.9835	0.3711
75-79	2,095	1,880	1,841	713	0.9793	0.3873
80-84	1,411	1,260	1,237	452	0.9817	0.3654
85-89	813	717	703	251	0.9805	0.3570
90+	423	363	352	109	0.9697	0.3097

Supplementary Table 25: Absolute number of *female* patients and risk measures per age range ($[y_1, y_2]$, 1st column). Columns 2 through 5 lists, per age range, absolute numbers of: female patients ($|U^y|$), female patients with at least 2 drug administrations ($|U^{\nu\geq 2,F,[y_1,y_2]}|$), female patients with at least one co-administration ($|U^{\Psi,F,[y_1,y_2]}|$), and female patients with at least one known DDI co-administration ($|U^{\Phi,F,[y_1,y_2]}|$). Per age range women risk for both co-administration ($RC^{F,[y_1,y_2]}$) and known DDI co-administration ($RI^{F,[y_1,y_2]}$) are listed in columns 6 and 7, respectively.

5.4 Risk Ratios per number of drug

# of drugs ν	$\lvert U^{\nu}\rvert$	$\lvert U^{\Psi,\nu}\rvert$	$\lvert U^{\Phi,\nu}\rvert$	RRC^{ν}	RRI^{ν}
1	27,911	-	-	-	-
2	25,032	20,517	283	1.0	1.0
3	19,163	18,468	677	1.1758	3.1249
4	14,305	14,185	929	1.2098	5.7443
5	11,026	11,010	1,208	1.2183	9.6908
6	8,587	8,583	1,425	1.2195	14.6785
7	6,438	6,438	1,512	1.2201	20.7735
8	4,970	4,970	1,477	1.2201	26.2865
9	3,877	3,877	1,417	1.2201	32.3283
10	2,932	2,932	1,335	1.2201	40.2742
11	2,264	2,264	1,089	1.2201	42.5462
12	1,691	1,691	936	1.2201	48.9600
13	1,214	1,214	754	1.2201	54.9366
14	937	937	641	1.2201	60.5101
15	618	618	413	1.2201	59.1113
16	482	482	368	1.2201	67.5320
17	366	366	285	1.2201	68.8768
18	268	268	218	1.2201	71.9500
19	177	177	142	1.2201	70.9617
20	131	131	105	1.2201	70.8969
>20	333	333	313	1.2201	83.1398

Supplementary Table 26: Absolute number of patients, join probabilities and relative risk per number of distinct drugs dispensed, ν^{u} (1st column). By definition, patients who had only one distinct drug dispensed could not have had any co-administration or interaction. Columns 2 through 4 lists, per distinct drugs dispensed, absolute numbers of: patients ($|U^{\nu}|$), patients with at least one co-administration ($|U^{\Psi,\nu}|$), and patients with at least one known DDI co-administration ($|U^{\Phi,\nu}|$). Per number of distinct drugs dispensed relative risks for both co-administration (RRC^{ν}) and known DDI co-administration (RRI^{ν}) are listed in columns 5 and 6, respectively.

Supplementary Note 6 DDI Networks

For all pair of drugs known to interact we built two different networks, which are weighted versions of Δ. In these networks weights are defined by either $\tau^{\Phi}_{i,j}$ or $|U^{\Phi}_{i,j}|$. In this section we show alternatives plotting schemes, additional subgraphs, and tables containing values used for plotting or inference. We also show a Principal Component Analysis and two clustering methods performed on the networks.

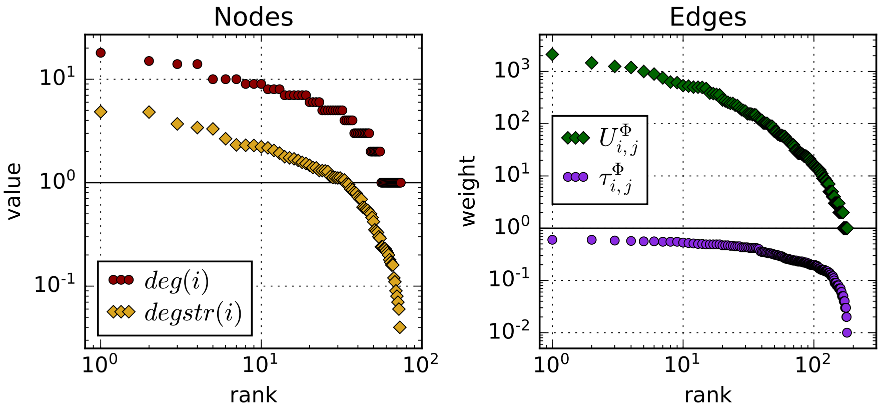

Supplementary Figure 7: Left. Node degree, $deg(i)$, and node degree strength, $degstr(i) = \sum_j \tau^{\Phi}_{i,j}$, of weighted version of network Δ where weights are defined by $\tau^{\Phi}_{i,j}$. **Right.** Edge distribution of weighted version of network Δ, where weights are defined by either $\tau^{\Phi}_{i,j}$ or $|U^{\Phi}_{i,j}|$.

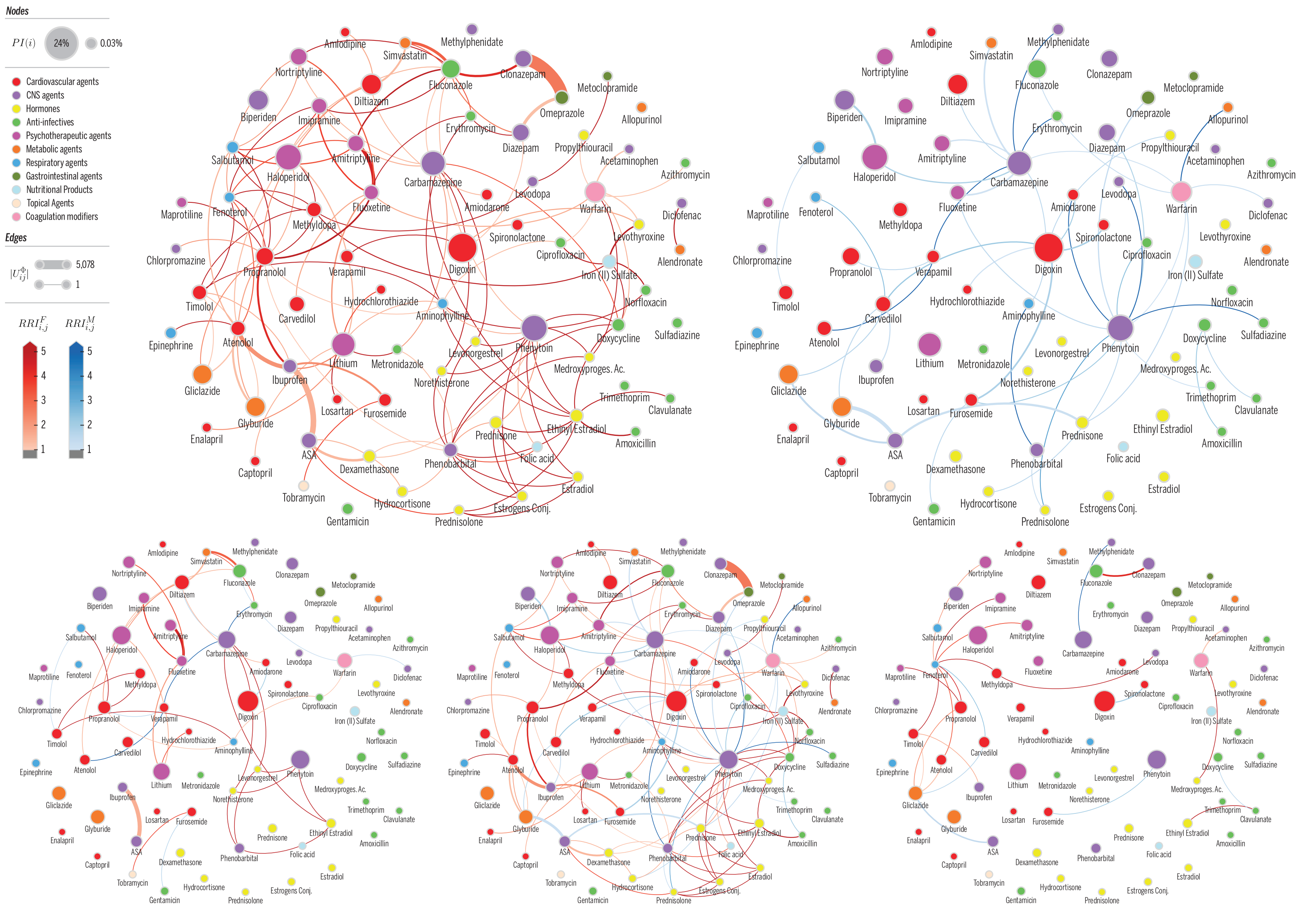

Supplementary Figure 8: A weighted version of network Δ where weights are defined by $|U^{\Phi}_{i,j}|$. **Top left.** Female DDI network. **Top right.** Male DDI network. **Bottom left.** Major DDI network. **Bottom center.** Moderate DDI network. **Bottom right.** Minor DDI network. **Nodes** denote drugs i involved in at least one co-administration known to be a DDI. Node color represents the highest level of primary action class, as retrieved from Drugs.com (see legend) Node size represents the probability of interaction, $PI(i)$, as defined in main text. **Edges weights** are the value of $|U^{\Phi}_{i,j}|$, the number of patients affected by the DDI. **Edge colors** denote $RRI^{g}_{i,j}$, where $g \in \{\text{M}, \text{F}\}$, to identify DDI edges that are higher risk for females (blue) or males (red). Color intensity for $RRI^{g}_{i,j}$ varies in $[1, 5]$; that is, values are clipped at 5.

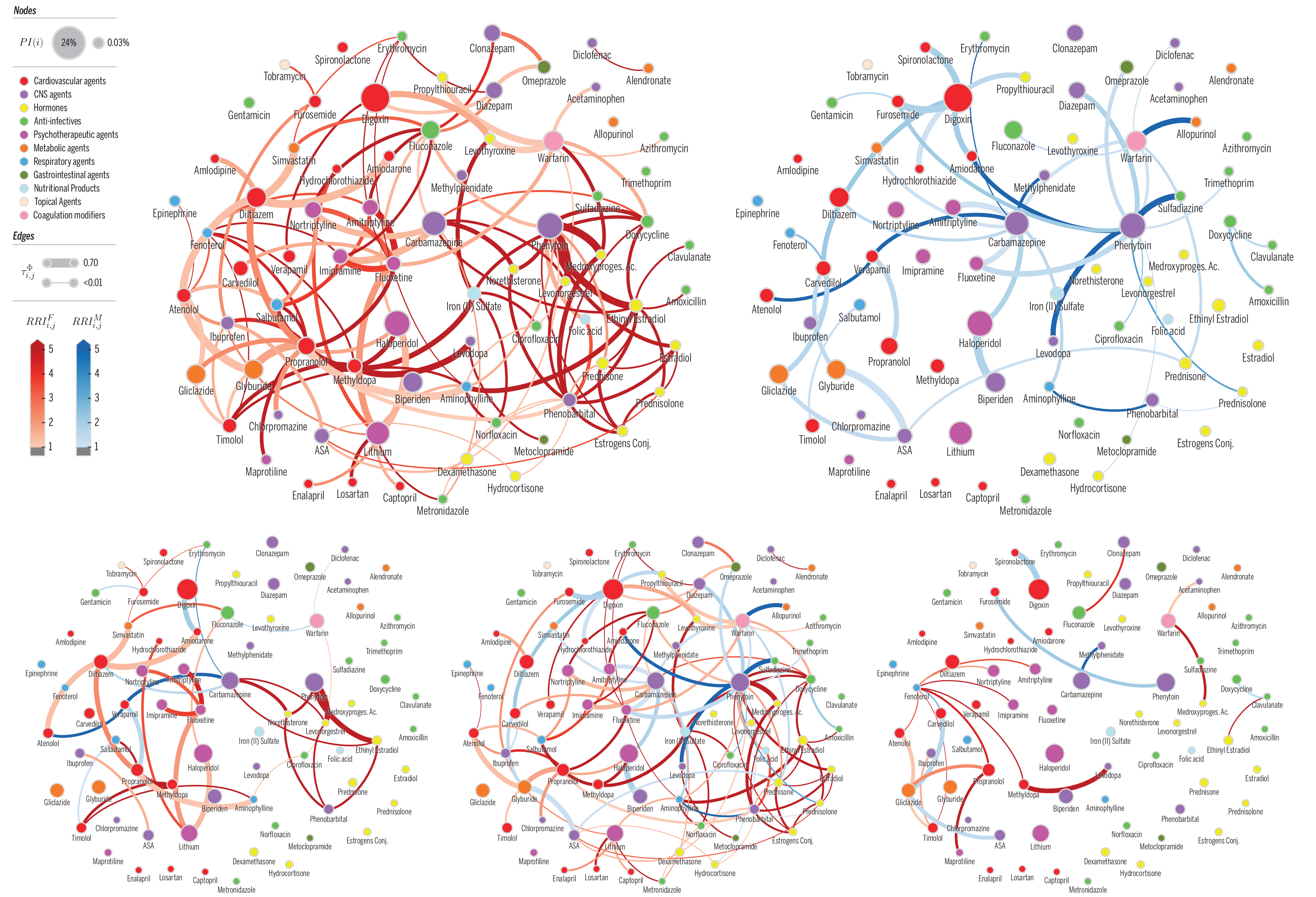

Supplementary Figure 9: A weighted version of network Δ where weights are defined by $\tau^{\Phi}_{i,j}$. **Top left.** Female DDI network. **Top right.** Male DDI network. **Bottom left.** Major DDI network. **Bottom center.** Moderate DDI network. **Bottom right.** Minor DDI network. **Nodes** denote drugs i involved in at least one co-administration known to be a DDI. Node color represents the highest level of primary action class, as retrieved from Drugs.com (see legend) Node size represents the probability of interaction, $PI(i)$, as defined in main text. **Edges weights** are the value of $\tau^{\Phi}_{i,j}$, obtained from Equation 4, a normalized measure of the degree to which a specific DDI is co-prescribed. **Edge colors** denote $RRI^{g}_{i,j}$, where $g \in \{\mathrm{M}, \mathrm{F}\}$, to identify DDI edges that are higher risk for females (blue) or males (red). Color intensity for $RRI^{g}_{i,j}$ varies in $[1, 5]$; that is, values are clipped at 5.

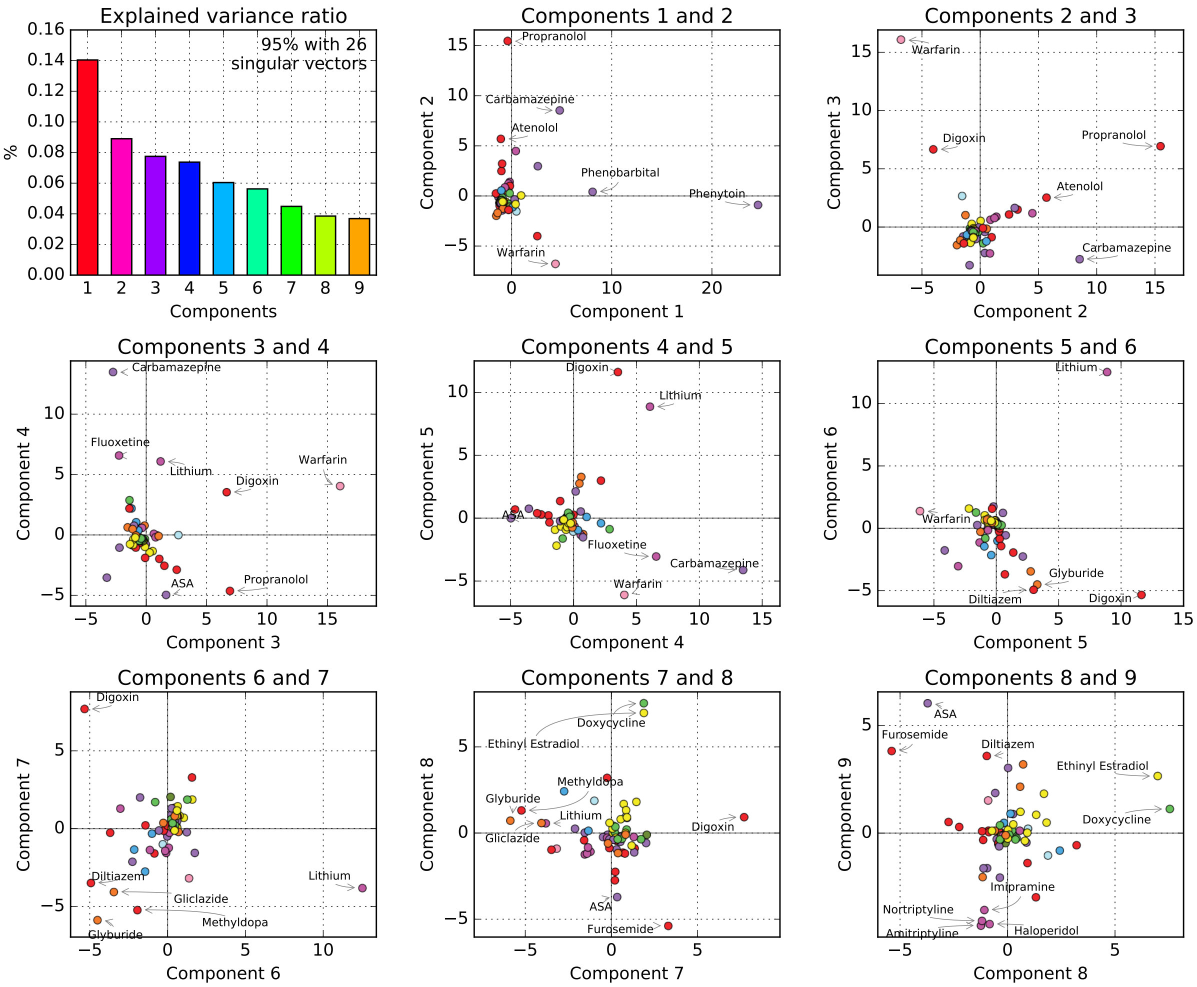

Supplementary Figure 10: Principal Component Analysis (PCA) of network where weights are defined by $\tau^{\Phi}_{i,j}$. **Top Left**. Explained variance ratio for the first 9 principal components. **Additional plots**. Projection of network nodes (drugs) given the respective principal component. Nodes with loading ≥ 2 s.d. in either component are annotated.

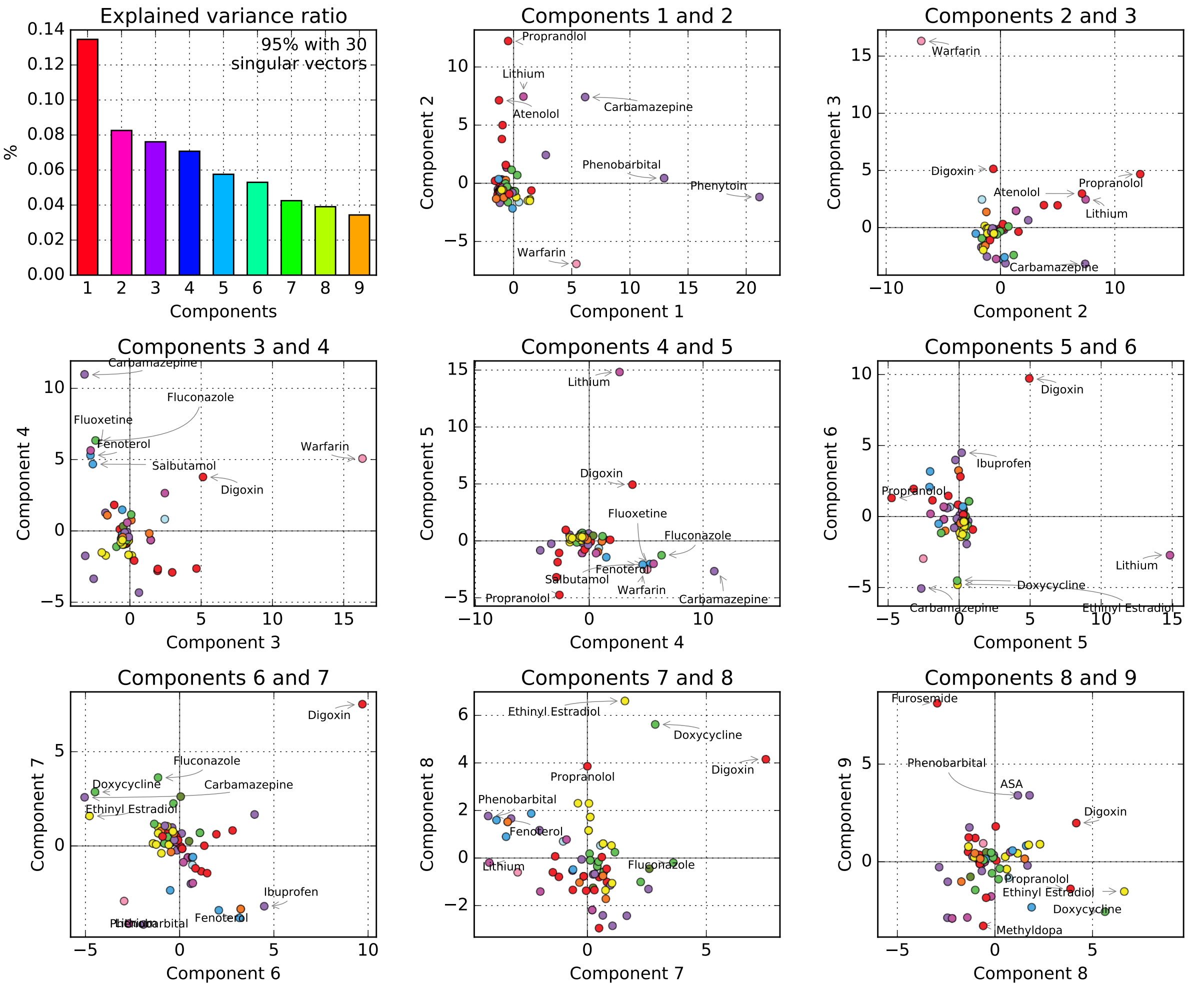

Supplementary Figure 11: Principal Component Analysis (PCA) of network where weights are defined by $|U^{\Phi}_{i,j}|$. **Top Left**. Explained variance ratio for the first 9 principal components. **Additional plots**. Projection of network nodes (drugs) given the respective principal component. Nodes with loading ≥ 2 s.d. in either component are annotated.

i	$deg(i)$	$degstr(i)$	$betweenness(i)$	$PI(i)$	class
Phenytoin	24	6.51	0.30	0.20	CNS agents
Phenobarbital	15	2.17	0.28	0.05	CNS agents
Ethinyl Estradiol	9	1.78	0.03	0.04	Hormones
Doxycycline	8	1.39	0.02	0.04	Anti-infectives
Prednisone	7	0.96	0.02	0.03	Hormones
Prednisolone	6	0.54	0.03	0.00	Hormones
Diazepam	5	1.12	0.05	0.09	CNS agents
Erythromycin	5	0.20	0.18	0.01	Anti-infectives
Estradiol	4	0.57	0.00	0.01	Hormones
Estrogens Conj.	4	0.58	0.00	0.01	Hormones
Norethisterone	3	0.73	0.00	0.00	Hormones
Levonorgestrel	3	0.79	0.00	0.00	Hormones
Medroxyproges. Ac.	3	1.06	0.00	0.00	Hormones
Omeprazole	3	0.85	0.00	0.05	Gastrointestinal agents
Folic acid	2	0.50	0.00	0.00	Nutritional Products
Clonazepam	2	0.42	0.00	0.09	CNS agents
Amoxicillin	2	0.30	0.00	0.00	Anti-infectives
Clavulanate	2	0.23	0.00	0.00	Anti-infectives
Sulfadiazine	1	0.51	0.00	0.01	Anti-infectives
Trimethoprim	1	0.16	0.00	0.00	Anti-infectives
Carbamazepine	18	4.84	0.20	0.18	CNS agents
Fluoxetine	10	3.41	0.02	0.06	Psychotherapeutic agents
Haloperidol	6	2.32	0.03	0.20	Psychotherapeutic agents
Lithium	9	2.05	0.13	0.17	Psychotherapeutic agents
Fluconazole	10	1.74	0.09	0.11	Anti-infectives
Salbutamol	7	1.53	0.00	0.03	Respiratory agents
Amitriptyline	5	1.47	0.00	0.08	Psychotherapeutic agents
Imipramine	5	1.31	0.01	0.07	Psychotherapeutic agents
Nortriptyline	5	1.30	0.00	0.09	Psychotherapeutic agents
Fenoterol	8	0.81	0.13	0.01	Respiratory agents
Biperiden	1	0.70	0.00	0.13	CNS agents
Methylphenidate	1	0.24	0.00	0.02	CNS agents
Losartan	1	0.21	0.00	0.00	Cardiovascular agents
Captopril	1	0.18	0.00	0.00	Cardiovascular agents
Metronidazole	3	0.17	0.16	0.00	Anti-infectives
Enalapril	1	0.16	0.00	0.00	Cardiovascular agents
Methyldopa	7	2.30	0.01	0.06	Cardiovascular agents
Iron (II) Sulfate	5	1.12	0.02	0.04	Nutritional Products
Levodopa	3	0.97	0.03	0.01	CNS agents
Ciprofloxacin	4	0.35	0.21	0.01	Anti-infectives
Norfloxacin	2	0.29	0.00	0.01	Anti-infectives
Metoclopramide	1	0.11	0.00	0.00	Gastrointestinal agents

Supplementary Table 27: Louvain modules of weighted version of network Δ where weights are defined by $\tau^{\Phi}_{i,j}$. Each Louvain module is shown separated by a horizontal line. Drugs nodes (i; 1nd column) and their respective degree, degree strength, and betweenness centrality measure, shown in columns 2, 3, and 4, respectively Column 5 shows the drug probability of interaction, $PI(i)$. Drug class is shown in column 6. Continues on Supplementary Table 28.

i	$deg(i)$	$degstr(i)$	$betweenness(i)$	$PI(i)$	class
Digoxin	9	3.70	0.03	0.24	Cardiovascular agents
Warfarin	14	3.31	0.17	0.13	Coagulation modifiers
Diltiazem	6	2.66	0.03	0.13	Cardiovascular agents
Amiodarone	3	1.40	0.00	0.02	Cardiovascular agents
Furosemide	5	1.31	0.05	0.04	Cardiovascular agents
Levothyroxine	3	1.15	0.00	0.01	Hormones
Simvastatin	4	1.07	0.00	0.02	Metabolic agents
Propylthiouracil	2	0.87	0.00	0.01	Hormones
Hydrochlorothiazide	2	0.69	0.00	0.00	Cardiovascular agents
Spironolactone	1	0.55	0.00	0.02	Cardiovascular agents
Allopurinol	1	0.46	0.00	0.01	Metabolic agents
Amlodipine	1	0.34	0.00	0.00	Cardiovascular agents
Acetaminophen	1	0.22	0.00	0.00	CNS agents
Gentamicin	1	0.12	0.00	0.02	Anti-infectives
Diclofenac	2	0.09	0.03	0.01	CNS agents
Tobramycin	1	0.08	0.00	0.00	Topical Agents
Azithromycin	1	0.07	0.00	0.00	Anti-infectives
Alendronate	1	0.04	0.00	0.01	Metabolic agents
Aminophylline	10	1.93	0.23	0.01	Respiratory agents
Hydrocortisone	3	0.06	0.20	0.01	Hormones
Timolol	7	1.11	0.16	0.06	Cardiovascular agents
Ibuprofen	7	1.28	0.06	0.05	CNS agents
Atenolol	8	2.22	0.05	0.06	Cardiovascular agents
Propranolol	14	4.81	0.06	0.10	Cardiovascular agents
ASA	7	1.57	0.01	0.07	CNS agents
Verapamil	4	1.11	0.01	0.04	Cardiovascular agents
Glyburide	5	2.29	0.00	0.12	Metabolic agents
Carvedilol	6	1.70	0.00	0.07	Cardiovascular agents
Gliclazide	5	1.64	0.00	0.12	Metabolic agents
Chlorpromazine	1	0.33	0.00	0.00	CNS agents
Dexamethasone	3	0.24	0.00	0.03	Hormones
Maprotiline	1	0.23	0.00	0.01	Psychotherapeutic agents
Epinephrine	1	0.0	0.0	0.02	Respiratory agents

Supplementary Table 28: Continuation. See Supplementary Table 27 for column description.

i	$deg(i)$	$degstr(i)$	$betweenness(i)$	$PI(i)$	class
Phenytoin	24	6.51	0.30	0.20	CNS agents
Phenobarbital	15	2.17	0.28	0.05	CNS agents
Ethinyl Estradiol	9	1.78	0.03	0.04	Hormones
Doxycycline	8	1.39	0.02	0.04	Anti-infectives
Prednisone	7	0.96	0.02	0.03	Hormones
Prednisolone	6	0.54	0.03	0.00	Hormones
Estradiol	4	0.57	0.00	0.01	Hormones
Estrogens Conj.	4	0.58	0.00	0.01	Hormones
Dexamethasone	3	0.24	0.00	0.03	Hormones
Norethisterone	3	0.73	0.00	0.00	Hormones
Hydrocortisone	3	0.06	0.20	0.01	Hormones
Levonorgestrel	3	0.79	0.00	0.00	Hormones
Medroxyproges. Ac.	3	1.06	0.00	0.00	Hormones
Folic acid	2	0.50	0.00	0.00	Nutritional Products
Amoxicillin	2	0.30	0.00	0.00	Anti-infectives
Clavulanate	2	0.23	0.00	0.00	Anti-infectives
Sulfadiazine	1	0.51	0.00	0.01	Anti-infectives
Trimethoprim	1	0.16	0.00	0.00	Anti-infectives
Propranolol	14	4.81	0.06	0.10	Cardiovascular agents
Methyldopa	7	2.30	0.01	0.06	Cardiovascular agents
Glyburide	5	2.29	0.00	0.12	Metabolic agents
Atenolol	8	2.22	0.05	0.06	Cardiovascular agents
Aminophylline	10	1.93	0.23	0.01	Respiratory agents
Carvedilol	6	1.70	0.00	0.07	Cardiovascular agents
Gliclazide	5	1.64	0.00	0.12	Metabolic agents
ASA	7	1.57	0.01	0.07	CNS agents
Salbutamol	7	1.53	0.00	0.03	Respiratory agents
Ibuprofen	7	1.28	0.06	0.05	CNS agents
Iron (II) Sulfate	5	1.12	0.02	0.04	Nutritional Products
Timolol	7	1.11	0.16	0.06	Cardiovascular agents
Verapamil	4	1.11	0.01	0.04	Cardiovascular agents
Levodopa	3	0.97	0.03	0.01	CNS agents
Ciprofloxacin	4	0.35	0.21	0.01	Anti-infectives
Chlorpromazine	1	0.33	0.00	0.00	CNS agents
Norfloxacin	2	0.29	0.00	0.01	Anti-infectives
Maprotiline	1	0.23	0.00	0.01	Psychotherapeutic agents
Metoclopramide	1	0.11	0.00	0.00	Gastrointestinal agents

Supplementary Table 29: InfoMap modules of weighted version of network Δ where weights are defined by $\tau^{\Phi}_{i,j}$. Each InfoMap module is shown separated by a horizontal line. Drugs nodes (i; 1nd column) and their respective degree, total degree strength (, and betweenness centrality measure, shown in columns 2, 3, and 4, respectively Column 5 shows the drug probability of interaction, $PI(i)$. Drug class is shown in column 6. Continues on Supplementary Table 30.

i	$deg(i)$	$degstr(i)$	$betweenness(i)$	$PI(i)$	class
Carbamazepine	18	4.84	0.20	0.18	CNS agents
Fluoxetine	10	3.41	0.02	0.06	Psychotherapeutic agents
Fluconazole	10	1.74	0.09	0.11	Anti-infectives
Amitriptyline	5	1.47	0.00	0.08	Psychotherapeutic agents
Imipramine	5	1.31	0.01	0.07	Psychotherapeutic agents
Nortriptyline	5	1.30	0.00	0.09	Psychotherapeutic agents
Fenoterol	8	0.81	0.13	0.01	Respiratory agents
Methylphenidate	1	0.24	0.00	0.02	CNS agents
Digoxin	9	3.70	0.03	0.24	Cardiovascular agents
Warfarin	14	3.31	0.17	0.13	Coagulation modifiers
Furosemide	5	1.31	0.05	0.04	Cardiovascular agents
Levothyroxine	3	1.15	0.00	0.01	Hormones
Propylthiouracil	2	0.87	0.00	0.01	Hormones
Hydrochlorothiazide	2	0.69	0.00	0.00	Cardiovascular agents
Spironolactone	1	0.55	0.00	0.02	Cardiovascular agents
Allopurinol	1	0.46	0.00	0.01	Metabolic agents
Acetaminophen	1	0.22	0.00	0.00	CNS agents
Gentamicin	1	0.12	0.00	0.02	Anti-infectives
Tobramycin	1	0.08	0.00	0.00	Topical Agents
Azithromycin	1	0.07	0.00	0.00	Anti-infectives
Haloperidol	6	2.32	0.03	0.20	Psychotherapeutic agents
Lithium	9	2.05	0.13	0.17	Psychotherapeutic agents
Biperiden	1	0.70	0.00	0.13	CNS agents
Losartan	1	0.21	0.00	0.00	Cardiovascular agents
Captopril	1	0.18	0.00	0.00	Cardiovascular agents
Metronidazole	3	0.17	0.16	0.00	Anti-infectives
Enalapril	1	0.16	0.00	0.00	Cardiovascular agents
Diltiazem	6	2.66	0.03	0.13	Cardiovascular agents
Amiodarone	3	1.40	0.00	0.02	Cardiovascular agents
Simvastatin	4	1.07	0.00	0.02	Metabolic agents
Amlodipine	1	0.34	0.00	0.00	Cardiovascular agents
Diazepam	5	1.12	0.05	0.09	CNS agents
Omeprazole	3	0.85	0.00	0.05	Gastrointestinal agents
Clonazepam	2	0.42	0.00	0.09	CNS agents
Erythromycin	5	0.20	0.18	0.01	Anti-infectives
Diclofenac	2	0.09	0.03	0.01	CNS agents
Alendronate	1	0.04	0.00	0.01	Metabolic agents
Epinephrine	1	0.0	0.0	0.02	Respiratory agents

Supplementary Table 30: Continuation. See Supplementary Table 29 for column description.

Supplementary Note 7 Null Model for RI^y

To test if sheer combinatorics explains the increased risk of DDI in older age, we compared the observed risk of interactions RI^y with a random null model, H_0^{rnd}. We separated all patients u in our dataset per age range y. From these subset of patients $U^{[y1,y2]}$ we also separated which drugs d were prescribed in their age range as $D^{[y1-y2]}$. For clarity, we will refer to all measures previously reported with an added star ($\star$) in the notation to indicate that these values are calculated for the null model (e.g., $RI^{y\star}$ is the null model value of the risk of interaction per age range, RI^y).

The null model is then computed by proportionally sampling patients for each age range, $u \in U^{[y1-y2]}$. For each drawn patient u we sampled $|D^u|$ drugs available to patients in the patient's age range $D^{[y1,y2]}$, and then randomly drew Ψ^u co-administrations from the patient's possible pairwise combinations $\binom{|D^u|}{2}$ of drugs, thus yielding random drug pairs $\psi_{i,j}^{u\star}$ that matched the observed number of co-administrations, $\Psi^u \equiv \Psi^{u\star}$. To decide if a co-administration is an interaction in the null model, we compare the randomly drawn pair of drugs against DrugBank to decide if $\varphi_{i,j}^{u\star}$ is an interaction or not.

This null model allow us to measure what is the expected number of interactions given the increase of co-administrations observed with age, assuming drugs are prescribed completely at random. In other words, it measures the risk of DDI if only age, and the drugs available to patients in these ages, were given to them at random with the same number of co-administrations.

To compute confidence intervals for the number of patients in the null model, we proportionally sampled the same number of patients observed in each age range, 100 times. Confidence intervals can be seen as background fills in Figures 2 and 4. To measure the significance of our null models, Supplementary Table 31 shows the chi-square tests against the expected number of patients in each age bin, $|U^{[y1,y2]}|$, from our data. The null model rejects the hypothesis it was sampled from the same distribution as our data. This means the observed increase in DDI with age, seen in our data, cannot be explained alone by the increased combinatorics of drug co-administrations alone.

	model	chi-square	p-value
1	H_0^{rnd}	22378.5912	0.0

Supplementary Table 31: Chi-square statistic when the number of patients in the null model, $|U^{y\star}|$, is compared to the observed values, $|U^y|$.

Supplementary Note 8 Interactions per Neighborhood

Supplementary Figure 12 shows the number of drugs dispensed for each neighborhood N, colored by the average income of its residents, R$ (*Reais*). Naturally, the larger the neighborhood population (Ω^N), the more drugs are dispensed (α^N), leading to a fairly clear linear relationship ($R^2 = 623$, $p = 0.0$). Some observed exceptions above and below the regression line are noteworthy, though. Three neighborhoods—*Itoupavazinha*, *Velha Central* and *Água Verde*—display dispensation levels below what is expected for their population (circled in Cyan in Supplementary Figure 12-left). On the other hand, two neighborhoods that are also not among the wealthiest—*Fortaleza* and *Tribess*—are well above the expected drug dispensation (circled in magenta). Looking at these specific neighborhoods will require further work to be better understood. In any case, their identification highlights the benefits of analyzing EHR and a data science approach to support responsive public health policy.

For a visual inspection of how both official survey numbers and those analyzed in the main manuscript related geographically in the city of Blumenau, we have mapped neighborhoods to results in Supplementary Figures 13 and 14. The first figure denotes neighborhoods mapped to official numbers from IBGE[7], such as population, gender rate and income distribution. The second figure denotes dispensed drug intervals, distinct drugs, co-administrations and interactions mapped to each neighborhood.

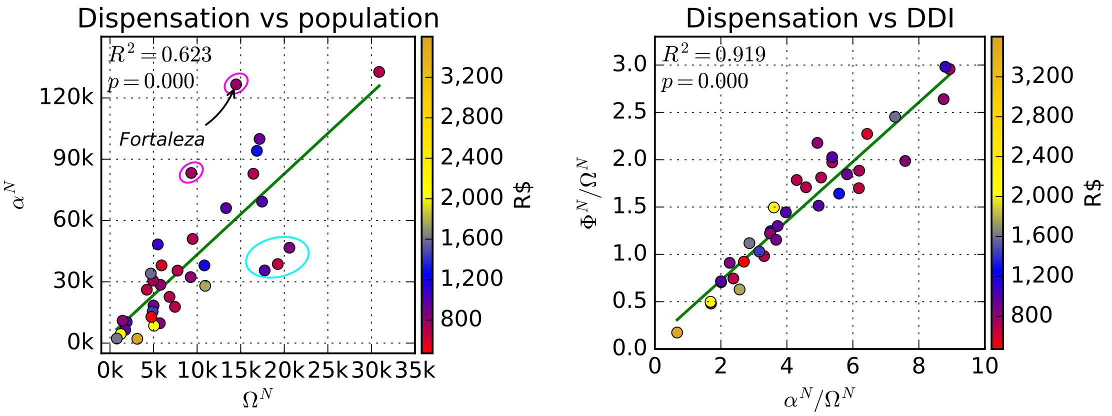

Supplementary Figure 12: Left. Number of drugs intervals dispensed α^N against population Ω^N in each neighborhood N. **Right.** Number of drug intervals dispensed (α^N) versus number of interactions (Φ^N), per neighborhood (N), normalized by population (Ω^N). Color denotes the average per capita income of neighborhood, in Brazilian *Reais* (R$). Regression line shown in green. Patients who reported living in neighborhood *Other* were discarded from computation. Cartographic shapes from IBGE [7].

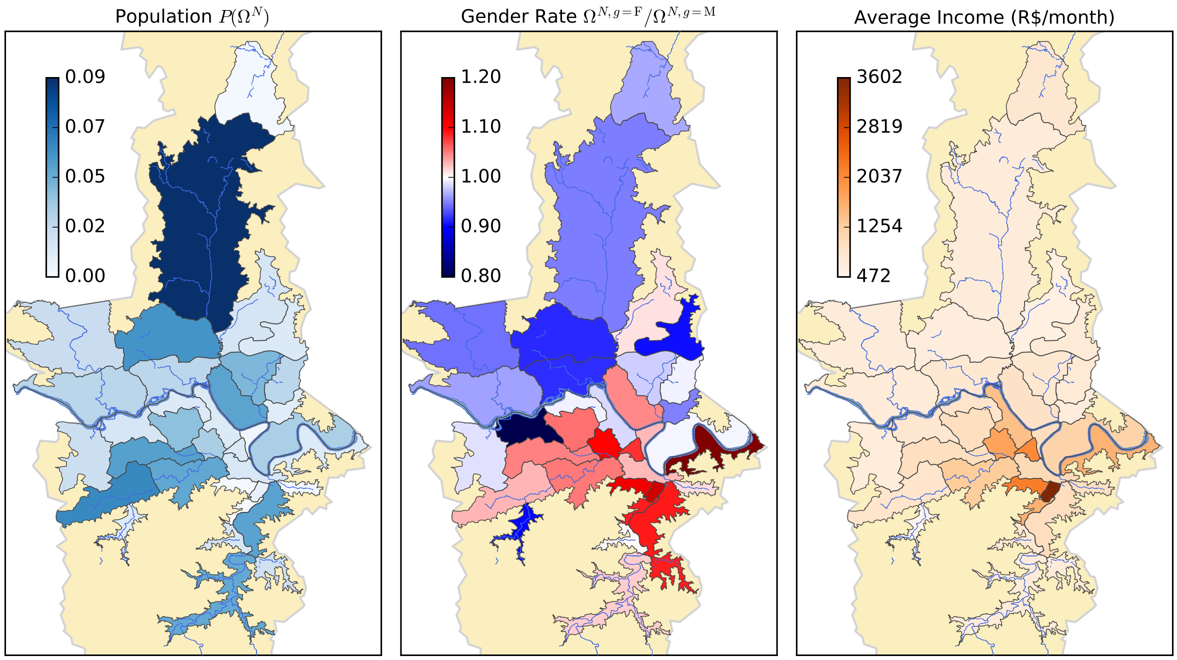

Supplementary Figure 13: Data from IBGE [7] mapped to geographical neighbourhoods in the city of Blumenau. **Left.** Population probability, $P(\Omega^N)$. **Center.** Gender rate, $\Omega^{N,g=F}/\Omega^{N,g=M}$. **Right.** Average income in Brazilian Reais (R$/month). See Supplementary Note 9 for details on income. Cartographic shapes from IBGE [7].

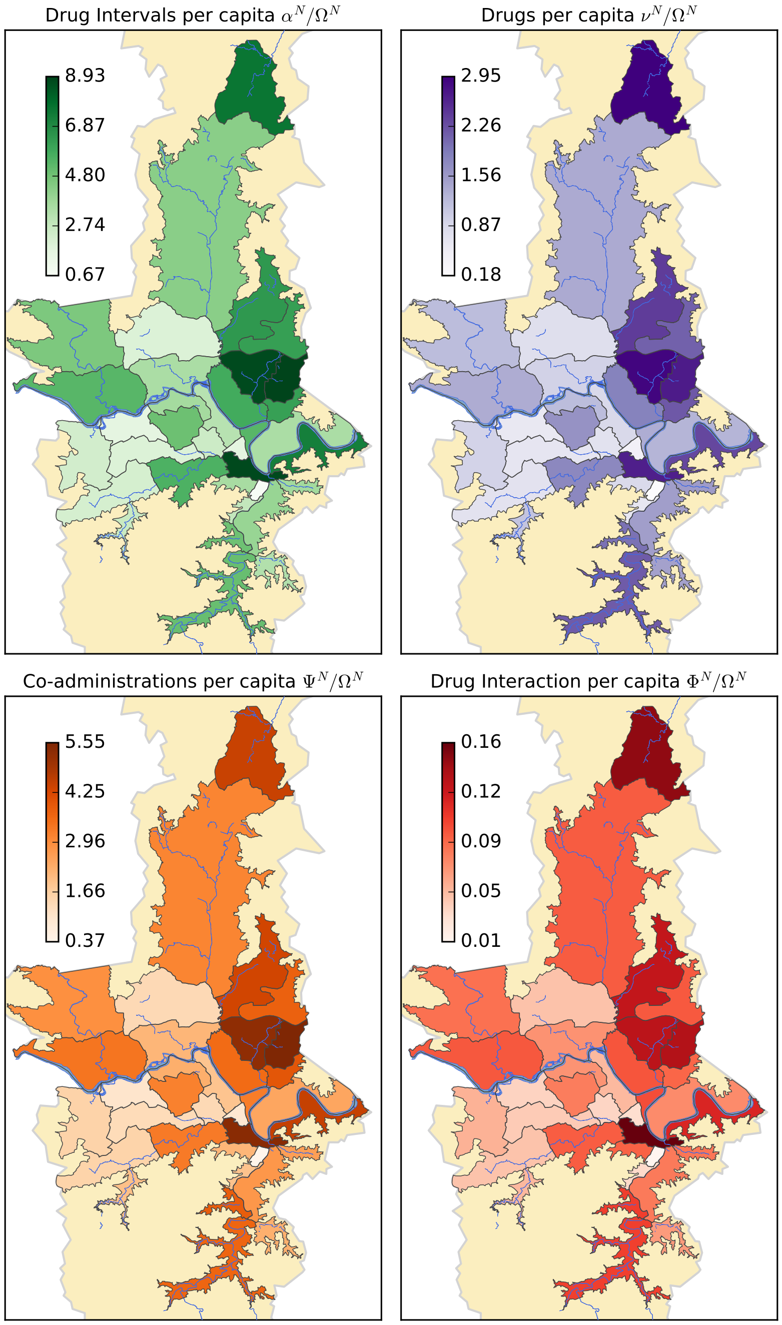

Supplementary Figure 14: Results from Pronto data mapped to geographical neighbourhoods in the city of Blumenau. **Top left.** Number of drug interval dispensed per capita, α^N/Ω^N. **Top right.** Distinct drugs dispensed per capita, ν^N/Ω^N. **Bottom left.** Number of co-adminstrations per capita, Ψ^N/Ω^N. **Bottom right.** Number of interactions per capita, Φ^N/Ω^N. Cartographic shapes from IBGE [7].

Supplementary Note 9 Projected Cost of DDI in hospitalizations

Estimating the financial burden of DDI prescribed in primary and secondary care is difficult, since outcomes vary by a large margin and only few result in short-term symptoms requiring hospitalization. Measuring hospitalizations due to DDI are also strenuous, since underestimation of true risk can be masked in practitioners and pharmacists failing to recognize adverse patient outcomes caused by DDI as such. However, drug- and cohort-focused studies have shown that the number of DDI is associated with a significantly increased risk of hospitalization [21, 22]. A review paper in 2007 [23] estimated that DDI were held responsible for 0.054% of emergency room (ER) visits, 0.57% of hospital admissions (4.8% in the elderly population) and 0.12% of re-hospitalizations. The most common outcomes were gastrointestinal bleeding (32.8%), hypertension/hypotension (18%) and cardiac rhythm disturbances (18%).

In this section a study of the financial burden of possible DDI-related hospitalizations is presented. It considers various rates of hospitalization expected for major DDI co-administrations, and is based on a cost estimate of ADR hospitalizations in Canada [24], and average hospitalization costs for Brazil at city, state and national levels. As average hospitalization costs were not found for the United States, results in Canadian dollars were also converted to US dollars. Our estimation then relies on guessing what proportion of patients with major DDI co-administrations are likely to have an ADR requiring hospitalization.

To compute costs we gathered number of public health care hospitalizations and average costs for each level (see Supplementary Table 32) from the national Hospitalization Information System (*Sistema de Informações Hospitalares do SUS*; SIH/SUS), a data source managed by the Informatics Department under the Executive Secretary of Brazil's Ministry of Health [25].

As reported, the number of patients prescribed a major DDI in Blumenau (city level) was $|U^{\Phi,s=major}| = 5,224$. For state and national levels, we estimated this number from the percentage of hospitalizations it represents at city level, a reasonable assumption due the lack of data that generalizes medical practice in Blumenau for the state and country. For example, say 261 (or 5%) Pronto patients prescribed a major DDI had to be hospitalized. In hospitalization terms, that accounts for 1.06% of all hospitalizations in the same period. At the state and national level, the same 1.06% accounts for 5,376 and 142,564 patients, respectively. Costs are then estimated by multiplying the number of patients assumed hospitalized by the average hospitalization cost in each level.

Wu *et al.* [24] argued in 2007 that the average cost of ADR-related hospitalization for all adults over 65 in the province of Ontario (pop. 12M in 2006; 13.6M in 2014) was C$ 7,528 (C$ 8,443.14 or $7,380.78 in 2014 when adjusted for inflation and exchange rate) for a total annual cost of C$ 13.6 million (C$ 15.2M or $13.3M after adjusting), or estimated C$ 35.7 million (C$ 40M or $35M after adjusting) in Canada. In an attempt to compare results, we also multiplied the number of patients assumed to have been hospitalized to their average cost of ADR-relation hospitalization (see columns 6 and 7 of Supplementary Table 33). Moreover,

		Blumenau city	Santa Catarina state	Brazil national	Ontario province
Population		338,876	6,819,190	204,450,649	13,680,425
Hospitalization	Elective	9,761	146,395	3,391,088	-
	Urgent	24,592 (5,808)	507,189 (110,748)	13,440,043 (2,711,527)	-
	Work Accident	87	2,106	64,485	-
	External Causes	786	902	110,922	-
	Total	35,226	656,592	17,006,538	-
Avg. Cost	Elective	R$ 3,764.62	R$ 1,533.10	R$ 1,583.45	-
	Urgent	R$ 2,606.03	R$ 1,379.13	R$ 1,083.23	C$ 8,443.14
	Work Accident	R$ 1,663.27	R$ 2,595.45	R$ 1,541.38	-
	External Causes	R$ 2,321.31	R$ 2,203.50	R$ 1,256.36	-

Supplementary Table 32: Population, number of hospitalizations, and average cost per hospitalization in the analyzed period shown for city, state and national levels. Population follows the official projections for 2015. Hospitalization numbers and cost shown by type. Urgent hospitalization values in parenthesis shown for patients over 64 years old. Note Blumenau has a much higher average cost per hospitalization than state and national levels. Brazil data from Hospitalization Information System (*Sistema de Informações Hospitalares do SUS; SIH/SUS*) [25]. Ontario data from Wu *et al.* [24], adjusted for inflation.

Supplementary Tables 33, 34 and 35 show the estimated costs at different percentages of hospitalizations at city, state and national levels, respectively. Costs in Brazilian Reais (columns 4 and 5) are computed based on the average cost of hospitalization in Brazil. Costs in C$ use Wu *et al.* [24] as reference (columns 6 and 7), and then converted to US$ with the average exchange rate between the two currencies for the whole period of our data (columns 8 and 9). The average exchange rate in the period was C$1.00 Canadian dollar equals to $0.8742 US dollar, and maximum and minimum rates were .9418 in January 4th 2014 and .7821 in March 14th 2015, respectively.

Our cost analysis first considers two previous studies of the proportion of emergency room visits that are due to DDI and ADR. Becker *et al.* [23] reported that 0.57% of all hospital admissions they observed were due to DDI, which in our data would correspond to 140 patients, or $p_h = 2.68\%$ of all patients dispensed a major DDI (see Supplementary Table 33). Wu *et al.* [24], on the other hand, argued that 0.75% of all hospitalizations of patients over 65 years of age were due to ADR (not only from DDI), which corresponds in our data to 436 patients, or $p_h = 8.35\%$ of all patients who were co-administered a major DDI. Both of these conjectures are likely to err on the side of under-reporting emergency room admissions due to DDI or ADR, since this a well-known problem in studies of this phenomenon [26–30]. Indeed, the proportion of hospital admissions due to ADR has been reported in the literature to vary anywhere between 0.5% to 12% [28], with DDI reportedly being responsible for 15% [29] to 30% [31] of all ADR. These ranges, if correct, put the proportion of hospital admissions due to DDI anywhere between $0.5\% \times 15\% = 0.075\%$ and $12\% \times 30\% = 3.6\%$, which in our Blumenau data would mean between 18 and 885 emergency room patients, or $p_h \in [0.35, 16.95]\%$ of all patients dispensed a major DDI. These ranges could be higher, since even in the more controlled hospital environment, the proportion of patients with ADR can be as high as 41% [32]. Therefore, in addition to the costs derived from the numbers provided by Becker *et al.* [23] and Wu *et al.* [24], tables in this section also report cost estimates for various values of p_h, so that readers can judge what is an appropriate value to consider. Given the ranges just outlined, it is reasonable to assume, for instance, that $p_h = 10\%$ of all patients dispensed a major DDI will have to be hospitalized (522 patients).

The lowest estimate ($p_h = 2.68\%$, via Becker *et al.* [23]) leads to a cost of DDI-related hospitalization in Blumenau of over $1M in the 18-month period, after adjusting for Canadian cost, inflation and exchange rate to US dollar. The extrapolated costs to the state and the country are $21M and $565M, respectively (see Supplementary Tables 34 and 35). The estimated costs obtained via Wu *et al.* [24] ($p_h = 8.35\%$) reach $3.2M, $61M, and $1.5B, for the city, state and country levels respectively. Finally, if we assume $p_h = 10\%$, the estimated costs reach $3.9M, $79M, and $2.1B, for the city, state and country levels respectively.

All estimations lead to very substantial costs for the various levels of government. To compare them to the costs found for Canada [24], we computed per capita measures of the burden of DDI-related hospitalizations. For instance, the lowest estimate $p_h = 2.68\%$ leads to a per capita cost for Blumenau of $2.03, while the inflation-adjusted cost for Ontario (Canada) is $0.97, suggesting that the financial burden of DDI is more severe than previously thought—even when considering only the lowest estimate of the proportion of hospitalizations that derive from co-administration of known major DDIs. For the state of Santa Catarina and Brazil as a whole, these numbers are $2.09 and $1.84, respectively. If we consider the higher estimates of $p_h = 8.35\%$ or $p_h = 10\%$, the per capita cost for Blumenau is $6.33 and $7.58, respectively.

To put these numbers in context, Brazil's minimum monthly wage was R$724 (R$9,412/year[3]) in 2014, and workers in Blumenau received on average 2.9 wages a month [7]. This constitutes an average gross income of R$2,099.60 a month (R$27,294.80/year). If we assume the same 140 patients were hospitalized due to ADR caused by DDI, the direct cost of such hospitalizations is equal to 3,707 lost productive worker/days (considering an 8 hour working day), with possible much higher indirect costs.

Some limitations should be noted. When comparing to Becker *et al.* [23], data from IBGE–Instituto Brasileiro de Geografia e Estatística [7] includes patients over 64, while in their work the authors included patients over 65 years old. Our analysis then possibly contains additional patients exactly age 65, although we do not believe this affects the results presented given their large difference. In general, other studies [23] divide hospital admissions only between two categories, emergency room (ER) visits and hospitalizations. It is not possible to conclude whether electives or external causes are included in their hospitalization numbers. SIH/SUS data are only available for patients that were hospitalized in the public system, meaning

[3]Brazilians receive a 13th salary in December. Thus, yearly gross income is calculated by a 13, and not by a 12, multiplier.

p_h	$\lvert U^{\Phi}_{major}\rvert$	% of hosp.	Cost R$ 18 months	Cost R$ 12 months	Cost CA$ 18 months	Cost CA$ 12 months	Cost US$ 18 months	Cost US$ 12 months
100%	5,224	21.24%	13,613,909	9,075,940	44,106,963	29,404,642	38,557,213	25,704,809
50%	2,612	10.62%	6,806,955	4,537,970	22,053,482	14,702,321	19,278,606	12,852,404
30%	1,567	6.37%	4,083,652	2,722,434	13,230,400	8,820,267	11,565,688	7,710,458
25%	1,306	5.31%	3,403,477	2,268,985	11,026,741	7,351,161	9,639,303	6,426,202
20%	1,044	4.25%	2,720,697	1,813,798	8,814,638	5,876,425	7,705,538	5,137,025
10%	522	2.12%	1,360,349	906,899	4,407,319	2,938,213	3,852,769	2,568,513
5%	261	1.06%	680,174	453,450	2,203,660	1,469,106	1,926,384	1,284,256
2.68%	140	0.57%	364,844	243,230	1,182,040	788,026	1,033,310	688,873
8.35%	436	0.75%	1,136,230	757,487	3,681,209	2,454,139	3,218,022	2,145,348

Supplementary Table 33: Projected cost of DDI for the city of Blumenau in Reais (R$), Canadian Dollars (C$) and US dollars (US$) for the analysis period (18 months) and yearly (12 months). Each row calculates the associated cost based on different proportion of patients who had at least one major DDI and required hospitalization. Last row shows the projected cost when only 0.75% of all hospitalizations of patients over 64 years old are considered, based on results of Wu *et al.* [24]. Similarly, second-to-last row shows projected cost when only 0.57% of all hospitalization are considered, based on results of Becker *et al.* [23]. In the 18 month period, Blumenau had a total of 24,592 public health care emergency hospitalizations, from which 5,808 were of patients age over 64 years old. Average cost per hospitalization in the city is R$ 2,606.03. US$ costs were calculated based on C$ exchange rate of .8742, the average rate in the study period.

p_h	$\lvert U^{\Phi}_{major}\rvert$	% of hosp.	Cost in R$ 18 months	Cost in R$ 12 months	Cost in CA$ 18 months	Cost in CA$ 12 months	Cost in US$ 18 months	Cost in US$ 12 months
100%	107,726	21.24%	148,567,620	99,045,080	909,545,700	606,363,800	795,102,280	530,068,187
50%	53,863	10.62%	74,283,810	49,522,540	454,772,850	303,181,900	397,551,140	265,034,093
30%	32,307	6.37%	44,555,391	29,703,594	272,772,524	181,848,349	238,450,972	158,967,314
25%	26,931	5.31%	37,141,215	24,760,810	227,382,203	151,588,136	198,771,880	132,514,586
20%	21,555	4.25%	29,727,039	19,818,026	181,991,883	121,327,922	159,092,788	106,061,859
10%	10,752	2.12%	14,828,352	9,885,568	90,780,641	60,520,428	79,358,184	52,905,456
5%	5,376	1.06%	7,414,176	4,942,784	45,390,321	30,260,214	39,679,092	26,452,728
2.68%	2,890	0.57%	3,985,671	2,657,114	24,400,675	16,267,116	21,330,464	14,220,309
7.71%	8,306	0.75%	21,645,699	14,430,466	70,128,721	46,752,481	61,304,788	40,869,858

Supplementary Table 34: Projected cost of DDI for the state of Santa Catarina in Reais (R$), Canadian Dollars (C$) and US dollars (US$) for the analysis period (18 months) and yearly (12 months). Each row calculates the associated cost based on different proportion of patients who had at least one major DDI and required hospitalization. Last row shows the projected cost when only 0.75% of all hospitalizations of patients over 64 years old are considered, based on results of Wu *et al.* [24]. Similarly, second-to-last row shows projected cost when only 0.57% of all hospitalization are considered, based on results of Becker *et al.* [23]. In the 18 month period, Santa Catarina had a total of 507,189 public health care emergency hospitalizations. Average cost per hospitalization in the state is R$ 1,379.13. US$ costs were calculated based on C$ exchange rate of .8742, the average rate in the study period.

the cost of hospitalization was billed to the public system. Therefore, if a patient was hospitalized and his/her private insurance covered the costs, the SIH/SUS would have no record of it. Furthermore, SIH/SUS provides the number of hospitalizations broken down by type. These consist of "electives" (e.g., schedules cesareans), "urgencies", "work accidents", and "other external causes" (codes V01 to Y98 of ICD-10[4]; e.g., car accident, poisoning, and drowning). To better approximate reality, we have calculated the cost of DDI-related hospitalizations only using the number of urgent hospitalizations.

[4] http://www.datasus.gov.br/cid10/V2008/WebHelp/v01_y98.htm

p_h	$\lvert U^{\Phi}_{major}\rvert$	% of hosp.	Cost in R$ 18 months	Cost in R$ 12 months	Cost in CA$ 18 months	Cost in CA$ 12 months	Cost in US$ 18 months	Cost in US$ 12 months
100%	2,854,665	21.24%	3,092M	2,061M	24,102M	16,068M	21,070M	14,046M
50%	1,427,332	10.62%	1,546M	1,031M	12,051M	8,034M	10,535M	7,023M
30%	856,130	6.37%	927M	618M	7,228M	4,819M	6,319M	4,213M
25%	713,666	5.31%	773M	515M	6,026M	4,017M	5,267M	3,512M
20%	571,201	4.25%	619M	412M	4,823M	3,215M	4,216M	2,811M
10%	284,928	2.12%	309M	206M	2,406M	1,604M	2,103M	1,402M
5%	142,464	1.06%	154M	103M	1,203M	802M	1,051M	701M
2.68%	76,608	0.57%	83M	55M	647M	431M	565M	377M
7.12%	203,365	0.75%	530M	353M	1,717M	1,145M	1,501M	1,001M

Supplementary Table 35: Projected cost of DDI for Brazil in Reais (R$), Canadian Dollars (C$) and US dollars (US$) for the analysis period (18 months) and yearly (12 months). Each row calculates the associated cost based on different proportion of patients who had at least one major DDI and required hospitalization. Last row shows the projected cost when only 0.75% of all hospitalizations of patients over 64 years old are considered, based on results of Wu *et al.* [24]. Similarly, second-to-last row shows projected cost when only 0.57% of all hospitalization are considered, based on results of Becker *et al.* [23]. In the 18 month period, Brazil had a total of 13,440,043 public health care emergency hospitalizations. Average cost per hospitalization in the country is R$ 1,083.23. US$ costs were calculated based on C$ exchange rate of .8742, the average rate in the study period.

Supplementary Note 10 Statistical modeling

Here we show the complete results from runs of simple regression (SR), polynomial regression (PR), ordinary multiple regression (OMR) and linear mixed model (LMM) that were mentioned in the original text. For further details on LMM see [33].

A SR is is a linear regression model with a single explanatory variable. It works by fitting a line through the data that minimizes the sum of the squared of the residual, where the residuals are differences between the observed values to the predicted values of the model. For example, when predicting the number of interactions from the number co-administrations, the SR equation would look like

$$\Phi^u(x) = \beta^0 + \beta^1 x + \epsilon^u \tag{16}$$

where β^0 is the intercept (or bias), β^1 is the coefficient (or slope) and ϵ^u are the residuals.

Similarly, PR is a regression where the dependent variable being modeled is fitted with a nth degree polynomial, but still a single explanatory variable. In the main manuscript we modeled RRI^y with a cubic (3rd degree) polynomial. In our case, the PR equation would look like

$$RRI^y(x) = \beta^0 + \beta^1(x^3) + \beta^2(x^2) + \beta^3 x + \epsilon^y \tag{17}$$

where β^0 is the intercept (or bias), β^1, β^2 and β^3 are coefficients (giving the characteristic shape of the cubic curve) and ϵ^y are the residuals.

An OMR is a widely used type of regression for predicting the value of one dependent variable from the value of a set of independent variables. Similar to SR, it works by fitting a hyperplane through the data that minimizes the sum of the squared of the residuals.

In our case, the OMR equation would look like

$$\Phi^u = \beta^0 + \beta^1 x^{u,1} + \beta^2 x^{u,2} + \ \dots\ \beta^j x^{u,i} + \epsilon^u \tag{18}$$

Where β^0 is the intercept (or bias), β^j are the coefficients (or slopes) and each $x^{u,i}$ is a predictor (covariate, regressor) such as age or number of drugs. ϵ^u is the error associated with the fit.

A LMM (also known as multilevel, mixed effects, random effects or hierarchical linear model) can be seen as extensions of the OMR where instances of the data belong to certain groups—like children in classrooms or cities in states. In our case, they are patients in specific neighborhoods.

The individual levels are usually defined as level-1 (within-group), and level-2 (between-group) for a two level model. Separate level-1 models (e.g., patients) are developed for each level-2 (e.g., neighborhoods).

Considering only one predictor, level-1 models take the form of simple regressions:

$$\Phi^{u,n} = \beta^{0,n} + \beta^{1,n} x^{u,n} + r^{u,n} \tag{19}$$

where $\beta^{0,n}$ is the intercept for the n neighborhood, $\beta^{1,n}$ is a coefficient (slope) associated with predictor $x^{u,n}$, and $r^{u,n}$ is the error.

In the level-2 models, the level-1 regression coefficients ($\beta^{0,n}$ and $\beta^{1,n}$) are used as outcome variables and are related to each of the level-2 predictors.

$$\beta^{0,n} = \gamma^{0,0} + \gamma^{0,1} g^n + u^{0,n} \tag{20}$$

$$\beta^{1,n} = \gamma^{1,0} + \gamma^{1,1} g^n + u^{1,n} \tag{21}$$

where g^n is the level-2 predictor, $\gamma^{0,0}$ and $\gamma^{1,0}$ are the overall mean intercept adjusted for g. $\gamma^{0,1}$ ($\gamma^{1,1}$) is the regression coefficient associated with g relative to level-1 intercept (slope) and $u^{0,n}$ ($u^{1,n}$) are level-2 random effects adjusted for g on the intercept (slope).

A combined two-level model is created by substituting Supplementary Equations 20 and 21 into Supplementary Equation 19:

$$\Phi^{u,n} = \gamma^{0,0} + \gamma^{1,0} x^{u,n} + \gamma^{0,1} g^n + \gamma^{1,1} g^n x^{u,n} + u^{1,n} x^{0,n} + u^{0,n} + \epsilon^{u,n} \tag{22}$$

The combined model incorporates the level-1 and level-2 predictors ($x^{u,n}$ and g^n), a cross-level term ($g^n x^{u,n}$) as well as the composite error ($u^{1,n} x^{u,n} + u^{0,n} + r^{u,n}$).

In practice, LMM coefficients are estimated using maximum likelihood methods.

10.1 Simple Regression (SR) models

In Figure 3 of the main manuscript we show single regression models predicting the number of interactions. Specifically, ν^u predicts Ψ^u best with a quadratic regression ($R^2 = .857$) as shown in Figure 3-left. When it comes to predicting number of interactions (Figure 3, center and right), on the other hand, there is much more dispersion of the data, which leads to a relatively small linear correlation between Ψ^u and Φ^u ($R^2 = .487$)—though better than the linear correlation between ν^u and Φ^u ($R^2 = .304$). However, higher order regressions do not improve the prediction of the variance of Φ^u, as demonstrated by the Pareto front in Fig. 3-top-right (see also Supplementary Note 11)—thus discarding the hypothesis of a clear nonlinear relationship between co-administrations and interactions, which could explain the growth of RI with age.

Supplementary Tables below display additional regression models.

Listing 1: Ψ^u from ν^u linear model

```
===================================================
                                Ψ^u
ν^u                        3.891*** (0.007)
Constant                      -8.818*** (0.037)
---------------------------------------------------
Observations                       132,722
R2                                  0.712
Adjusted R2                         0.712
Residual Std. Error          8.650 (df = 132720)
F Statistic            328,478.000*** (df = 1; 132720)
===================================================
Note:                      *p<0.1; **p<0.05; ***p<0.01
```

Listing 2: Ψ^u from ν^u quadratic model

```
===================================================
                                Ψ^u
ν^u                        -0.121*** (0.012)
(ν^u)^2                0.273*** (0.001)
Constant                       -0.023 (0.036)
---------------------------------------------------
Observations                       132,722
R2                                  0.857
```

```
Adjusted R2                      0.857
Residual Std. Error        6.088 (df = 132719)
F Statistic          399,075.300*** (df = 2; 132719)
===================================================
Note:                    *p<0.1; **p<0.05; ***p<0.01
```

Listing 3: Φ^u from ν^u linear model

```
==================================================
                              Φ^u
ν^u                     0.110*** (0.0005)
Constant                   -0.267*** (0.003)
--------------------------------------------------
Observations                    132,722
R2                               0.304
Adjusted R2                      0.304
Residual Std. Error       0.580 (df = 132720)
F Statistic          58,011.640*** (df = 1; 132720)
==================================================
Note:                   *p<0.1; **p<0.05; ***p<0.01
```

Listing 4: Φ^u from ν^u quadratic model

```
==================================================
                              Φ^u
ν^u                     -0.009*** (0.001)
(ν^u)^2             0.008*** (0.0001)
Constant                    -0.007** (0.003)
--------------------------------------------------
Observations                    132,722
R2                               0.372
Adjusted R2                      0.372
Residual Std. Error       0.551 (df = 132719)
F Statistic          39,357.930*** (df = 2; 132719)
==================================================
Note:                   *p<0.1; **p<0.05; ***p<0.01
```

Listing 5: Φ^u from Ψ^u linear model

```
===================================================
                              Φ^u
Ψ^u                     0.030*** (0.0001)
Constant                    -0.033*** (0.002)
---------------------------------------------------
Observations                     132,722
R2                                0.487
Adjusted R2                       0.487
Residual Std. Error        0.498 (df = 132720)
F Statistic          126,232.900*** (df = 1; 132720)
===================================================
Note:                    *p<0.1; **p<0.05; ***p<0.01
```

10.1.1 RC^y models

In Figure 2 of the main manuscript two regressions were calculated to predict the growth of RC^y and RI^y based on age range ($y = [y1 - y2]$). Both RC^y and RI^y can be best approximated by a cubic polynomial regression (see Fig. 2 for R^2) The regression lines show different growth processes for co-administration and interaction risks. RC^y first decreases in children age range [5-14], followed by an almost flat level between ages [15,44] before a steeper growth is observed for older age groups (see shaded area in Fig 2-left). In contrast, RI^y is initially quite flat and only starts to increase after the age of 15, after which it has a much steeper growth curve than $RC^[y]$ (note the difference in scale).

In addition, Supplementary Tables below contain other regression models that were computed along with their respective ANOVA comparison, when appropriate.

A linear model is the simplest model one could fit to the increased risk of co-administration.

Listing 6: RC^y linear model

```
===============================================
```

```
                              RC^y
-----------------------------------------------
y                     0.003*** (0.0004)
Constant                 0.926*** (0.004)
-----------------------------------------------
Observations                    19
R2                             0.798
Adjusted R2                    0.787
Residual Std. Error       0.009 (df = 17)
F Statistic           67.336*** (df = 1; 17)
===============================================
Note:               *p<0.1; **p<0.05; ***p<0.01
```

A quadratic model fits slightly better but the increased model complexity is not significant.

Listing 7: RC^y quadratic model

```
===============================================
                              RC^y
-----------------------------------------------
(y)^2                  0.0001 (0.0001)
y                          0.001 (0.001)
Constant                 0.931*** (0.005)
-----------------------------------------------
Observations                    19
R2                             0.820
Adjusted R2                    0.798
Residual Std. Error       0.009 (df = 16)
F Statistic            36.493*** (df = 2; 16)
-----------------------------------------------
Model 1: RC^y ~ y
Model 2: RC^y ~ (y)^2 + y
  Res.Df       RSS Df  Sum of Sq      F Pr(>F)
1     17 0.0013609
2     16 0.0012139  1 0.00014698 1.9374  0.183
===============================================
Note:               *p<0.1; **p<0.05; ***p<0.01
```

A cubic model gives almost perfect fit while being significant for the more complex model.

Listing 8: RC^y cubic model

```
===============================================
                              RC^y
-----------------------------------------------
(y)^3                -0.0001*** (0.00001)
(y)^2                 0.001*** (0.0003)
y                       -0.008*** (0.002)
Constant                 0.943*** (0.004)
-----------------------------------------------
Observations                    19
R2                             0.936
Adjusted R2                    0.923
Residual Std. Error       0.005 (df = 15)
F Statistic           72.789*** (df = 3; 15)
-----------------------------------------------
Model 1: RC^y ~ y
Model 2: RC^y ~ (y)^2 + y
Model 3: RC^y ~ (y)^3 + (y)^2 + y
  Res.Df        RSS Df  Sum of Sq       F    Pr(>F)
1     17 0.00136086
2     16 0.00121387  1 0.00014698  5.0807 0.0395787 *
3     15 0.00043394  1 0.00077993 26.9599 0.0001094 ***
===============================================
Note:               *p<0.1; **p<0.05; ***p<0.01
```

10.1.2 RI^y models

Similarly to how we modeled RI^y, with the risk of known DDI co-administration (RI^y) we start with the simplest linear model possible.

Listing 9: RI^y linear model

```
===============================================
```

```
                              RI^y
-----------------------------------------------
y                      0.024*** (0.002)
Constant                   -0.032* (0.016)
-----------------------------------------------
Observations                    19
R2                             0.932
Adjusted R2                    0.928
Residual Std. Error        0.037 (df = 17)
F Statistic            233.631*** (df = 1; 17)
===============================================
Note:                *p<0.1; **p<0.05; ***p<0.01
```

A quadratic model fits slightly better but the increased model complexity is not significant.

Listing 10: RI^y quadratic model

```
===============================================
                              RI^y
-----------------------------------------------
(y)^2                 -0.0004 (0.0003)
y                      0.030*** (0.006)
Constant                  -0.050** (0.023)
-----------------------------------------------
Observations                    19
R2                             0.937
Adjusted R2                    0.930
Residual Std. Error        0.037 (df = 16)
F Statistic            119.823*** (df = 2; 16)
-----------------------------------------------
Model 1: RC^y ~ y
Model 2: RC^y ~ (y)^2 + y
  Res.Df      RSS Df Sum of Sq      F Pr(>F)
1     17 0.023355
2     16 0.021550  1 0.001805 1.3401 0.264
===============================================
Note:                *p<0.1; **p<0.05; ***p<0.01
```

Finally, a cubic model gives us almost perfect fit while being significant for the more complex model.

Listing 11: RI^y Cubic model

```
===============================================
                              RI^y
-----------------------------------------------
(y)^3                  -0.0003*** (0.00001)
(y)^2                   0.007*** (0.0004)
y                          -0.019*** (0.003)
Constant                      0.013** (0.006)
-----------------------------------------------
Observations                    19
R2                             0.997
Adjusted R2                    0.997
Residual Std. Error        0.008 (df = 15)
F Statistic           1,927.479*** (df = 3; 15)
-----------------------------------------------
Model 1: RI^y ~ y
Model 2: RI^y ~ (y)^2 + y
Model 3: RI^y ~ (y)^3 + (y)^2 + y
  Res.Df       RSS Df Sum of Sq       F    Pr(>F)
1     17 0.0233550
2     16 0.0215500  1  0.001805  30.391 5.96e-05 ***
3     15 0.0008909  1  0.020659 347.842 8.66e-12 ***
===============================================
Note:                *p<0.1; **p<0.05; ***p<0.01
```

10.2 Multiple Regression (MR) models

This section displays several MR models that were generated in order to analyze the possible prediction of drug interaction based on patient demographics. Tables below contain the model results and also their respective ANOVA comparison when appropriate.

10.2.1 Baseline (no transformation)

This is the baseline MR model with no transformation.

Listing 12: Baseline linear regression model

```
==================================================
                              $\Phi^u$
--------------------------------------------------
$\nu^u$                       -0.026*** (0.001)
$\Psi^u$                     0.035*** (0.0002)
Constant                      0.041*** (0.003)
--------------------------------------------------
Observations                      132,722
R2                                 0.492
Adjusted R2                        0.492
Residual Std. Error       0.496 (df = 132719)
F Statistic          64,377.810*** (df = 2; 132719)
==================================================
Note:                  *p<0.1; **p<0.05; ***p<0.01
```

10.2.2 Baseline (transformed)

These are other baseline MR model with transformed variables

Listing 13: Transformed baseline MR model

```
==================================================
                              $\Phi^u$
--------------------------------------------------
$\nu^u$                       -0.004*** (0.001)
$\Psi^u$                     0.040*** (0.0002)
$(\nu^u)^2$                 -0.003*** (0.0001)
Constant                      -0.006** (0.003)
--------------------------------------------------
Observations                      132,722
R2                                 0.497
Adjusted R2                        0.497
Residual Std. Error       0.493 (df = 132718)
F Statistic          43,696.240*** (df = 3; 132718)
--------------------------------------------------
Model 1: $\Phi^u$ ~ $\nu^u$ + $\Psi^u$
Model 2: $\Phi^u$ ~ $\nu^u$ + $\Psi^u$ + $(\nu^u)^2$
  Res.Df   RSS Df Sum of Sq      F    Pr(>F)
1 132719 32592
2 132718 32304  1    288.37 1184.7 < 2.2e-16 ***
==================================================
                              $\Phi^u$
--------------------------------------------------
$\nu^u$                       -0.033*** (0.001)
$\Psi^u$                     0.038*** (0.0002)
$(\Psi^u)^2$              -0.00002*** (0.00000)
Constant                      0.053*** (0.003)
--------------------------------------------------
Observations                      132,722
R2                                 0.494
Adjusted R2                        0.494
Residual Std. Error       0.495 (df = 132718)
F Statistic          43,145.430*** (df = 3; 132718)
--------------------------------------------------
Model 1: $\Phi^u$ ~ $\nu^u$ + $\Psi^u$
Model 2: $\Phi^u$ ~ $\nu^u$ + $\Psi^u$ + $(\Psi^u)^2$
  Res.Df   RSS Df Sum of Sq      F    Pr(>F)
1 132719 32592
2 132718 32508  1    84.745 345.99 < 2.2e-16 ***
==================================================
                              $\Phi^u$
--------------------------------------------------
$\nu^u$                       -0.008*** (0.001)
$\Psi^u$                     0.041*** (0.0003)
$(\nu^u)^2$                 -0.003*** (0.0001)
$(\Psi^u)^2$              -0.00000*** (0.00000)
Constant                        0.001 (0.003)
--------------------------------------------------
Observations                      132,722
```

```
R2                                0.497
Adjusted R2                       0.497
Residual Std. Error      0.493 (df = 132717)
F Statistic         32,786.680*** (df = 4; 132717)
--------------------------------------------------
Model 1: Φ^u ~ ν^u + Ψ^u
Model 2: Φ^u ~ ν^u + Ψ^u + (ν^u)^2 + (Ψ^u)^2
  Res.Df   RSS Df Sum of Sq      F    Pr(>F)
1 132719 32592
2 132717 32297  2    295.59 607.33 < 2.2e-16 ***
==================================================
Note:                 *p<0.1; **p<0.05; ***p<0.01
```

10.2.3 Baseline + age + gender

This section shows the MR results when age and gender are included as dependent variables in the baseline model.

Listing 14: Baseline MR model added variables age and gender.

```
==================================================
                               Φ^u
--------------------------------------------------
ν^u                     -0.027*** (0.001)
Ψ^u                     0.034*** (0.0002)
age                         0.002*** (0.0001)
C(gender)Male               -0.010*** (0.003)
Constant                    -0.021*** (0.004)
--------------------------------------------------
Observations                     132,722
R2                                0.496
Adjusted R2                       0.496
Residual Std. Error      0.494 (df = 132717)
F Statistic         32,639.900*** (df = 4; 132717)
--------------------------------------------------
Model 1: Φ^u ~ ν^u + Ψ^u
Model 2: Φ^u ~ ν^u + Ψ^u + age + C(gender)
  Res.Df   RSS Df Sum of Sq      F    Pr(>F)
1 132719 32592
2 132717 32369  2    223.56 458.33 < 2.2e-16 ***
==================================================
Note:                 *p<0.1; **p<0.05; ***p<0.01
```

10.2.4 Baseline (replacing Ψ^u with y)

Interestingly, number of co-administrations (Ψ^u) and age (y) are virtually exchangeable.

Listing 15: Baseline MR model exchanging variables Ψ^u and y.

```
==================================================
                               Φ^u
--------------------------------------------------
Ψ^u                     0.029*** (0.0001)
age                         0.002*** (0.0001)
Constant                    -0.100*** (0.003)
--------------------------------------------------
Observations                     132,722
R2                                0.491
Adjusted R2                       0.491
Residual Std. Error      0.496 (df = 132719)
F Statistic         63,937.920*** (df = 2; 132719)
--------------------------------------------------
Model 1: Φ^u ~ ν^u + Ψ^u
Model 2: Φ^u ~ Ψ^u + age
  Res.Df   RSS Df Sum of Sq F Pr(>F)
1 132719 32592
2 132719 32702  0   -110.03
==================================================
Note:                 *p<0.1; **p<0.05; ***p<0.01
```

10.2.5 Baseline + education level

This section shows the OMR results when education level is included as one of the dependent variables in the model.

Note that this model fits a smaller dataset because the number of patients that have given their education level is smaller than the full dataset.

Listing 16: Baseline MR model added education level variable.

```
=============================================================
                                            Φ^u
-------------------------------------------------------------
ν^u                                    -0.015*** (0.001)
Ψ^u                                   0.033*** (0.0002)
C(education)Cant read/write                -0.027** (0.014)
C(education)Complete college                -0.007 (0.018)
C(education)Complete elementary           0.037*** (0.013)
C(education)Complete high school             0.003 (0.013)
C(education)Doctoral                        -0.106 (0.132)
C(education)Espec./Residency                 0.009 (0.045)
C(education)Incomplete college               0.004 (0.018)
C(education)Incomplete elementary          0.024** (0.011)
C(education)Incomplete high school          -0.006 (0.014)
C(education)Masters                         -0.050 (0.119)
Constant                                     0.018 (0.011)
-------------------------------------------------------------
Observations                                    61,060
R2                                               0.511
Adjusted R2                                      0.511
Residual Std. Error                     0.602 (df = 61047)
F Statistic                      5,312.884*** (df = 12; 61047)
-------------------------------------------------------------
Model 1: Φ^u ~ ν^u + Ψ^u
Model 2: Φ^u ~ ν^u + Ψ^u + C(education)
  Res.Df   RSS Df Sum of Sq      F    Pr(>F)
1  61057 22127
2  61047 22107 10    19.845 5.4801 3.472e-08 ***
=============================================================
Note:                                *p<0.1; **p<0.05; ***p<0.01
```

10.2.6 Baseline + marital status

This section shows the OMR results when marital status is included as one of the dependent variables in the model.

Listing 17: Baseline MR model added marital status variable.

```
====================================================
                                 Φ^u
----------------------------------------------------
ν^u                          -0.027*** (0.001)
Ψ^u                         0.035*** (0.0002)
C(marital)Divorced              0.105*** (0.025)
C(marital)Ignored              -0.029*** (0.008)
C(marital)Married                -0.005 (0.008)
C(marital)Not informed         -0.072*** (0.008)
C(marital)Separated             0.080*** (0.011)
C(marital)Single                -0.014* (0.008)
C(marital)Widower                0.019* (0.011)
Constant                        0.077*** (0.008)
----------------------------------------------------
Observations                        132,722
R2                                   0.494
Adjusted R2                          0.494
Residual Std. Error          0.495 (df = 132712)
F Statistic            14,420.090*** (df = 9; 132712)
----------------------------------------------------
Model 1: Φ^u ~ ν^u + Ψ^u
Model 2: Φ^u ~ ν^u + Ψ^u + C(marital)
  Res.Df   RSS Df Sum of Sq      F    Pr(>F)
1 132719 32592
2 132712 32464  7    128.13 74.829 < 2.2e-16 ***
====================================================
Note:                     *p<0.1; **p<0.05; ***p<0.01
```

10.2.7 Baseline + average neighborhood income assigned to patients

Listing 18: Baseline MR model added average neighborhood income variable.

```
==================================================
                              Φ^u
--------------------------------------------------
ν^u                    -0.026*** (0.001)
Ψ^u                   0.035*** (0.0002)
avg_income               0.00003*** (0.00000)
Constant                   0.016*** (0.005)
--------------------------------------------------
Observations                   132,722
R2                              0.493
Adjusted R2                     0.493
Residual Std. Error      0.495 (df = 132718)
F Statistic         42,944.890*** (df = 3; 132718)
--------------------------------------------------
Model 1: Φ^u ~ ν^u + Ψ^u
Model 2: Φ^u ~ ν^u + Ψ^u + avg_income
  Res.Df   RSS Df Sum of Sq      F    Pr(>F)
1 132719 32592
2 132718 32582  1    9.9727 40.622 1.853e-10 ***
==================================================
Note:                  *p<0.1; **p<0.05; ***p<0.01
```

10.2.8 Baseline + neighborhood safety variables assigned to patients

Listing 19: Baseline MR model added neighborhood safety variables.

```
==================================================
                              Φ^u
--------------------------------------------------
ν^u                    -0.026*** (0.001)
Ψ^u                   0.035*** (0.0002)
theft_pc                  -0.737*** (0.283)
robbery_p1000               -0.004 (0.003)
suicide_p1000               0.006 (0.009)
transitcrime_p1000         0.022*** (0.002)
traffic_p1000              0.008*** (0.002)
rape_p1000                  -0.002 (0.004)
Constant                   0.024*** (0.004)
--------------------------------------------------
Observations                   132,722
R2                              0.493
Adjusted R2                     0.493
Residual Std. Error      0.495 (df = 132713)
F Statistic         16,148.060*** (df = 8; 132713)
--------------------------------------------------
Model 1: Φ^u ~ ν^u + Ψ^u
Model 2: Φ^u ~ ν^u + Ψ^u + theft_pc +
    robbery_p1000 + suicide_p1000 +
    transitcrime_p1000 + traffic_p1000 +
    rape_p1000
  Res.Df   RSS Df Sum of Sq      F    Pr(>F)
1 132719 32592
2 132713 32538  6    54.096 36.773 < 2.2e-16 ***
==================================================
Note:                  *p<0.1; **p<0.05; ***p<0.01
```

10.2.9 Baseline + neighborhood

Listing 20: Baseline MR model added neighborhood as categorical variables.

```
======================================================
                                  Φ^u
------------------------------------------------------
ν^u                        -0.026*** (0.001)
Ψ^u                       0.035*** (0.0002)
C(hood)BADENFURT                -0.021 (0.014)
C(hood)BOA VISTA                0.009 (0.024)
C(hood)BOM RETIRO             0.150*** (0.036)
```

```
C(hood)CENTRO                    0.012 (0.013)
C(hood)DA GLORIA                 -0.009 (0.013)
C(hood)DO SALTO                  -0.005 (0.016)
C(hood)ESCOLA AGRICOLA         -0.041*** (0.012)
C(hood)FIDELIS                   0.005 (0.013)
C(hood)FORTALEZA               -0.030*** (0.011)
C(hood)FORTALEZA ALTA           -0.029** (0.014)
C(hood)GARCIA                    -0.009 (0.011)
C(hood)ITOUPAVA CENTRAL          0.005 (0.011)
C(hood)ITOUPAVA NORTE           -0.023** (0.011)
C(hood)ITOUPAVA SECA            -0.037** (0.019)
C(hood)ITOUPAVAZINHA             0.012 (0.012)
C(hood)JARDIM BLUMENAU           -0.053 (0.047)
C(hood)NOVA ESPERANCA          -0.055*** (0.014)
C(hood)OTHER                   -0.067*** (0.010)
C(hood)PASSO MANSO               0.025* (0.015)
C(hood)PONTA AGUDA               -0.009 (0.013)
C(hood)PROGRESSO                 -0.006 (0.011)
C(hood)RIBEIRAO FRESCO           0.010 (0.021)
C(hood)SALTO DO NORTE            0.019 (0.015)
C(hood)SALTO WEISSBACH           0.018 (0.018)
C(hood)TESTO SALTO               -0.009 (0.015)
C(hood)TRIBESS                 -0.041*** (0.012)
C(hood)VALPARAISO                -0.015 (0.014)
C(hood)VELHA                     -0.015 (0.011)
C(hood)VELHA CENTRAL             -0.009 (0.013)
C(hood)VELHA GRANDE             -0.031* (0.017)
C(hood)VICTOR KONDER             0.026 (0.024)
C(hood)VILA FORMOSA            -0.225*** (0.053)
C(hood)VILA ITOUPAVA             0.015 (0.017)
C(hood)VILA NOVA               -0.041*** (0.015)
C(hood)VORSTADT                 -0.028** (0.014)
Constant                        0.067*** (0.010)
-----------------------------------------------------
Observations                         132,722
R2                                    0.494
Adjusted R2                           0.494
Residual Std. Error           0.495 (df = 132684)
F Statistic             3,502.150*** (df = 37; 132684)
-----------------------------------------------------
Model 1: Φ^u ~ ν^u + Ψ^u
Model 2: Φ^u ~ ν^u + Ψ^u + C(hood)
  Res.Df   RSS Df Sum of Sq      F    Pr(>F)
1 132719 32592
2 132684 32486 35    106.61 12.441 < 2.2e-16 ***
=====================================================
Note:                      *p<0.1; **p<0.05; ***p<0.01
```

10.3 Linear Mixed-Effect (LMM) models

To be sure there were not nested effects between variables gender and age, we also ran a linear mixed-model (LMM) where variable gender is nested within age. This model accounts for specific differences within patient age and across different genders. Intuitively, if there are large variations in the number of interaction that are explained by the nestedness of gender and age, these groups would help explain a large portion of the variance in the data. The results indicate that is not the case. In fact, the variance attributed to gender (within age) group is 0.00016 while the age group is a little higher, 0.00217.

Listing 21: Linear Mixed Model with age nested within gender.

```
Linear mixed model fit by maximum likelihood  ['lmerMod']
ForFormula: Φ^u ~ ν^u + Ψ^u + (1 | age/gender)
   Data: data

     AIC      BIC   logLik deviance df.resid
189314.1 189372.9 -94651.1 189302.1   132716

Scaled residuals:
     Min       1Q   Median       3Q      Max
-13.1102  -0.2048  -0.0734   0.0394  19.2402

Random effects:
 Groups     Name        Variance  Std.Dev.
 gender:age (Intercept) 0.0001645 0.01282
 age        (Intercept) 0.0021678 0.04656
```

```
 Residual                0.2432636 0.49322
Number of obs: 132722, groups:  gender:age, 213; age, 109

Fixed effects:
              Estimate Std. Error t value
(Intercept)  0.0483215  0.0055929    8.64
νᵘ  -0.0262493  0.0007287  -36.02
Ψᵘ  0.0343219  0.0001590  215.87

Correlation of Fixed Effects:
           (Intr) νᵘ
νᵘ  -0.367
Ψᵘ  0.224 -0.831
```

To be sure that neighborhood did not differ in their DDI observations, we also ran a linear mixed-model (LMM) with neighborhood as a random effect. Intuitively, if there are variations in the number of interactions between neighborhoods that cannot be explained by the independent variables alone—due to, say, differences in policies or practices—we should see the random effect variable explaining a great deal of the variance in the model. Our results indicate that is not the case. In fact, the variance attributed to the neighborhood random effect is 0.00059 and therefore too small. This shows that at least in predicting the number of DDI, there is neighborhood homogeneity in how they are being prescribed and thus dispensed.

Listing 22: Linear Mixed Model with neighborhood as random effect.

```
Linear mixed model fit by maximum likelihood  ['lmerMod']
Formula: Φᵘ ~ νᵘ + Ψᵘ + (1 | hood)
   Data: data

     AIC      BIC   logLik deviance df.resid
189980.7 190029.6 -94985.3 189970.7   132717

Scaled residuals:
     Min       1Q   Median       3Q      Max
-13.1462  -0.1846  -0.0678   0.0180  19.1046

Random effects:
 Groups   Name        Variance  Std.Dev.
 hood     (Intercept) 0.0005935 0.02436
 Residual             0.2448642 0.49484
Number of obs: 132722, groups:  hood, 36

Fixed effects:
              Estimate Std. Error t value
(Intercept)   0.0544948  0.0050850   10.72
νᵘ   -0.0264618  0.0007270  -36.40
Ψᵘ  0.0348255  0.0001572  221.58

Correlation of Fixed Effects:
           (Intr) νᵘ
νᵘ  -0.411
Ψᵘ  0.268 -0.841
```

Supplementary Note 11 Patient classification

We applied machine learning classifiers in order to predict if a specific patient had at least one DDI in the whole 18 month period. A binary classification task. Support Vector Machine (SVM)[34] and Logistic Regression (LR)[35] are considered both standard and reliable machine learning algorithm for binary classification problems. We built models for each classifier considering different sets of features, including demographic (i.e., age & gender) and drugs the patient was prescribed in the period. For baseline comparison we also ran against three null model classifiers. One with a "coin-toss" probability of classification (Uniform), another with a bias with respect to class probability (Biased), and a custom made (AgeGender) which finds the best age cutoff for each gender from which it consider all patients older than the cutoff as having a DDI. Regression and classification models were computed using R and Python [36].

We present results as measures derived from a confusion matrix, also called a contingency table[37]. The confusion matrix contains four categories: true positives (TP), patients correctly labeled as having a DDI; false positives (FP), patients incorrectly classified as having a DDI; true negative (TN), patients correctly labeled as not having a DDI; and finally false negatives (FN), patients with DDI but mislabeled as not having them. A contingency table example can be seen in Table 36.

From the confusion matrix we compute Precision and Recall as

$$\text{Precision} = \frac{TP}{TP + FP} \quad , \quad \text{Recall} = \frac{TP}{TP + FN} \quad , \tag{23}$$

where Precision is the fraction of patients with DDI correctly predicted, among all predicted patients with DDI; while Recall is the fraction of patients with DDI correctly predicted, among all patients with DDI. We also compute True Positive Rate (TPR) and False Positive Rate (FPR) measures as

$$TPR = \frac{TP}{TP + FN} \quad , \quad FPR = \frac{FP}{FP + TN} \quad , \tag{24}$$

where TPR measures the fraction of patients with DDI that are correctly classified and FPR measures the fraction of patients with no DDI incorrectly classified as having DDI. These four measures enables the plotting of the Receiver Operator Characteristic (ROC) and the Precision and Recall (P/R) space. In ROC space we plot FPR against TPR while in P/R space we plot Precision against Recall (Figure 15 displays the results). These plots are typically generated to evaluate the performance of machine learning algorithms, and to enable system users to inspect the trained algorithm's precision at a specific recall level, for example. From both ROC and P/R curves we computes their respective interpolated area under the curve (AUC)[37].

From Precision and Recall we also compute the F_1-score (also called F-score or F-measure) as

$$F_1 = 2 \times \frac{\text{Precision} \times \text{Recall}}{\text{Precision} + \text{Recall}} \quad . \tag{25}$$

We also compute Matthew's Correlation Coefficient (MCC)[38], which is regarded as an ideal measure of the quality of binary classification in unbalanced scenarios[39], as

$$MCC = \frac{TP \times TN - FP \times FN}{\sqrt{(TP + FP) + (TP + FN) + (TN + FP) + (TN + FN)}} \quad . \tag{26}$$

Below, we display results as measures of Precision, Recall, F_1-score, MCC, AUC ROC curve and AUC P/R curve.

We also display the full table of feature weights for both classifiers. Since these are both linear classifiers,

	DDI	no DDI
predicted DDI	TP	FP
predicted no DDI	FN	TN

Supplementary Table 36: Confusion Matrix.

one can interpret positive (negative) values as contributing to the positive (negative) class—having a DDI. The higher (smaller) the weight, the bigger (smaller) the contribution. All results are based on 4-fold cross validation.

† Gender is used as a categorical variable and expanded into the binary features ($g = M$ and $g = F$).

‡ Education level is used as a categorical variable expanded into individuals binary features (“Cant read/write”, “Can read/write a note”, “Incomplete elementary”, “Complete elementary”, “Incomplete high school”, “Complete high school”, “Incomplete college”, “Complete college”, “Espec./Residency”, “Masters”, and “Doctoral”).

11.1 Simple model

Patients: 132,722

- **DDI (positive):** 15,527 (11.70%)
- **no DDI (negative):** 117,195 (88.30%)

Features: 127

- **Demographic:** gender† (g), age (y), number of drugs (ν^u), number of co-administrations (Ψ^u).
- **Neighborhood:** average income, number of thefts per capita, number of robberies per capita, number of suicides per capita, number of transit crimes per capita, number of traffic accidents per capita, number of rapes per capita.
- **Drug:** all drugs D.

Fold	Precision	Recall	F_1	MCC	AUC ROC	AUC P/R
1	0.8196	0.6309	0.7130	0.6877	0.9676	0.8269
2	0.8241	0.6494	0.7264	0.7011	0.9702	0.8365
3	0.8127	0.6504	0.7226	0.6957	0.9697	0.8315
4	0.8187	0.6436	0.7207	0.6949	0.9690	0.8311
Mean	0.8188	0.6436	0.7207	0.6948	0.9691	0.8315

Supplementary Table 37: Individual fold and mean performance of Support Vector Machine (SVM) classifier on stratified 4-fold cross-validation, using demographic and drug features. Measures of performance shown are: Precision, Recall, F1 (balanced Precision and Recall), Matthew’s Correlation Coefficient, the Area Under the Receiver Operating Characteristic Curve, and the Area Under the Precision and Recall Curve.

Fold	Precision	Recall	F_1	MCC	AUC ROC	AUC P/R
1	0.8085	0.6535	0.7228	0.6953	0.9675	0.8249
2	0.8096	0.6669	0.7314	0.7037	0.9700	0.8337
3	0.7991	0.6662	0.7266	0.6977	0.9697	0.8299
4	0.8092	0.6612	0.7277	0.7002	0.9691	0.8304
Mean	0.8066	0.6619	0.7271	0.6992	0.9691	0.8297

Supplementary Table 38: Individual fold and mean performance of Logistic Regression (LR) classifier on stratified 4-fold cross-validation, using demographic and drug features. Measures of performance shown are: Precision, Recall, F1 (balanced Precision and Recall), Matthew’s Correlation Coefficient, the Area Under the Receiver Operating Characteristic Curve, and the Area Under the Precision and Recall Curve.

11.2 Complete model

Patients: 132,722

- **DDI (positive):** 15,527 (11.70%)
- **no DDI (negative):** 117,195 (88.30%)

Features: 154

Classifier	Precision	Recall	F_1	MCC	AUC ROC	AUC P/R
Uniform	0.1181	0.5075	0.1916	0.0035	0.5	0.5585
Biased	0.1147	0.1153	0.1150	-0.0026	0.4987	0.1668
GenderAge	0.2044	0.8834	0.3320	0.2751	0.7139	0.5507

Supplementary Table 39: Mean performance of Uniform (coin-toss), Biased (biased coin-toss on class distribution) and GenderAge (hard cutoff for gender and gender) classifiers on stratified 4-fold cross-validation, using demographic and drug features. Measures of performance shown are: Precision, Recall, F1 (balanced Precision and Recall), Matthew's Correlation Coefficient, the Area Under the Receiver Operating Characteristic Curve, and the Area Under the Precision and Recall Curve.

Demographic: gender† (g), age (y), number of drugs (ν^u), number of co-administrations (Ψ^u), education levels‡.

Neighborhood: average income, number of thefts per capita, number of robberies per capita, number of suicides per capita, number of transit crimes per capita, number of traffic accidents per capita, number of rapes per capita.

Drug: all drugs D.

Classifier	Precision	Recall	F_1	MCC	AUC ROC	AUC P/R
SVM	0.8186	0.6442	0.7210	0.6951	0.9690	0.8312
LR	0.8070	0.6619	0.7273	0.6994	0.9690	0.8295

Supplementary Table 40: Mean performance of classifiers on stratified 4-fold cross-validation, using all possible features, including demographic, neighborhood and drugs dispensed. Measures of performance shown are: Precision, Recall, F1 (balanced Precision and Recall), Matthew's Correlation Coefficient, the Area Under the Receiver Operating Characteristic Curve, and the Area Under the Precision and Recall Curve.

11.3 No Drugs model

This model is similar to the "simple" model, except no drug features are used.

Patients: 132,722

DDI (positive): 15,527 (11.70%)

no DDI (negative): 117,195 (88.30%)

Features: 5

Demographic: gender† (g), age (y), number of drugs (ν^u), number of co-administrations (Ψ^u).

Neighborhood: None.

Drug: None.

Classifier	Precision	Recall	F_1	MCC	AUC ROC	AUC P/R
SVM	0.7578	0.3791	0.5053	0.4971	0.9185	0.6539
LR	0.7172	0.4170	0.5273	0.5044	0.9130	0.6391

Supplementary Table 41: Mean performance of classifiers on stratified 4-fold cross-validation, using only demographic features. Measures of performance shown are: Precision, Recall, F1 (balanced Precision and Recall), Matthew's Correlation Coefficient, the Area Under the Receiver Operating Characteristic Curve, and the Area Under the Precision and Recall Curve.

11.4 Precision & Recall and Receiver Operating Characteristic curves

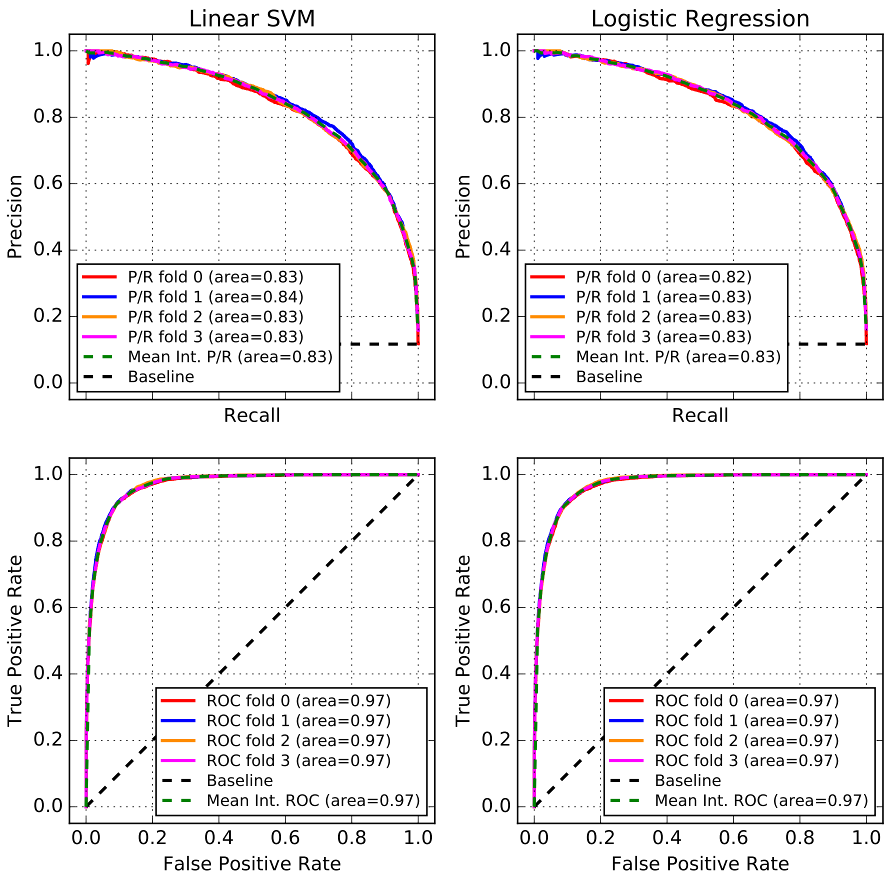

Supplementary Figure 15: Precision and Recall (P/R) curve and Receiver operating characteristic (ROC) curve for individual cross-validation folds. Model containing demographic and drug features. Black and green dotted line shows the baseline and mean values, respectively.

11.5 Feature loadings

Supplementary Tables 42 and 43 shows the feature loading for both SVM and LR classifiers on model "simple".

feature	coef	feature	coef
d=Digoxin	1.1677	d=Acetaminophen	-0.1169
d=Diltiazem	0.8718	d=Tobramycin	-0.1185
d=Warfarin	0.6938	d=Hydrochlorothiazide	-0.1203
d=Haloperidol	0.6879	d=Norethisterone	-0.1225
d=Glyburide	0.6681	d=Propylthiouracil	-0.1242
d=Pyrimethamine	0.6549	d=Phenylephrine	-0.1309
d=Phenytoin	0.6015	d=Estrogens Conjugated	-0.1312
d=Biperiden	0.5807	d=Trimethoprim	-0.1325
d=Carbamazepine	0.5752	d=Sulfamethoxazole	-0.1325
d=Gliclazide	0.4735	d=Colchicine	-0.1333
d=Clonazepam	0.4717	d=Diclofenac	-0.1347
d=Methyldopa	0.4617	d=Ranitidine	-0.1374
d=Propranolol	0.4487	d=Neomycin	-0.1383
d=Lithium	0.3887	d=Bacitracin	-0.1383
d=Fluconazole	0.3716	d=Nimesulide	-0.1413
ν_i	0.3169	d=Fenoterol	-0.1446
d=Acetylsalicylic Acid	0.3119	d=Nystatin	-0.1508
$\Psi_{i,j}$	0.3080	d=Albendazole	-0.1514
d=Diazepam	0.3038	d=Nitrofurantoin	-0.1514
d=Omeprazole	0.2822	d=Loratadine	-0.1611
d=Amitriptyline	0.2810	d=Metamizole	-0.1624
d=Iron (II) Sulfate	0.2584	d=Spironolactone	-0.1634
d=Ethinyl Estradiol	0.2571	d=Tramadol	-0.1643
d=Ibuprofen	0.2170	d=Dexchlorpheniramine maleate	-0.1664
d=Imipramine	0.1825	d=Enalapril	-0.1671
d=Fluoxetine	0.1639	d=Azithromycin	-0.1672
d=Verapamil	0.1455	d=Miconazole	-0.169
d=Timolol	0.1452	d=Scopolamine butylbromide	-0.171
d=Atenolol	0.1432	d=Metronidazole	-0.1747
d=Nortriptyline	0.1159	d=Cephalexin	-0.1767
d=Doxycycline	0.1046	d=Ipratropium Bromide	-0.1779
d=Nifedipine	0.0973	d=Hydrocortisone	-0.1812
d=Methylphenidate	0.0638	d=Metoclopramide	-0.1832
d=Vaseline	0.0596	d=Levodopa	-0.1872
y	0.0518	d=Medroxyprogesterone Acetate	-0.1877
d=Phenobarbital	0.0274	d=Doxazosin	-0.1909
d=Prednisone	0.0232	d=Amlodipine	-0.1936
d=Estradiol	0.0181	d=Losartan	-0.1937
d=Atropine	0.0000	d=Metformin	-0.1943
d=Thiocolchicoside	0.0000	d=Mebendazole	-0.1945
d=Salbutamol	-0.0071	d=Fluphenazine	-0.204
d=Dexamethasone	-0.0102	d=Captopril	-0.2041
d=Penicillin G procaine	-0.0115	d=Amiodarone	-0.2042
d=Simvastatin	-0.0191	d=Bromazepam	-0.2063
d=Gentamicin	-0.0229	d=Codeine	-0.2064
d=Epinephrine	-0.0347	d=Valproic acid	-0.2083
d=Furosemide	-0.0395	d=Penicillin G Benzathine	-0.2123
d=Carvedilol	-0.0544	d=Aminophylline	-0.2133
d=Erythromycin	-0.0588	d=Clavulanate	-0.2141
d=Chlorpromazine	-0.0605	d=Clopidogrel	-0.2162
d=Methotrimeprazine	-0.0683	d=Carbidopa	-0.2269
d=Morphine	-0.0759	d=Insulin	-0.246
d=Levothyroxine	-0.0776	d=Isosorbide Mononitrate	-0.269
d=Alendronate	-0.0820	d=Nicotine	-0.3003
d=Amoxicillin	-0.0908	d=Glucose	-0.305
d=Ciprofloxacin	-0.0937	$g = M$	-0.3193
d=Prednisolone	-0.0944	$g = F$	-0.3213
d=Permethrin	-0.0978	d=Sodium chloride	-0.3474
d=Levonorgestrel	-0.0982	d=Isosorbide Dinitrate	-0.351
d=Folic acid	-0.0983	d=Oseltamivir	-0.3643
d=Promethazine	-0.1059	d=Betamethasone	-0.4765
d=Maprotiline	-0.1073	d=Spiramycin	-0.521
d=Norfloxacin	-0.1100	d=Sulfadiazine	-0.5259
d=Allopurinol	-0.1148	-	-

Supplementary Table 42: Feature weights for Support Vector Machine (SVM) classifier on model "simple".

feature	coef	feature	coef
d=Digoxin	3.6826	d=Norethisterone	-0.4217
d=Diltiazem	2.7678	d=Amoxicillin	-0.4283
d=Haloperidol	2.3874	d=Promethazine	-0.4327
d=Warfarin	2.3423	d=Colchicine	-0.434
d=Glyburide	2.2139	d=Hydrochlorothiazide	-0.4526
d=Phenytoin	2.1363	d=Norfloxacin	-0.4545
d=Carbamazepine	2.1098	d=Estrogens Conjugated	-0.4683
d=Biperiden	1.9247	d=Tobramycin	-0.4763
d=Clonazepam	1.6984	d=Propylthiouracil	-0.4767
d=Methyldopa	1.6363	d=Trimethoprim	-0.4888
d=Propranolol	1.5735	d=Sulfamethoxazole	-0.4888
d=Gliclazide	1.5618	d=Acetaminophen	-0.502
ν_i	1.4941	d=Spiramycin	-0.506
d=Fluconazole	1.3668	d=Ranitidine	-0.5178
d=Lithium	1.3303	d=Diclofenac	-0.5242
d=Acetylsalicylic Acid	1.0479	d=Betamethasone	-0.5301
d=Diazepam	1.0178	d=Nimesulide	-0.5316
d=Omeprazole	1.0114	d=Neomycin	-0.5318
d=Amitriptyline	0.9684	d=Bacitracin	-0.5318
d=Iron (II) Sulfate	0.8905	d=Nystatin	-0.5508
$\Psi_{i,j}$	0.7721	d=Prednisolone	-0.5531
d=Ibuprofen	0.7282	d=Fenoterol	-0.5542
d=Pyrimethamine	0.6518	d=Spironolactone	-0.564
d=Fluoxetine	0.6245	d=Hydrocortisone	-0.5778
d=Imipramine	0.6188	d=Mebendazole	-0.5857
d=Atenolol	0.5100	d=Enalapril	-0.5955
d=Ethinyl Estradiol	0.4965	d=Albendazole	-0.5991
d=Verapamil	0.3885	d=Nitrofurantoin	-0.6128
d=Doxycycline	0.3681	d=Miconazole	-0.6173
y	0.3547	d=Ipratropium Bromide	-0.619
d=Timolol	0.3492	d=Loratadine	-0.6196
d=Nortriptyline	0.3217	d=Metamizole	-0.6206
d=Nifedipine	0.2797	d=Scopolamine butylbromide	-0.6347
d=Levonorgestrel	0.2220	d=Tramadol	-0.6364
d=Phenobarbital	0.1465	d=Metronidazole	-0.6476
d=Vaseline	0.1118	d=Dexchlorpheniramine maleate	-0.6534
d=Estradiol	0.0873	d=Medroxyprogesterone Acetate	-0.6733
d=Prednisone	0.0824	d=Metformin	-0.6762
d=Epinephrine	0.0357	d=Azithromycin	-0.6796
d=Erythromycin	0.0242	d=Captopril	-0.6855
d=Thiocolchicoside	-0.0044	d=Losartan	-0.6882
d=Atropine	-0.0128	d=Amlodipine	-0.6899
d=Sulfadiazine	-0.0250	d=Cephalexin	-0.6901
d=Penicillin G procaine	-0.0593	d=Doxazosin	-0.6929
d=Salbutamol	-0.0845	d=Metoclopramide	-0.7212
d=Phenylephrine	-0.0869	d=Aminophylline	-0.7297
d=Simvastatin	-0.0914	d=Codeine	-0.7311
d=Dexamethasone	-0.0917	d=Clopidogrel	-0.7375
d=Gentamicin	-0.1000	d=Amiodarone	-0.7402
d=Methylphenidate	-0.1019	d=Clavulanate	-0.7449
d=Sodium chloride	-0.1856	d=Valproic acid	-0.7461
d=Fluphenazine	-0.2091	d=Carbidopa	-0.7552
d=Furosemide	-0.2152	d=Bromazepam	-0.7571
d=Methotrimeprazine	-0.2171	d=Levodopa	-0.7619
d=Carvedilol	-0.2223	d=Penicillin G Benzathine	-0.8072
d=Chlorpromazine	-0.2356	d=Insulin	-0.8443
d=Maprotiline	-0.2791	d=Isosorbide Mononitrate	-0.9186
d=Morphine	-0.2889	d=Nicotine	-0.9342
d=Levothyroxine	-0.2929	d=Glucose	-0.9742
d=Folic acid	-0.3650	$g = M$	-1.116
d=Alendronate	-0.3683	$g = F$	-1.132
d=Allopurinol	-0.3954	d=Isosorbide Dinitrate	-1.178
d=Permethrin	-0.4101	d=Oseltamivir	-1.3
d=Ciprofloxacin	-0.4119	-	-

Supplementary Table 43: Feature weights on Logistic Regression (LR) classifier on model "simple".

Supplementary References

1. Paim, J., Travassos, C., Almeida, C., Bahia, L. & Macinko, J. The Brazilian health system: history, advances, and challenges. *The Lancet* **377,** 21–27 (May 2011).
2. Ministério da Saúde. *Final report of the 14th National Health Conference* in *Conferência Nacional de Saúde* (Brasília, 2012). (2015).
3. Da Cunha, R. E. National Health Card – the implementation and conception challenges of a national system used to extract information from health services. *Ciência e Saúde Coletiva* **7,** 869–878 (2002).
4. Hexsel, R. A., Urban, A. E. & Barros, R. S. M. *Arquitetura do Sistema Cartão Nacional de Saúde* in *VIII Congresso Brasileiro de Informática em Saúde* (Sept. 2002).
5. Franco, T. B. *Processos de trabalho e transição tecnologica na saude: um olhar a partir do Sistema Cartão Nacional de Saude* Doctorate (Faculdade de Ciências Médicas, Campinas, SP, 2003).

6. Lorenzetti, J., de Melo Lanzoni, G. M., Assuiti, L. F. C., de Pires, D. E. P. & Ramos, F. R. S. Gestão em Saúde no Brasil: Diálogo com Gestores Públicos e Privados. *Texto Contexto Enferm* **23,** 417–425 (Apr. 2014).

7. IBGE–Instituto Brasileiro de Geografia e Estatística. ibge.gov.br. 2019.

8. Laboratório de Desenvolvimento e Transferência de Tecnologia. *Pronto: nosso plano é atender você* http://www.furb.br/ldtt/. 2019.

9. Choi, B. C. K. & Pak, A. W. P. Multidisciplinarity, interdisciplinarity and transdisciplinarity in health research, services, education and policy: 1. Definitions, objectives, and evidence of effectiveness. *Send to Clin Invest Med* **29,** 351–64 (Dec. 2006).

10. Etzkowitz, H. *The Triple Helix: University-Industry-Government Innovation in Action* (Taylor & Francis, 2010).

11. De Araújo, L. P., Berkenbrock, C. D. M. & Mattos, M. M. *Using participatory design in designing phase of collaborative systems* in *Proc. of the 2014 IEEE 18th Inte'l Conf. on Comp. Supported Cooperative Work in Design (CSCWD)* (Hsinchu, Taiwan, May 2014), 633–637.

12. De Araújo, L. P., Mattos, M. M., Casa, S. M., Boeing, E. & da Costa, S. E. *Mobile Application to Support Community Health Agents using GeneXus Smart Devices* in *Proc. of the 2015 Inter'l Conf. on Innovative Mobile and Internet Services in Ubiquitous Computing (IMIS)* (Blumenau, Brazil, July 2015).

13. Mattos, M. M. *et al. PRONTO System: integration between doctors and pharmacists in the basic health care* in *Int'l Conf. Software Eng. Research and Practice, SERP'15* (Las Vegas, NV, July 2015), 177–180.

14. Ministério da Saúde. *National Primary Health Care Policy* 1st ed., 110. http://189.28.128.100/dab/docs/publicacoes/geral/pnab.pdf (Ministério da Saúde, Brasília, 2012).

15. Lancet, T. Making sense of our digital medicine Babel. *The Lancet* **392,** 1487 (Nov. 2018).

16. Rocha, L. M., Simas, T., Rechtsteiner, A., Giacomo, M. D. & Luce, R. *MyLibrary@LANL: Proximity and Semi-metric Networks for a Collaborative and Recommender Web Service* in *2005 IEEE/WIC/ACM International Conference on Web Intelligente (WI'05)* (IEEE Press, 2005), 565–571.

17. Simas, T. & Rocha, L. M. Distance closures on complex networks. *Network Science* **3,** 227–268 (June 2015).

18. Correia, R. B., Li, L. & Rocha, L. M. Monitoring potential drug interactions and reactions via network analysis of Instagram user timeliness. *Pacific Symposium on Biocomputing* **21,** 492–503 (2016).

19. *Drugs.com* http://www.drugs.com. Accessed on May 23. 2019.

20. Wishart, D. *et al.* DrugBank: a knowledgebase for drugs, drug actions and drug targets. *Nucleic Acids Res* **36,** D901–6 (Jan. 2008).

21. Hamilton, R. A., Briceland, L. L. & Andritz, M. H. Frequency of Hospitalization after Exposure to Known Drug-Drug Interactions in a Medicaid Population. *Pharmacotherapy: The Journal of Human Pharmacology and Drug Therapy* **18,** 1112–1120 (1998).

22. DN, J., M, M., A, K., A, L. & DA, R. Drug-drug interactions among elderly patients hospitalized for drug toxicity. *JAMA* **289,** 1652–1658 (2003).

23. Becker, M. L. *et al.* Hospitalisations and emergency department visits due to drug–drug interactions: a literature review. *Pharmacoepidemiology and Drug Safety* **16,** 641–651 (2007).

24. Wu, C., Bell, C. M. & Wodchis, W. P. Incidence and Economic Burden of Adverse Drug Reactions among Elderly Patients in Ontario Emergency Departments: A Retrospective Study. *Drug Safety* **35,** 769–781 (2012).

25. Ministério da Saúde. *Sistema de Informações Hospitalares–SIH/SUS* datasus.saude.gov.br. Accessed on May 23. 2019.

26. Patrignani, A. *et al.* Under-reporting of adverse drug reactions, a problem that also involves medicines subject to additional monitoring. Preliminary data from a single-center experience on novel oral anticoagulants. *Giornale italiano di cardiologia (Rome)* **19,** 54–61 (Jan. 2018).

27. González-Rubio, F. *et al.* Underreporting of recognized adverse drug reactions by primary care physicians: an exploratory study. *Pharmacoepidemiology and drug safety* **20,** 1287–1294 (2011).

28. Ponte, M., Carrara, R., Flores Lazdin, C. & Wachs, A. *Drug-Drug Interactions: An Under-Estimated Problem* in *Drug Safety* **33** (2010), 894–894.

29. Alvarez, P. A. *et al.* Adverse drug reactions as a reason for admission to an internal medicine ward in Argentina. *International Journal of Risk & Safety in Medicine* **25,** 185–192 (2013).

30. Tatonetti, N. P., Fernald, G. H. & Altman, R. B. A novel signal detection algorithm for identifying hidden drug-drug interactions in adverse event reports. *Journal of the American Medical Informatics Association* **19,** 79–85 (June 2012).

31. Iyer, S. V., Harpaz, R., LePendu, P., Bauer-Mehren, A. & Shah, N. H. Mining clinical text for signals of adverse drug-drug interactions. *Journal of the American Medical Informatics Association* **21,** 353–362 (Mar. 2014).

32. Cano, F. G. & Rozenfeld, S. Adverse drug events in hospitals: a systematic review. *Cadernos de Saúde Pública* **25,** S360–S372 (2009).

33. Woltman, H., Feldstain, A., MacKay, J. C. & Rocchi, M. An introduction to hierarchical linear modeling. *Tutorials in Quantitative Methods for Psychology* **8,** 52–69 (2012).

34. Boser, B. E., Guyon, I. M. & Vapnik, V. N. *A Training Algorithm for Optimal Margin Classifiers* in *Proceedings of the Fifth Annual Workshop on Computational Learning Theory* (ACM, Pittsburgh, PA, 1992), 144–152. doi:10.1145/130385.130401.

35. Cox, D. in *Research Papers in Probability and Statistics (Festschriftfor J. Neyman)* (ed David, F.) 55–71 (Wiley, London, 1966).

36. Pedregosa, F. *et al.* Scikit-learn: Machine Learning in Python. *Journal of Machine Learning Research* **12,** 2825–2830 (2011).

37. Davis, J. & Goadrich, M. *The Relationship Between Precision-Recall and ROC Curves* in *Proceedings of the 23rd International Conference on Machine Learning* (ACM, New York, NY, USA, June 2006), 233–240.

38. Matthews, B. Comparison of the predicted and observed secondary structure of T4 phage lysozyme. *Biochimica et Biophysica Acta (BBA) - Protein Structure* **405,** 442–451 (1975).

39. Baldi, P., Brunak, S., Chauvin, Y., Andersen, C. A. F. & Nielsen, H. Assessing the accuracy of prediction algorithms for classification: an overview. *Bioinformatics* **16,** 412–424 (May 2000).
